# Supplementary material for: Design-driven optimization of low-cost reagent formulations for reproducible and high-yielding cell-free gene expression
Source: Nat Commun. 2026 Mar 5;17:3478. doi: 10.1038/s41467-026-69605-8 (PMC13079839; doi:10.1038/s41467-026-69605-8)
Supplement: Supplementary file 1 — Supplementary Information [file 41467_2026_69605_MOESM1_ESM.pdf]

Supplementary Materials for:

Design-driven optimization of low-cost reagent formulations for reproducible and high-yielding cell-free gene expression

Meagan L. Olsen, Caroline E. Copeland, Chad A. Sundberg, Rochelle Aw, Zachary M. Shaver, Govind Rao, James R. Swartz, Ashty S. Karim, & Michael C. Jewett

This PDF file contains:

Supplementary Figures 1-25

Supplementary Tables 1-4

All relevant data is provided in the Source Data or Supplementary Data files.

Supplementary Figures

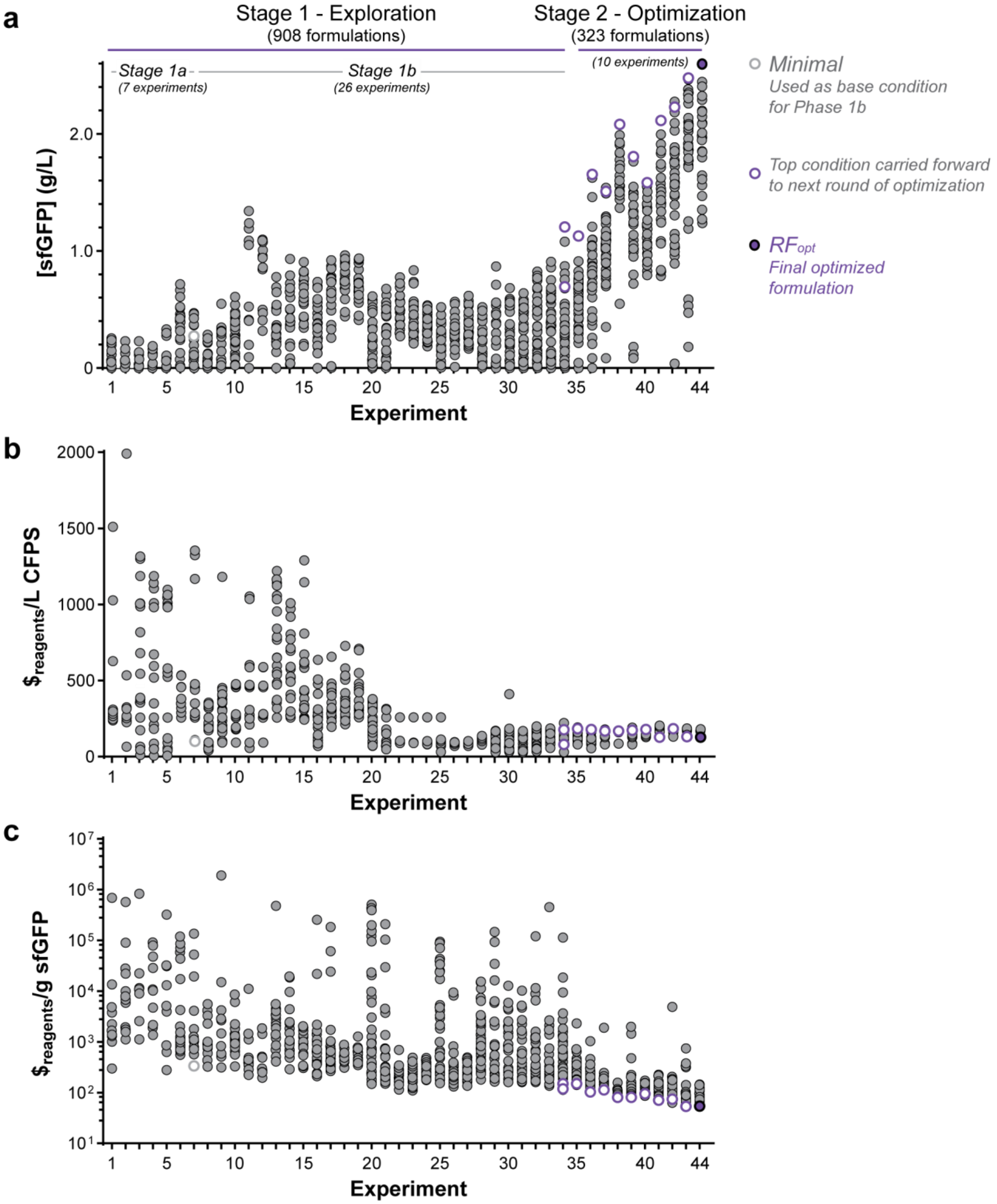

Supplementary Figure 1. Shift in cell-free reaction yield and cost over the optimization campaign. sfGFP yields (a), $\$_{reagents}$/L cell-free protein synthesis (b), and $\$_{reagents}$/g sfGFP (c) over the course of the reagent optimization campaign.

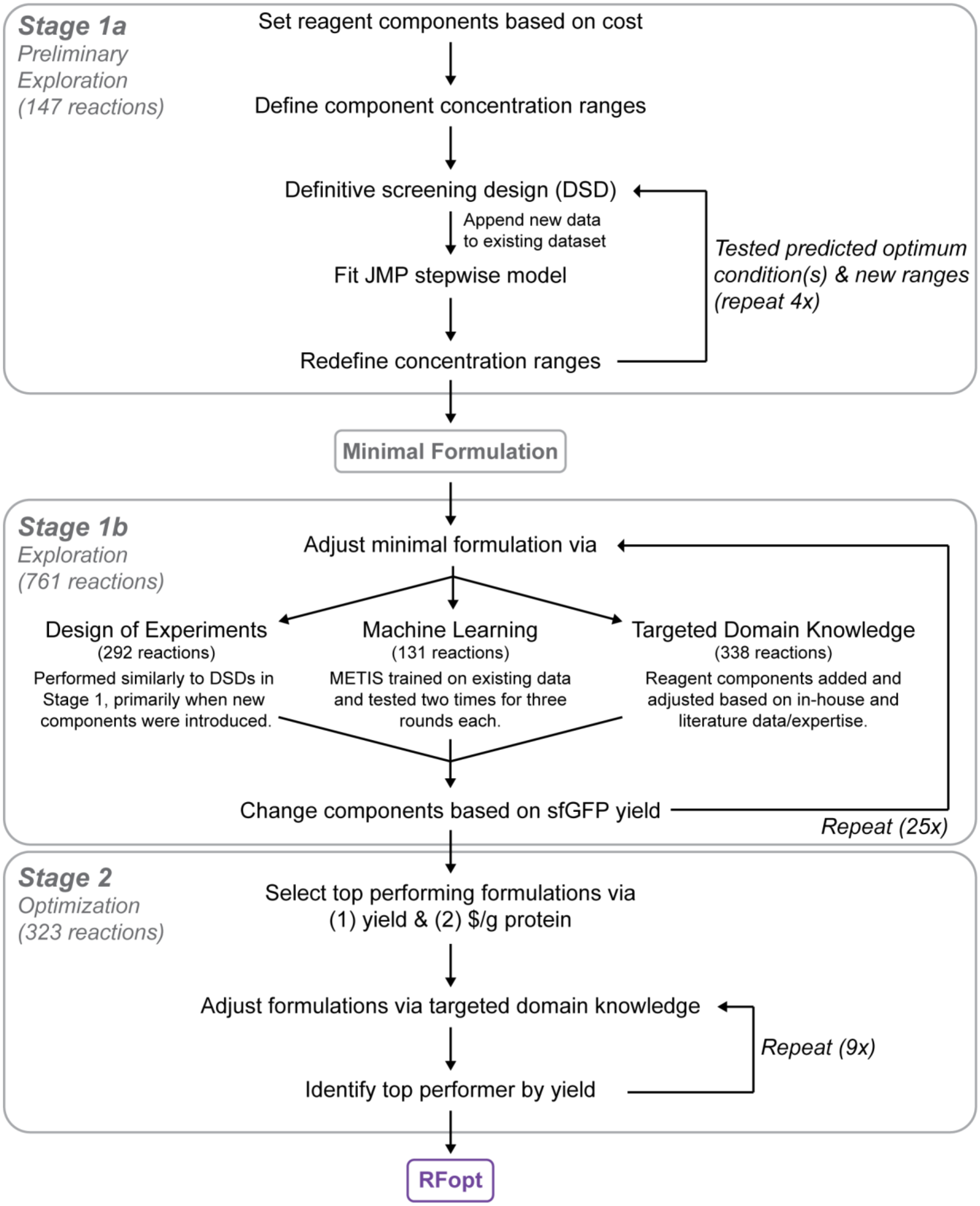

Supplementary Figure 2. Optimization campaign strategies. In some cases, additional high-performing formulations were re-tested across multiple experiments to determine consistency. Additionally, during stage 2, several top performers rather than a single top performer were advanced based on performance consistency and/or domain knowledge, such as the design rules learned during Stage 1.

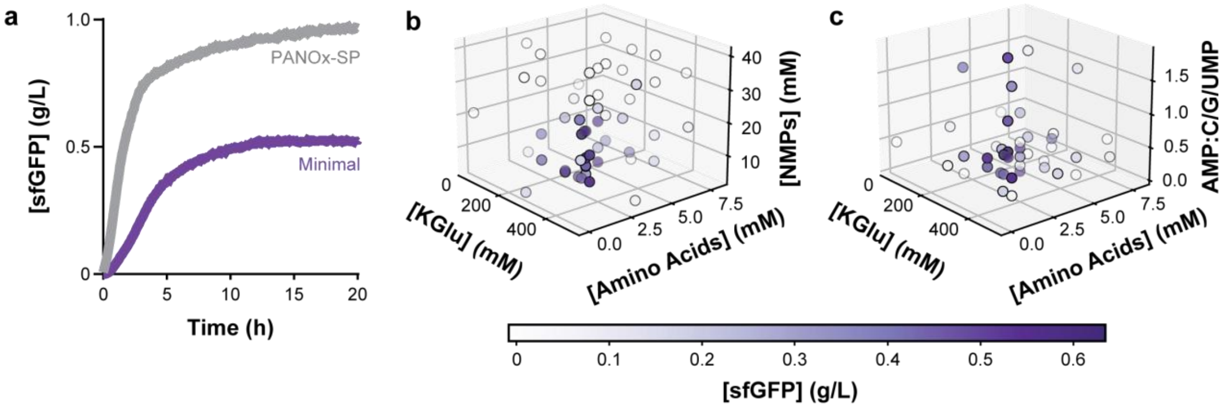

Supplementary Figure 3. Minimal reagent formulation behavior. (a) Cell-free sfGFP expression levels using either the PANOx-SP or minimal reagent formulations. Data is presented as mean ± standard deviation of n = 3 replicates. (b) sfGFP yield from manipulating the concentration of the three reagent components in the minimal formulation. The scatterplot shows potassium glutamate (KGlu), amino acids, and total NMP concentration, consisting of the sum of AMP, CMP, GMP, and UMP concentrations. (c) Reagent concentrations and sfGFP yield from (b), re-plotted to show the AMP to CMP/GMP/UMP concentration ratios. All reactions were run with BL21 Star (DE3) lysate at 30 °C for 20 h.

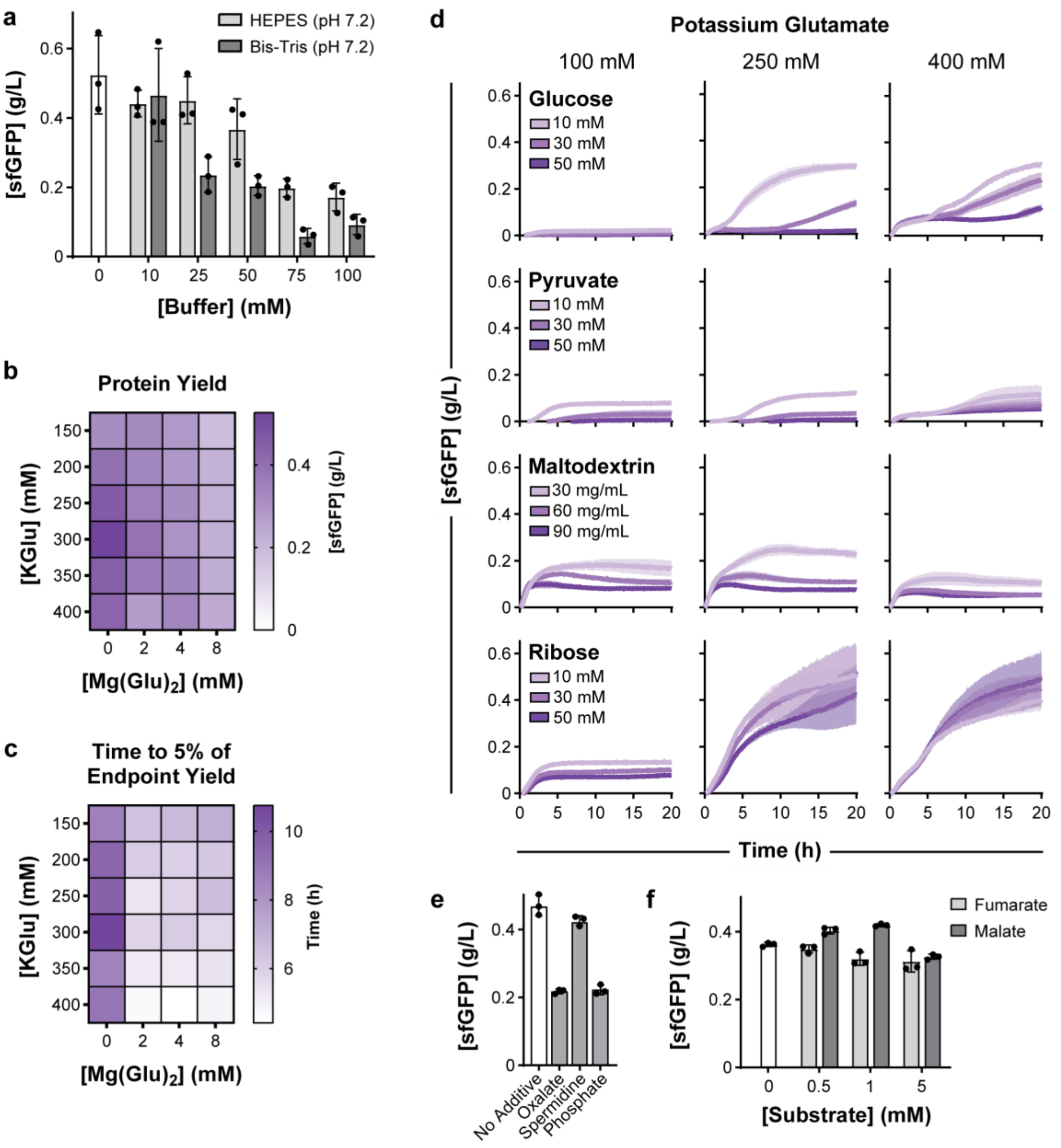

Supplementary Figure 4. Reagent addition to the minimal system. (a) sfGFP expression levels resulting from addition of either HEPES or Bis-Tris buffer, both at pH 7.2, to the minimal system. Data is presented as mean ± standard deviation of n = 3 replicates. (b) Magnesium and potassium glutamate cross-titration and corresponding impact on sfGFP expression level. Heat map cells show the average of n = 3 replicates. (c) Impact of magnesium and potassium glutamate cross-titration on the time the cell-free expression reaction took to reach within 5% of the final 20-h reaction yield. (d) Time courses of sfGFP production after adding alternative energy substrates and different amounts of potassium glutamate to the minimal reagent formulation. The shaded area represents the standard deviation of n = 3 replicates. (e) sfGFP expression levels when including either 4 mM oxalate, 1.5 mM spermidine, or 15 mM phosphate in the minimal reagent formulation. (f) sfGFP expression levels when including either fumarate or malate, two tricarboxylic acid cycle intermediates, in the reagent formulation. Data in (e) and (f) is presented as mean ± standard deviation of n = 3 replicates. All reactions were run using BL21 Star (DE3) lysate at 30 °C for 20 h.

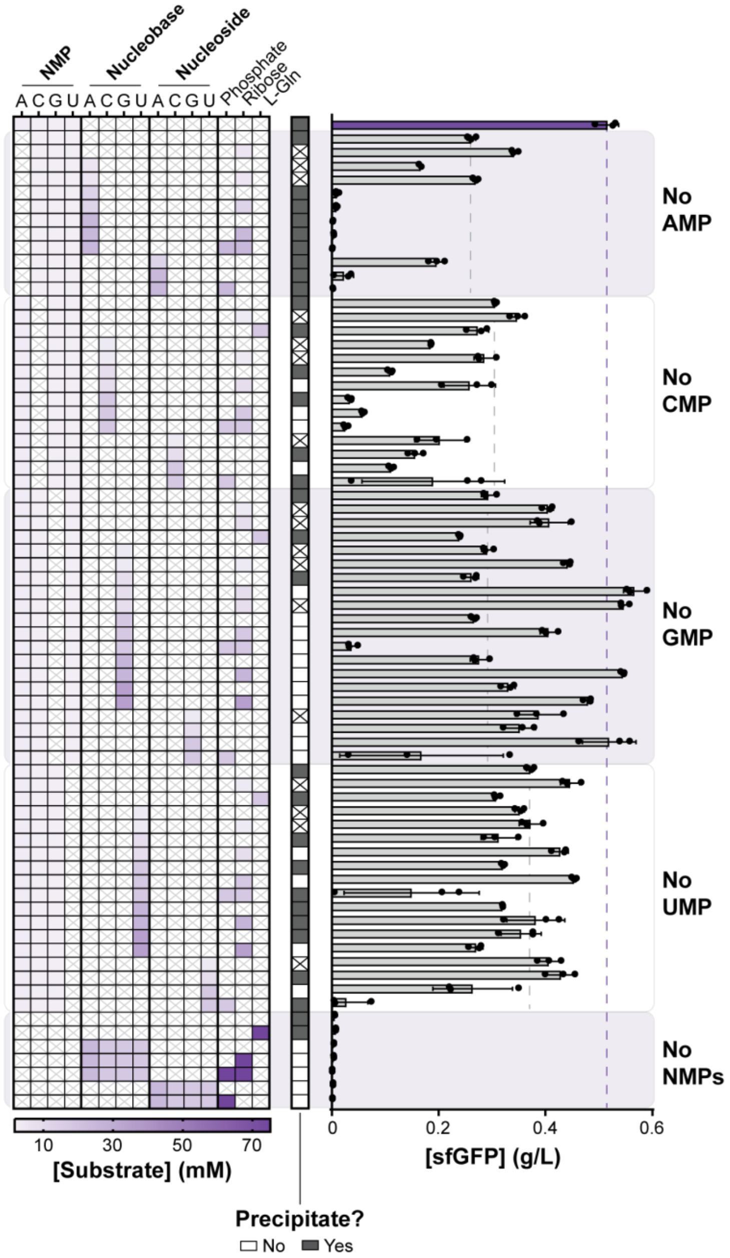

Supplementary Figure 5. Replacing NMPs with nucleobases or nucleosides. Using the minimal system as a reagent composition starting point, the listed NMP(s) were removed from the reagent formulation and replaced with either their corresponding nucleobase (adenine, cytosine, guanine, and uracil) or nucleoside (adenosine, guanosine, cytidine, and uracil). In some cases, phosphate and/or ribose were added to supplement the nucleotide synthesis pathway or attempt to improve overall system performance. L-Gln addition, a key component in nucleotide biosynthesis, was also assessed. In the reagent composition heat map, boxes with "X" indicate no addition of the reagent. Resultant sfGFP expression levels are shown. Dashed lines show the baseline expression levels for each condition; the purple bar is the unmodified minimal system. Data is presented as mean ± standard deviation of n = 3 replicates. All reactions were run using BL21 Star (DE3) lysate at 30 °C for 20 h. In many cases, insoluble precipitates were observed at the end of the reaction and are noted in the figure.

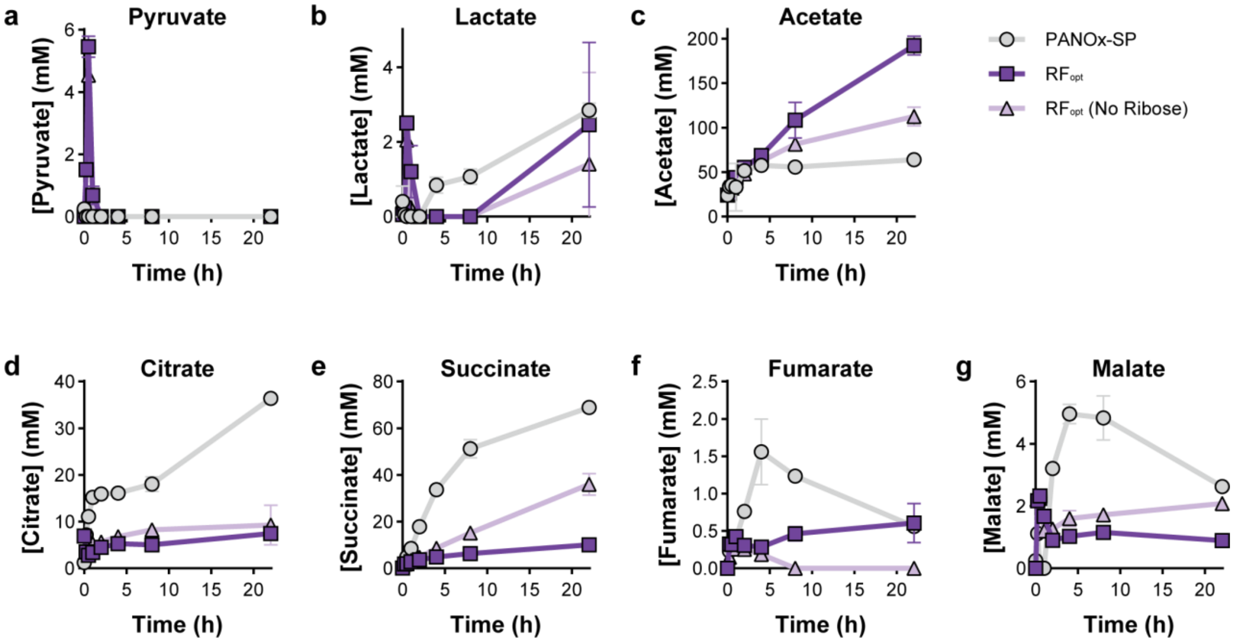

Supplementary Figure 6. Cell-free metabolite traces for different reagent formulations. Metabolic profiles of (a) pyruvate, (b) lactate, (c) acetate, (d) citrate, (e) succinate, (f) fumarate, and (g) malate over a 20-h batch reaction using either the PANOx-SP formulation, RF_{opt} formulation, or RF_{opt} formulation without addition of ribose. Reactions were run using the BL21 Star (DE3) lysate for 22 h at 30 °C. Data is presented as mean ± standard deviation of n = 3 replicates.

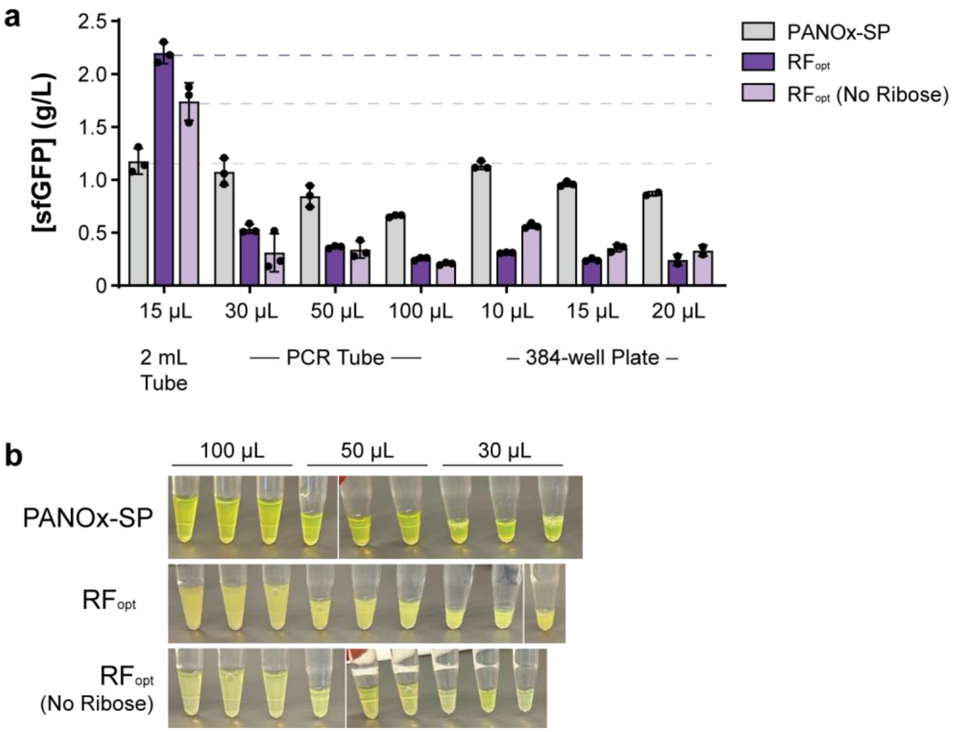

Supplementary Figure 7. Influence of reaction volume and geometry on protein expression. (a) Cell-free sfGFP expression levels from different volume reactions after 20-h incubation at 30 °C in either a 2-mL flat-bottomed tube (Axygen MCT-200-A), PCR tube (Thermo Scientific AB 2000), or a 384-well plate (Greiner Bio-One 781096) sealed with an optically clear seal (Bio-Rad MSB1001). Data is presented as mean ± standard deviation of n = 3 replicates. (b) Cell-free reaction opacity in PCR tubes after 20 h.

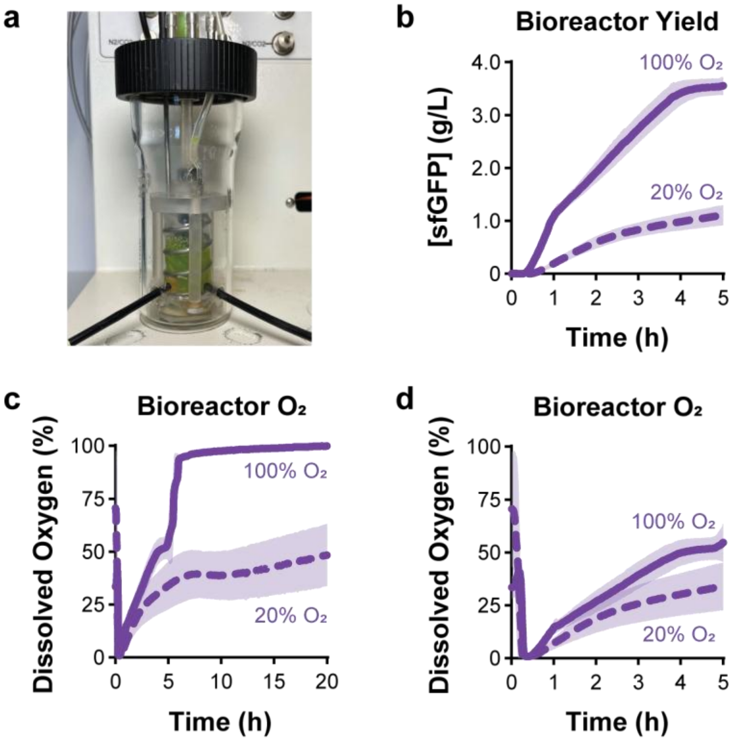

Supplementary Figure 8. (a) Photo of the in-house designed and constructed membrane-based bioreactor with a 2:1 aspect ratio (Sundberg 2025). (b) Zoomed-in version of the bioreactor sfGFP yield plot shown in Fig. 2e. (c) Dissolved oxygen content in the 4-mL bioreactor supplemented with either a 20% or 100% O_2 feed. (d) Zoomed-in version of the dissolved oxygen plot shown in (c). Data is presented as mean ± standard deviation of n = 6 bioreactors at 20% O_2 feed and n = 3 bioreactors at 100% O_2 feed.

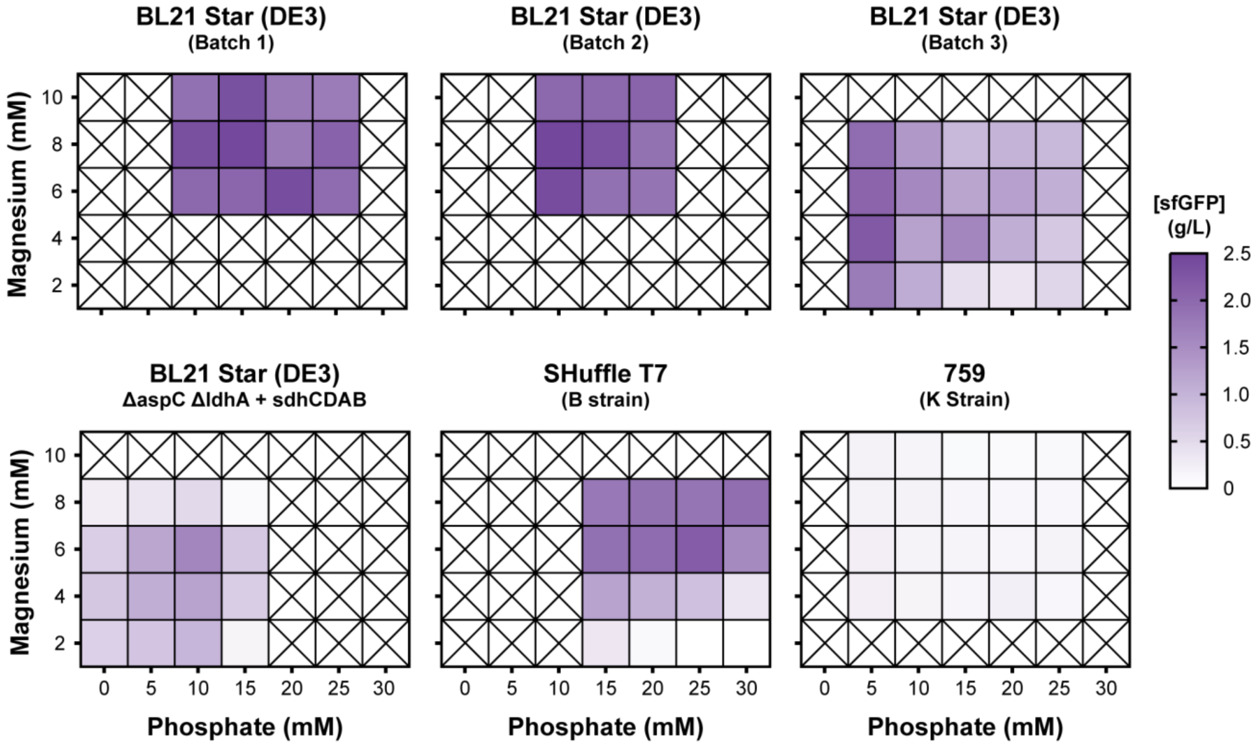

Supplementary Figure 9. Lysate-specific cell-free reaction optimization. Magnesium and phosphate cross-titrations and resultant sfGFP expression for six different lysates using RF_{opt}. Heat map cells represent the average of n = 3 replicates. All reactions were run at 30 °C for 20 h.

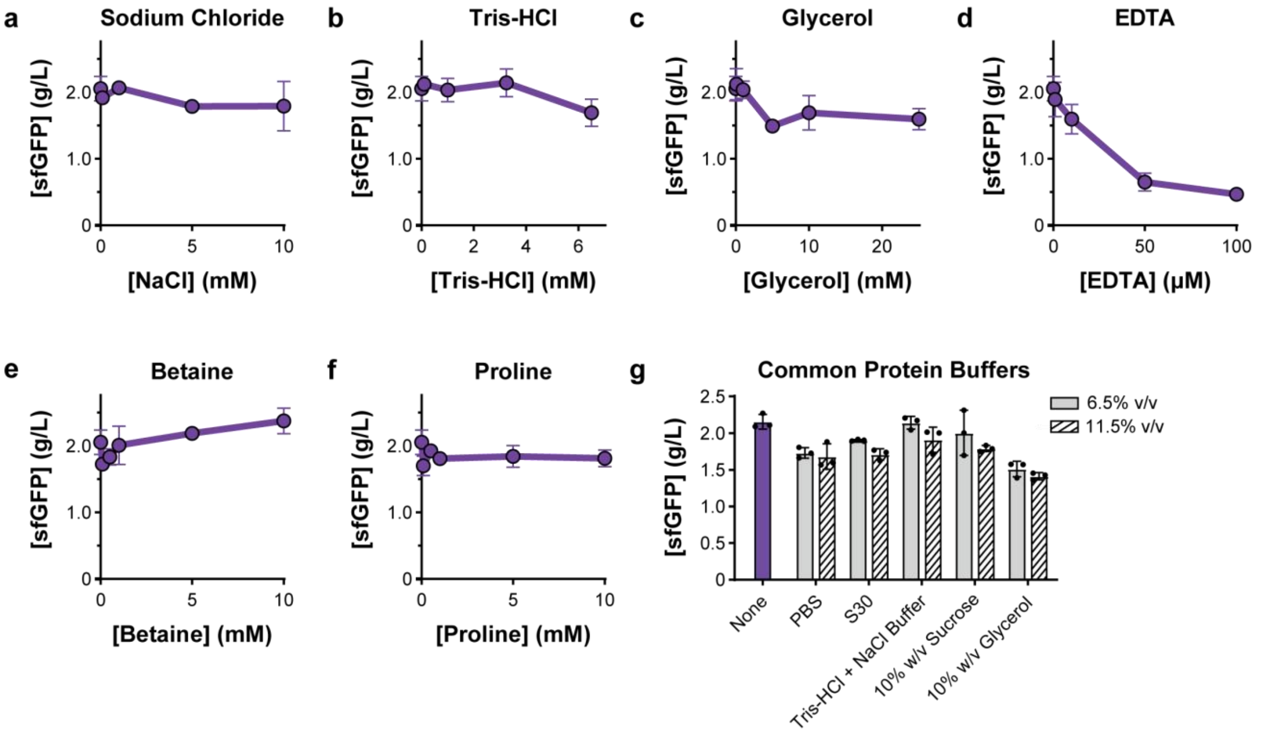

Supplementary Figure 10. RF_{opt} stability. sfGFP expression after addition of (a) sodium chloride, (b) Tris-HCl, (c) glycerol, (d) EDTA, (e) betaine, and (f) proline. (g) sfGFP expression when including either 6.5% or 11.5% v/v of common protein buffers and cryoprotectants in the cell-free reaction. PBS consisted of 137 mM NaCl, 2.7 mM KCl, 10 mM sodium phosphate dibasic, and 1.8 mM potassium phosphate dibasic. S30 buffer consisted of 10 mM Trizma acetate, 14 mM magnesium acetate, and 60 mM potassium acetate. Tris-HCl and NaCl buffer consisted of 100 mM Tris-HCl and 150 mM NaCl. In all panels, data is presented as mean ± standard deviation of n = 3 replicates. All reactions were run using BL21 Star (DE3) lysate and RF_{opt} with the listed additives at 30 °C for 20 h.

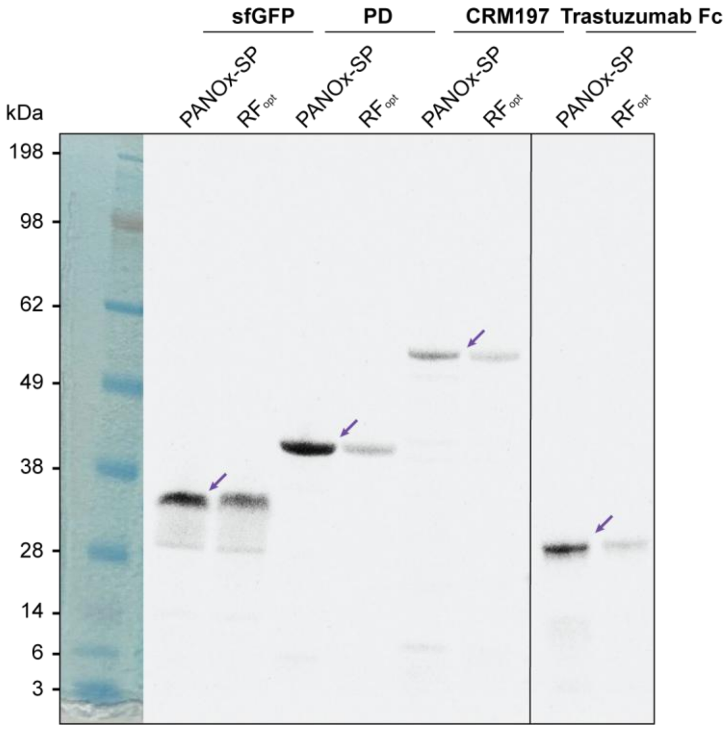

Supplementary Figure 11. Production of therapeutically relevant proteins in a reducing cell-free reaction environment. Autoradiograms of cell-free reactions expressing sfGFP, protein D (PD), CRM197, and the trastuzumab Fc domain with the RF_{opt} formulation. Protein bands were separated by SDS PAGE without addition of DTT or heat denaturation to observe presence of disulfide bonds, or lack thereof. Purple arrows indicate the correct protein molecular weight. Trastuzumab Fc should form a disulfide bonded dimer at 54.4 kDa. Note that although the molecular weight of sfGFP is 27.0 kDa, the protein runs at a higher molecular weight when not fully denatured. All reactions were run at 30 °C for 20 h using BL21 Star (DE3) lysate.

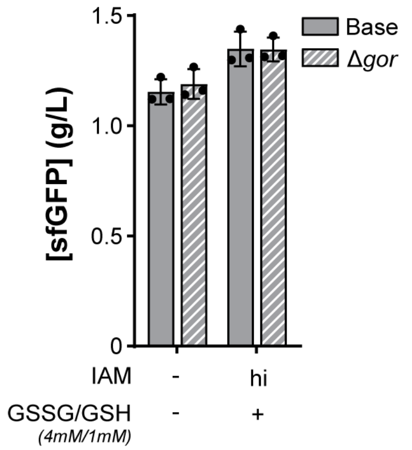

Supplementary Figure 12. PANOx-SP behavior in the presence of iodoacetamide (IAM) and glutathione (GSSG/GSH). Iodoacetamide was added at 500 μM (labeled hi). Reactions were run at 30 °C for 20 h. Corresponding RF_{opt} data is shown in Figure 4b. Data is presented as mean ± standard deviation of n = 3 replicates.

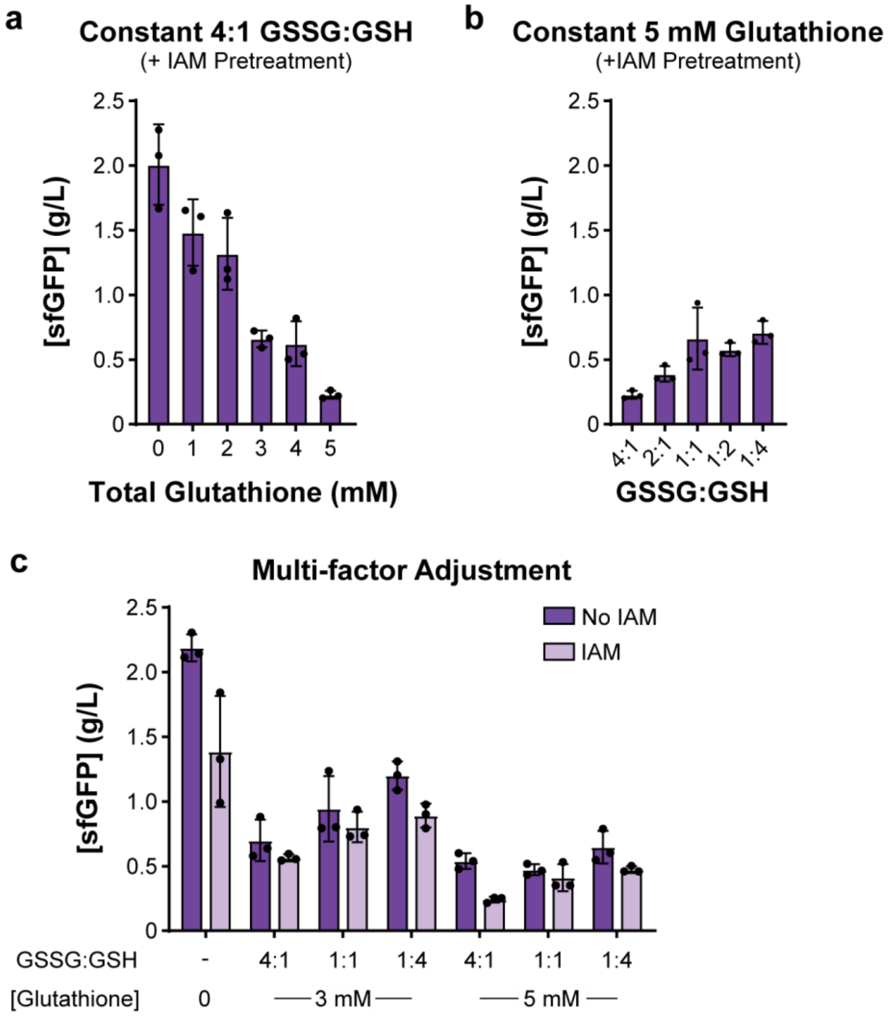

Supplementary Figure 13. Manipulation of RF_{opt} oxidizing environment. (a) sfGFP expression levels associated with including different concentrations of total glutathione, holding the 4:1 oxidized:reduced (GSSG:GSH) ratio constant. (b) sfGFP expression levels after altering the GSSG:GSH ratio, holding total glutathione concentrations constant at 5 mM. (c) Impact of iodoacetamide (IAM) pretreatment on cell-free sfGFP expression at different glutathione concentrations and GSSG:GSH ratios. Data in all panels is presented as mean ± standard deviation of n = 3 replicates. All reactions were run using BL21 Star (DE3) Δ*gor* lysate and RF_{opt} at 30 °C for 20 h.

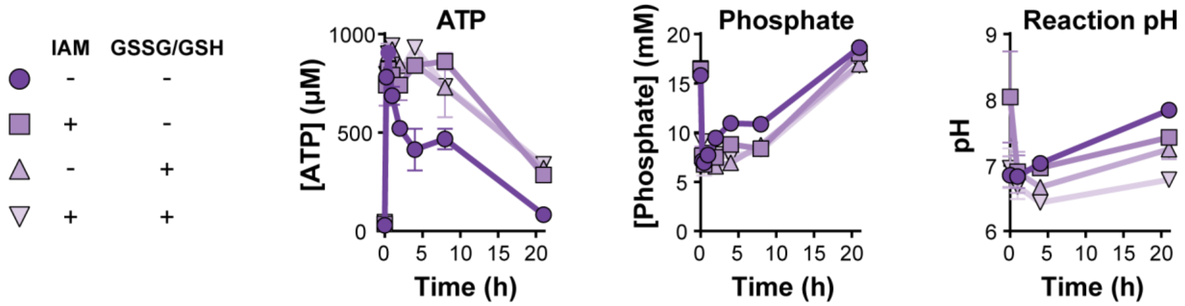

Supplementary Figure 14. Effect of iodoacetamide (IAM) and oxidized/reduced glutathione (GSSG/GSH) on ATP, inorganic phosphate, and reaction pH. All reactions were run using BL21 Star (DE3) Δ*gor* lysate and RF_{opt} at 30 °C for 21 h. Data is presented as mean ± standard deviation of n = 3 replicates.

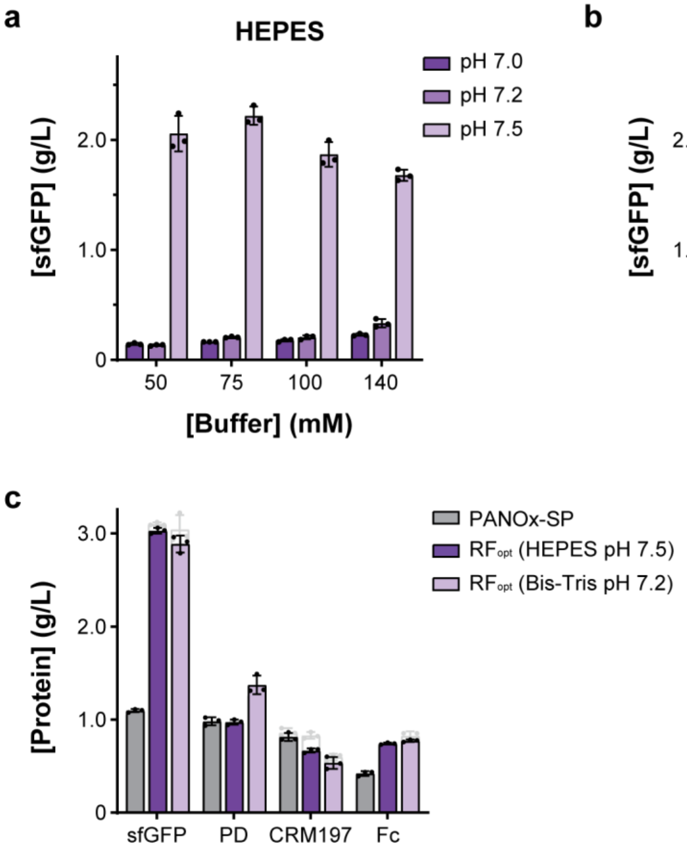

Supplementary Figure 15. Impact of buffer choice on oxidizing RF$_{opt}$ behavior. (a) sfGFP expression in the oxidizing RF$_{opt}$ system associated with the use of HEPES at different concentrations and initial pH levels. (b) sfGFP expression associated with the use of Bis-Tris at different concentrations and initial pH levels. (c) Expression of different protein products using the oxidizing version of either PANOx-SP, RF$_{opt}$ with HEPES pH 7.5 buffer, or RF$_{opt}$ with Bis-Tris pH 7.2 buffer. Data in all panels is presented as mean ± standard deviation of n = 3 replicates. All reactions were run using BL21 Star (DE3) Δ*gor* lysate at 30 °C for 20 h.

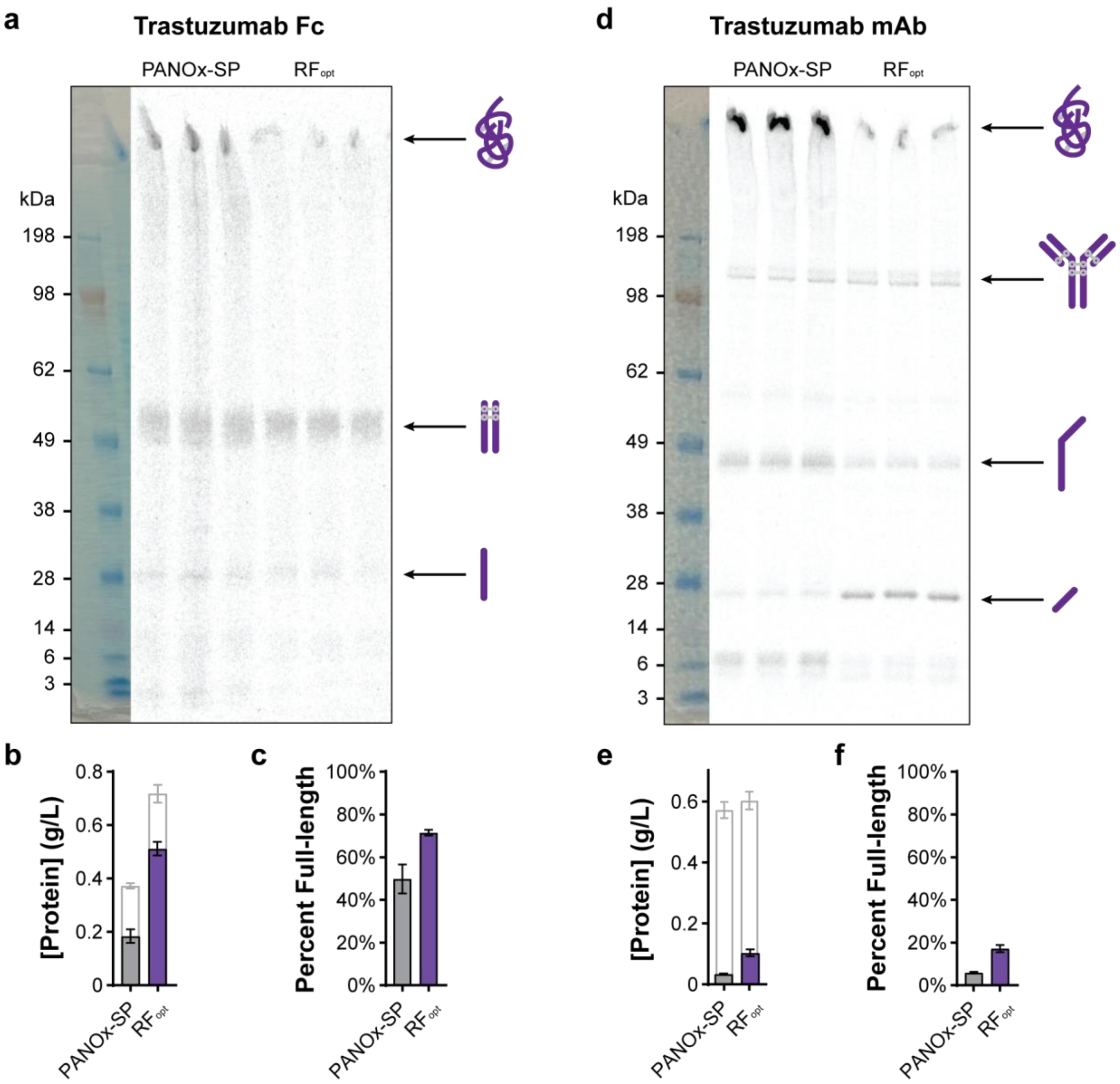

Supplementary Figure 16. Confirmation of disulfide bond formation using oxidizing RF$_{opt}$. (a) Autoradiogram of cell-free reactions expressing the trastuzumab Fc domain. Total samples were run directly on SDS-PAGE gels without treatment to preserve the disulfide bonds. (b) Total (gray bars) and soluble (colored bars) trastuzumab Fc cell-free expression yields. (c) Mass fraction of disulfide-bonded trastuzumab Fc dimers, as determined by densitometry. (d) Autoradiogram of cell-free reactions expressing trastuzumab heavy chain and light chain. Total samples were run directly on SDS-PAGE gels without treatment to preserve the disulfide bonds. (e) Total (gray bars) and soluble (colored bars) protein expression in reactions expressing trastuzumab heavy chain and light chain. (f) Percent full-length trastuzumab antibody, as determined by densitometry. Data is presented as mean ± standard deviation of n = 3 replicates. All reactions were run using BL21 Star (DE3) Δ*gor* lysate at 30 °C for 20 h in the oxidizing RF$_{opt}$ formulation.

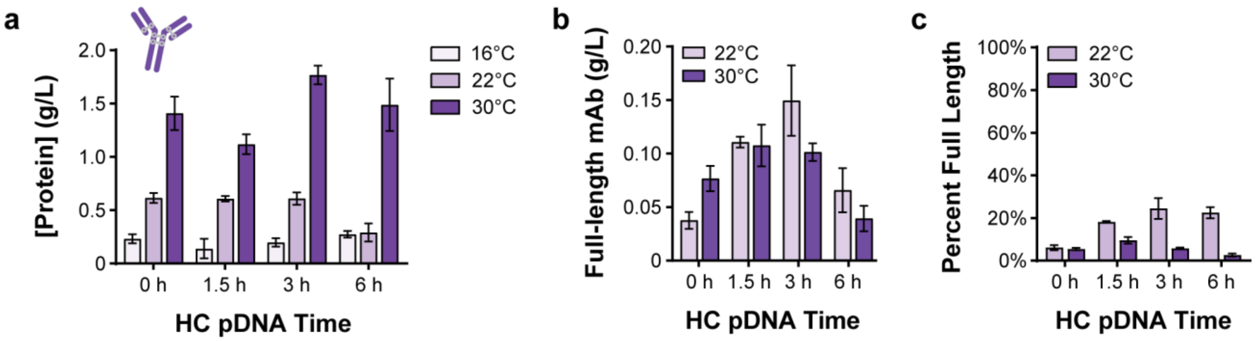

Supplementary Figure 17. Optimization of trastuzumab expression with oxidizing RF_{opt}. (a) Full amount of soluble protein expression from the trastuzumab heavy chain and light chain plasmids after 20 h. (b) Amount of full-length trastuzumab monoclonal antibody. Full-length antibody yields were determined by multiplying total protein yield by the fraction of full-length antibody (c). No results are reported for 16 °C reactions because bands were too faint to be distinguished from the background. (c) Fraction of full-length antibody observed for the expression of trastuzumab heavy chain and light chain proteins. Antibody yield data and error bars were calculated as described in Methods. All reactions were run using BL21 Star (DE3) Δ*gor* lysate and oxidizing RF_{opt}. All bars represent the average of n = 3 replicates.

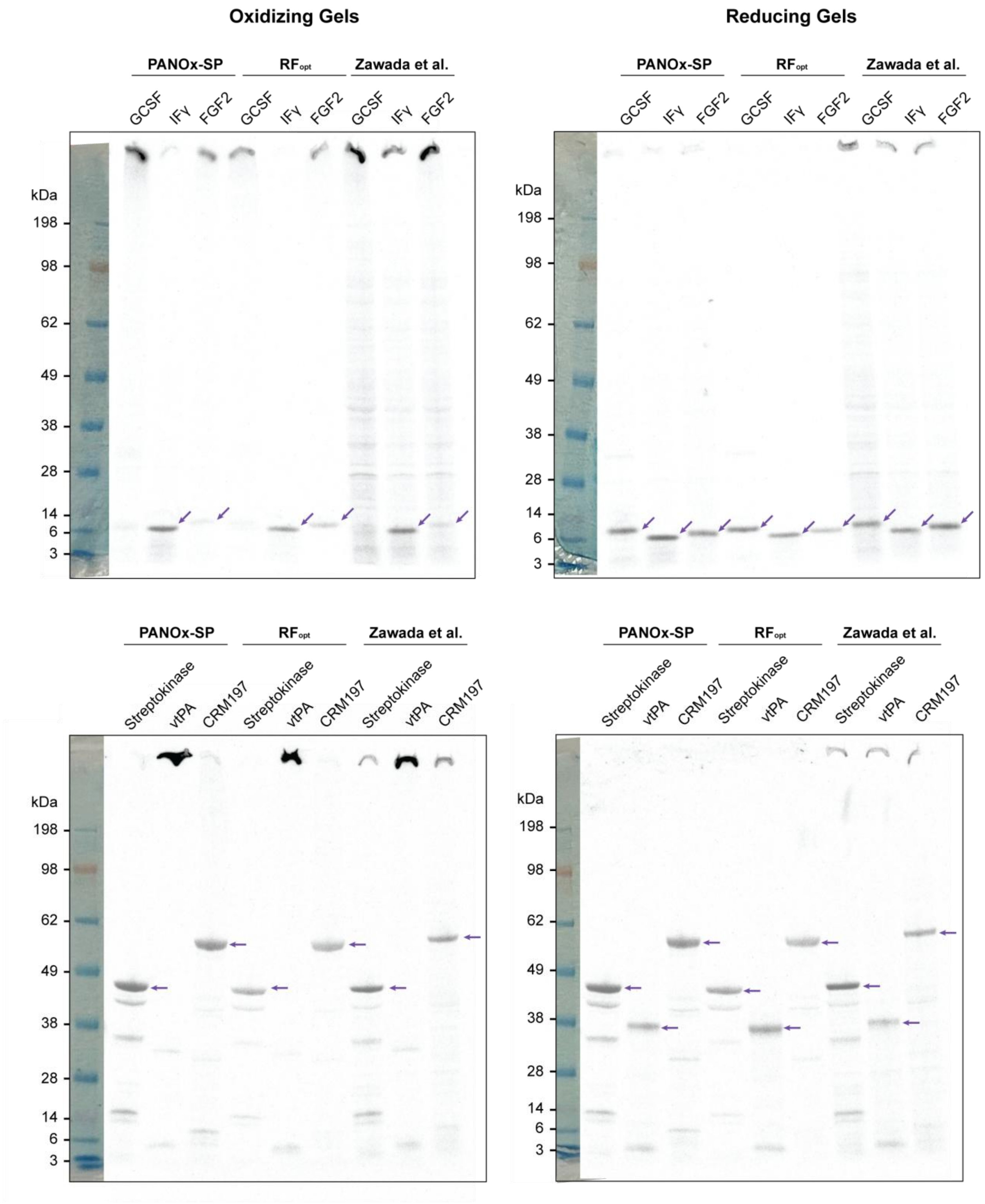

Supplementary Figure 18. Oxidizing and reducing autoradiograms for GCSF, IFγ, FGF2, streptokinase, vtPA, and CRM197. Correct protein band sizes are marked with a purple arrow.

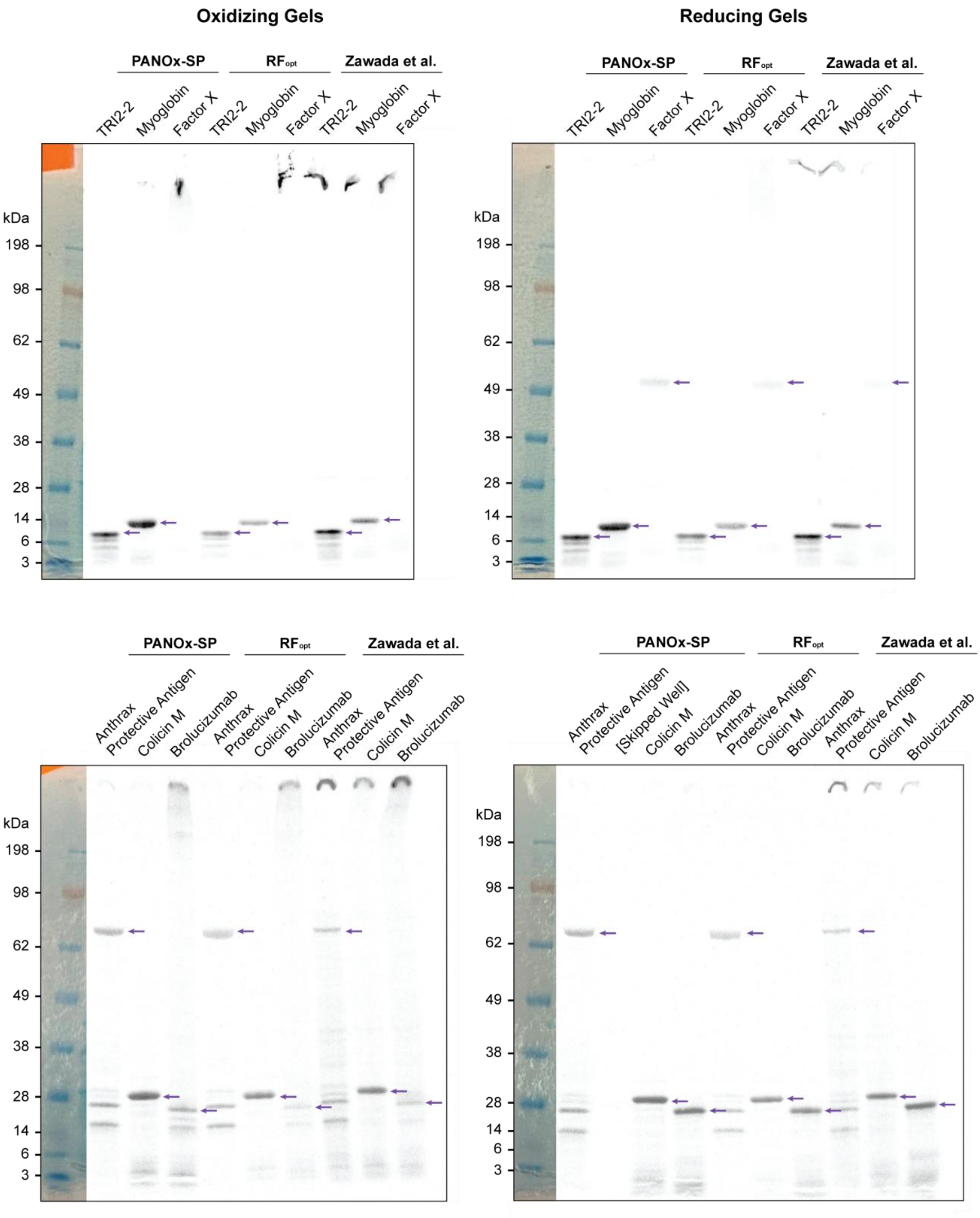

Supplementary Figure 19. Oxidizing and reducing autoradiograms for TRI2-2, myoglobin, Factor X, anthrax protective antigen, colicin M, and brolucizumab. Correct protein band sizes are marked with a purple arrow.

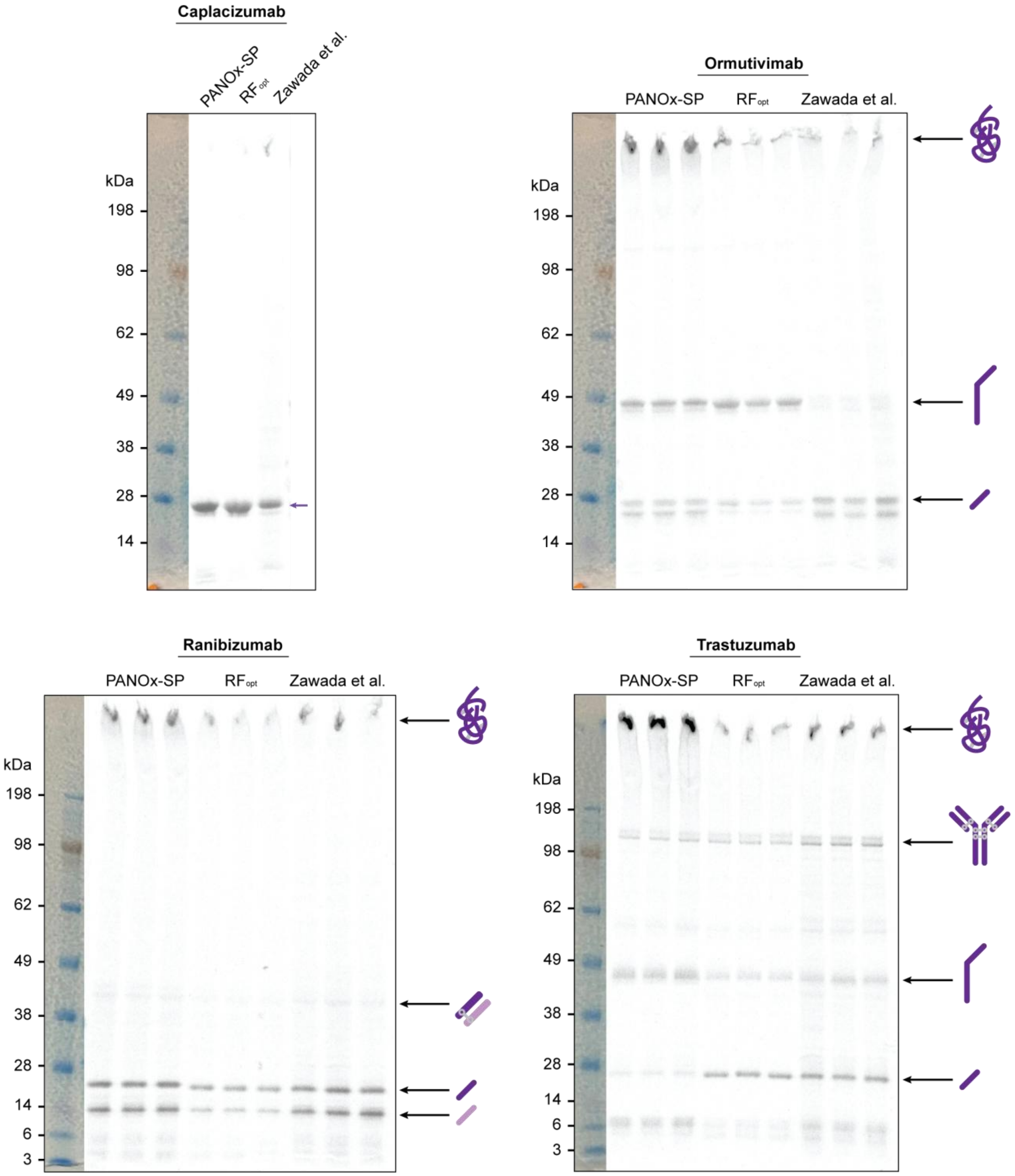

Supplementary Figure 20. Oxidizing autoradiograms for caplacizumab, ormutivimab, ranibizumab, and trastuzumab. Total fractions of cell-free expression reactions were run without treatment on SDS-PAGE gels to visualize disulfide-bonded components. Caplacizumab and ormutivimab were run on the same gel and separated in the above images for easier labeling. The trastuzumab gel was shown previously in Supplementary Fig. 16 and is repeated here with the Zawada et al. (2011) samples for easier comparison. Correct protein band sizes are marked with arrows.

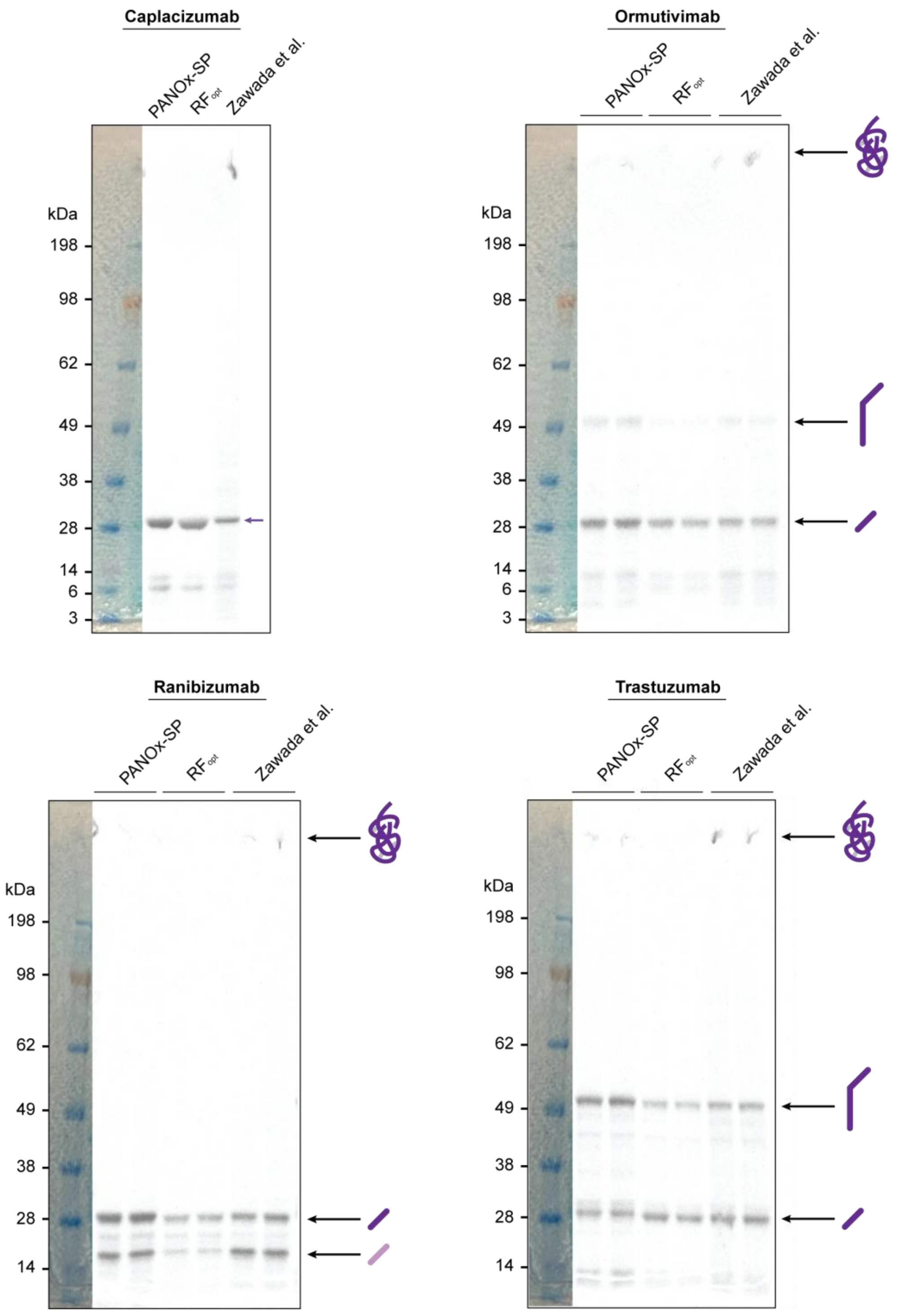

Supplementary Figure 21. Reducing autoradiograms for caplacizumab, ormutivimab, ranibizumab, and trastuzumab. Total cell-free reagent samples were treated with DTT and heated at 70 °C for 3 min. to denature the samples and break disulfide bonds between components before running the SDS-PAGE gels. Caplacizumab and ormutivimab samples were run on the same gel and separated in the above images for easier labeling of bands; ranibizumab and trastuzumab gels were treated similarly. Correct protein band sizes are marked with arrows.

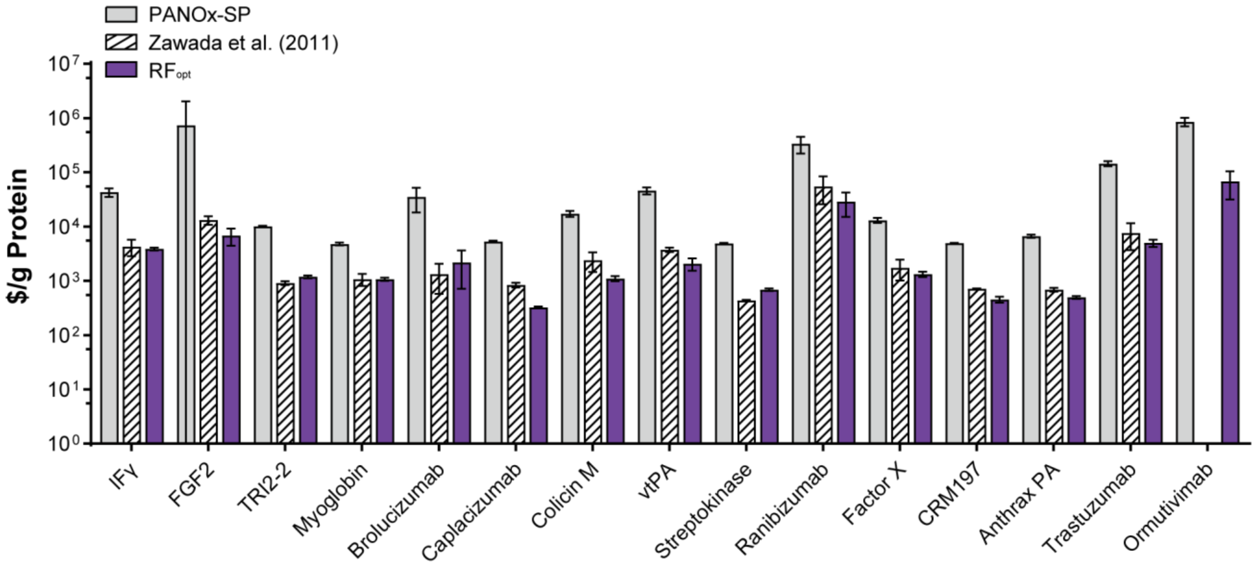

Supplementary Figure 22. Therapeutic protein reagent cost per gram product. Cost was determined from protein yield (Fig. 5a) and cost of reagents per liter for each system (Table S2). Costs associated with the PANOx-SP and RF_{opt} are increased to $4,898/L and $518/L in these calculations to account for the addition of oxidized and reduced glutathione; the Zawada et al. (2011) formulation already included glutathione. Cost of the added bacterial DsbC is not included. Costs were calculated for soluble protein produced. For ranibizumab, trastuzumab, and ormutivimab, this was the amount of full-length, soluble protein assemblies as determined by densitometry. Data is presented as calculated $\$/g_{protein}$ ± propagated error.

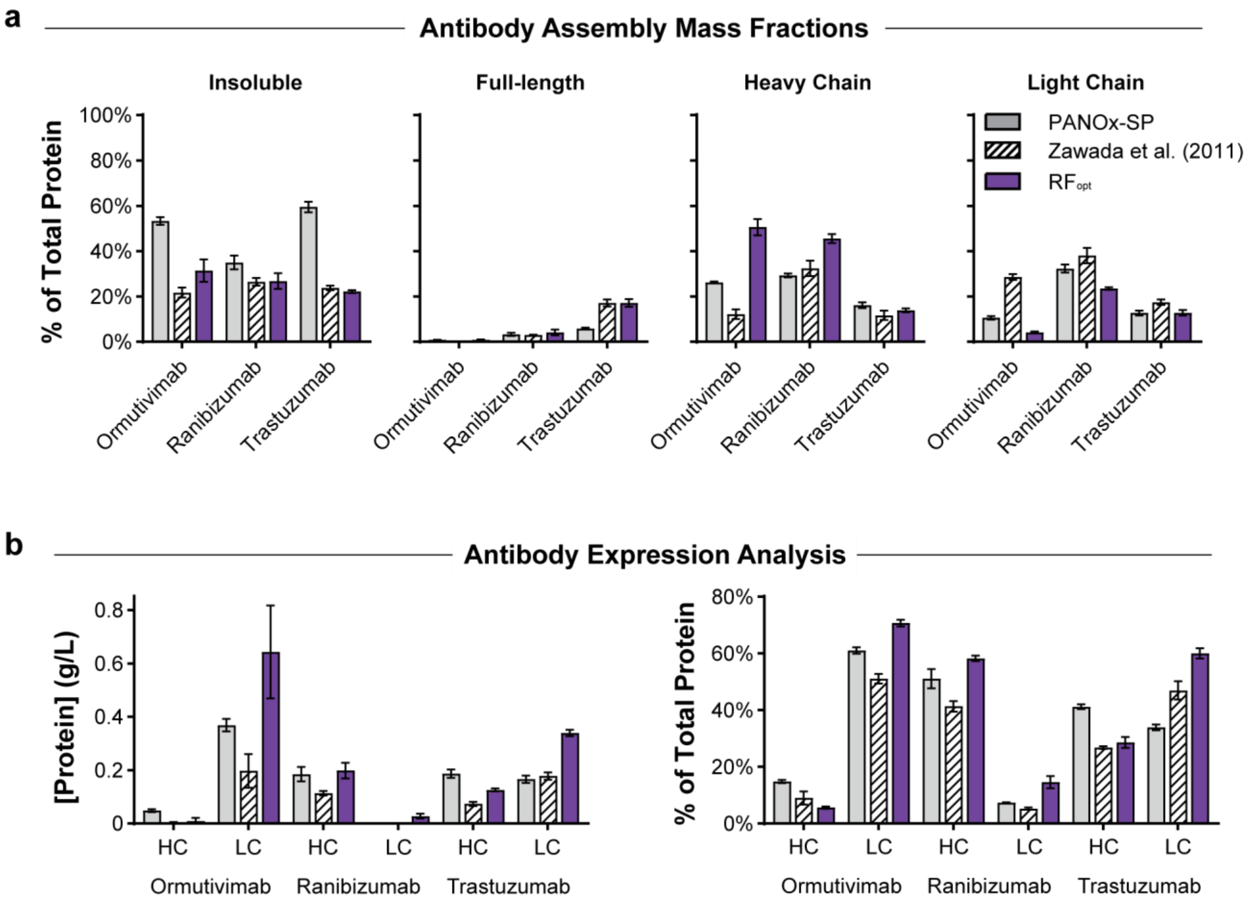

Supplementary Figure 23. Antibody component expression and assembly. (a) Mass fractions of the insoluble, full-length, heavy chain, and light chain components for three antibody constructs. Fractions were determined through densitometry of the oxidizing autoradiograms in Supplementary Fig. 19. Data is presented as mean ± standard deviation of n = 3 replicates. (b) Concentration and mass fraction of the heavy and light chain components for three antibody constructs, as determined through densitometry of the reducing autoradiograms in Supplementary Fig. 20. Mass fraction error bars represent the standard deviation of n = 2 replicates, and protein concentration error bars represent propagated error from both the n = 3 yield quantification replicates and the n = 2 autoradiogram replicates.

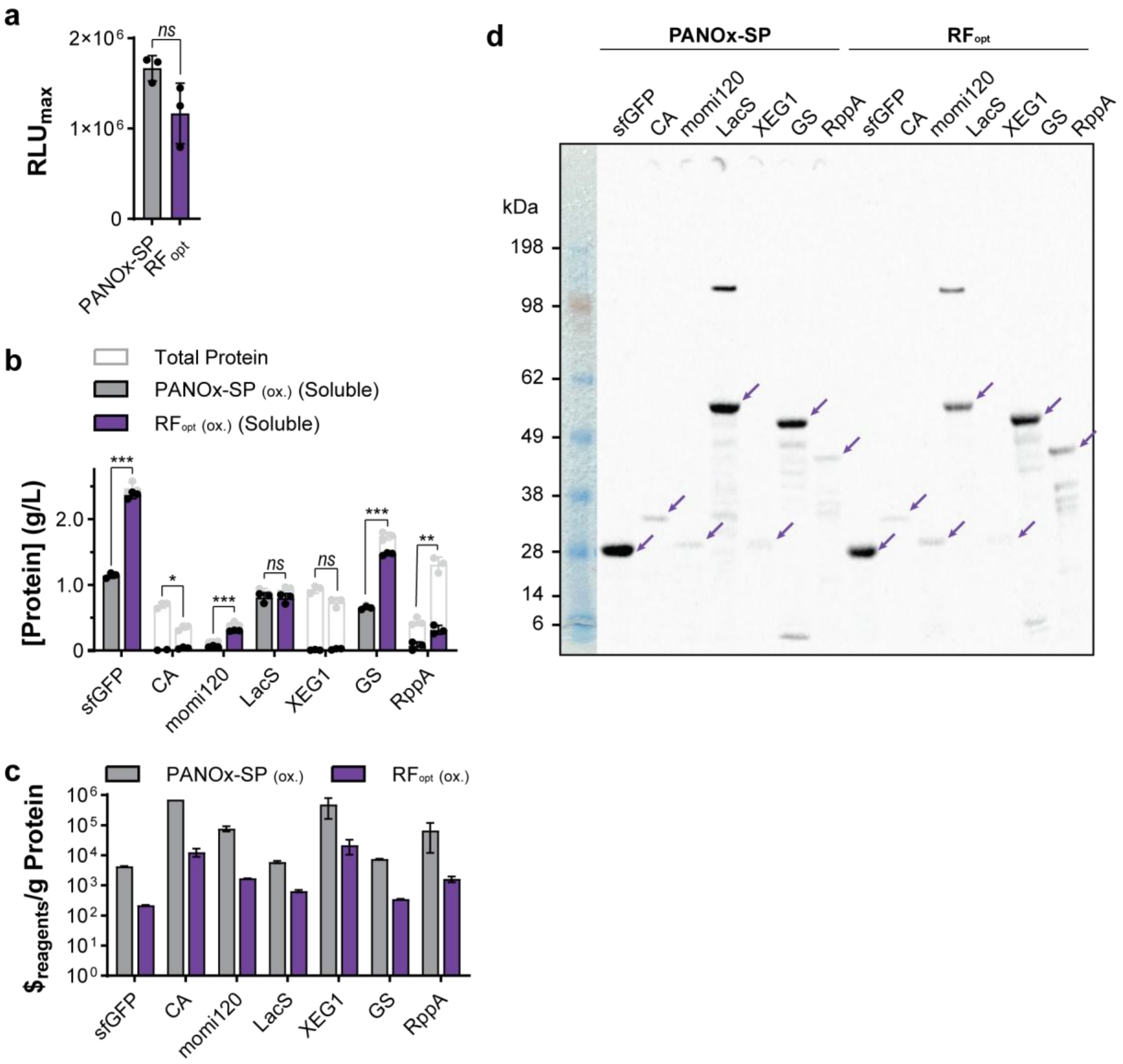

Supplementary Figure 24. Additional non-therapeutic protein expression in RF$_{opt}$.
(a) Maximum luminescence activity achieved by luciferase expressed in PANOx-SP or RF$_{opt}$ (p = 0.00003). The average maximum activity ± standard deviation of n = 3 replicates is shown. (b) Expression of sfGFP, carbonic anhydrase (CA), momi120, β-galactosidase (LacS), xyloglucan-specific endo-beta-1,4-glucanase (XEG1), glutamine synthase (GS), and type III polyketide synthase RppA. The average of both total and soluble protein yields ± standard deviation are shown for n = 3 replicates. For (a) and (b), reactions were run in an oxidizing cell-free environment with BL21 Star (DE3) lysate and included 5 μM of purified bacterial DsbC. Reactions were incubated at 30 °C for 20 h. Statistical significance for soluble yields was calculated by unpaired two-tailed *t*-tests (in order, p = 0.000006, 0.02, 0.000007, 0.9, 0.2, 0.000001, 0.009). (c) Reagent cost per gram product of proteins expressed in (b) following the same procedures addressed in Supplementary Fig. 22. (d) Reducing autoradiogram for the proteins expressed in (d). Soluble protein fractions were treated with DTT and heated at 95 °C for 10 min. before running the SDS-PAGE gel. Correct band sizes are marked with purple arrows.

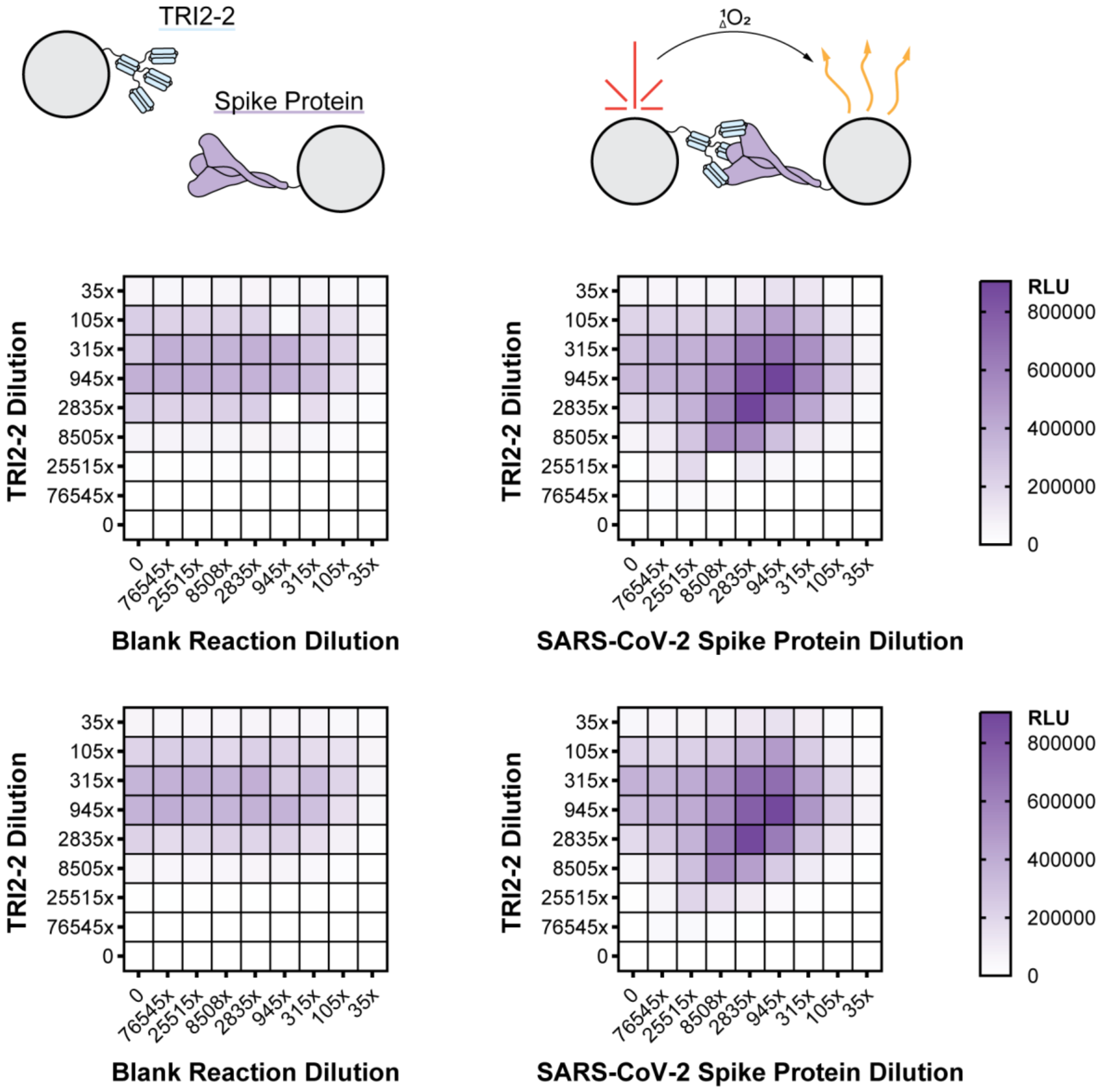

Supplementary Figure 25. TRI2-2 binding activity. Heatmaps demonstrating AlphaLISA luminescence associated with binding between the TRI2-2 minibinder and the SARS-CoV-2 RBD spike protein. Duplicate reactions with either a blank cell-free reaction (no DNA) or a cell-free reaction expressing TRI2-2 are shown.

Supplementary Tables

Supplementary Table 1. $/L$_{CFE}$ calculations for cell-free expression reactions including lysate, plasmid DNA, and one of the listed reagent formulations. Lysate and plasmid DNA values were previously calculated in Warfel et al. (2023).

**$/L$_{CFE}$**	**PANOx-SP**	**Minimal**	**RF$_{opt}$**
Small molecule reagents	$4,535	$93	$143
Lysate	$1,200	$1,200	$1,200
Plasmid DNA	$333	$333	$333
Total	$6,068	$1,626	$1,676
**% Reagent Cost**	**74.7%**	**5.7%**	**8.5%**

Supplementary Table 2. Reagents used in this work. Reagent prices were determined by the cost of the largest available retail unit offered by vendors on 4 April 2024. The Roche tRNA price was recorded prior to the vendor stopping product production. No *E. coli* tRNA is currently offered by any vendor as of 4 April 2024.

Reagent Name	Abbreviation	Vendor	Catalog Number	Preparation Notes*	$/g reagent	$/L/mM**
L-glutamic acid hemimagnesium salt	$Mg(Glu)_2$	Sigma	49605	-	$0.32	$0.13
L-glutamic acid ammonium salt	$NH_4(Glu)$	Biosynth	FG28929	-	$4.39	$0.72
L-glutamic acid potassium salt	K(Glu)	Sigma	G1501	-	$0.40	$0.08
D-(+)-Glucose	Glucose	Sigma	G8270	-	$0.01	$0.002
Potassium phosphate dibasic	Potassium Phosphate	Sigma	60353	1.6:1 ratio of dibasic to monobasic potassium phosphate. Adjusted to pH 7.	$0.39	$0.07
Potassium phosphate monobasic		Sigma	P0662		$0.11	$0.02
Nicotinamide	-	Sigma	72340	-	$0.18	$0.02
D-(-)-Ribose	Ribose	Sigma	R7500	-	$1.83	$0.27
HEPES	-	Sigma	H3375	Adjusted to pH 7.2.	$0.56	$0.13
Bis-Tris	-	Sigma	B9754	Adjusted to pH 7.2.	$1.02	$0.21
Sodium pyruvate	Pyruvate	Sigma	P5280	-	$1.66	$0.18
Putrescine dihydrochloride	Putrescine	Sigma	P5780	-	$5.40	$0.87
Spermidine	-	Sigma	S2626	-	$28.80	$4.18
Dithiothreitol	-	Sigma	43816	-	$2.04	$0.31
Folinic acid calcium salt hydrate	Folinic Acid	Sigma	47612	-	$897.75	-
tRNA, from E. coli	tRNA	Roche	10109541001	-	$1,919.00	-
Coenzyme A sodium salt hydrate	CoA	Sigma	C3144	-	$2,710.00	$2,080.00
Nicotinamide adenine dinucleotide	NAD	Sigma	N8535	-	$502.67	$333.48

Supplementary Table 2 (continued).

Reagent Name	Abbreviation	Vendor	Catalog Number	Preparation Notes*	$/g reagent	$/L/mM**
Adenosine 3',5'-cyclic monophosphate	cAMP	Sigma	A9501	Adjusted to pH ~8.	$113.60	$37.40
Phosphoenolpyruvate monopotassium salt	PEP	Sigma	10108294001	Adjusted to pH 7.	$373.00	$76.89
D-(-)-3-Phosphoglyceric acid disodium	3-PGA	Sigma	P8877	Adjusted to pH ~7.5.	$336.00	$77.29
Potassium oxalate monohydrate	Oxalic Acid	Sigma	P0963	-	$0.32	$0.06
L-glutathione oxidized	GSSG	Sigma	G4501	-	$157.20	$96.31
L-glutathione reduced	GSH	Sigma	G4521	-	$5.04	$1.55
Iodoacetamide	IAM	Sigma	I1149	-	$11.60	$2.15
Maltodextrin	-	Sigma	419672	Prepared fresh for each use.	$0.39	-
Poly(ethylene glycol)	PEG-8000	Sigma	P5413	-	$0.12	-
Ammonium acetate	-	Sigma	A1542	-	$0.13	$0.01
Adenosine 5'-triphosphate	ATP	Sigma	A2383	Adjusted to pH 7-7.2.	$27.92	$15.39
Cytidine 5'-triphosphate	CTP	Sigma	C1506	Adjusted to pH 7-7.2.	$530.00	$279.37
Guanosine 5'-triphosphate	GTP	Sigma	G8877	Adjusted to pH 7-7.2.	$752.00	$393.43
Uridine 5'-triphosphate	UTP	Sigma	U6625	Adjusted to pH 7-7.2.	$688.00	$378.46
Adenosine 5'-monophosphate	AMP	Sigma	01930	Adjusted to pH 7-7.2.	$17.88	$6.99
Cytidine 5'-monophosphate	CMP	Sigma	C1006	Adjusted to pH 7-7.2.	$34.60	$12.70
Guanosine 5'-monophosphate	GMP	Sigma	G8377	Adjusted to pH 7-7.2.	$13.10	$5.33
Uridine 5'-monophosphate	UMP	Sigma	U6375	Adjusted to pH 7-7.2.	$22.80	$8.39

Supplementary Table 2 (continued).

Reagent Name	Abbreviation	Vendor	Catalog Number	Preparation Notes*	$/g reagent	$/L/mM**
L-Valine	Amino Acids	Sigma	V0500	Amino acids added in the listed order to a single mixture.	$0.64	$0.07
L-Tryptophan		Sigma	T0254		$0.87	$0.18
L-Phenylalanine		Sigma	P2126		$0.65	$0.11
L-Isoleucine		Sigma	I2752		$1.21	$0.16
L-Leucine		Sigma	L8000		$0.72	$0.09
L-Cysteine		Sigma	C7352		$0.95	$0.12
L-Methionine		Sigma	M9625		$0.42	$0.06
DL-Alanine		Sigma	A7627		$0.71	$0.06
L-Arginine		Sigma	A8094		$0.46	$0.08
L-Asparagine		Sigma	A0884		$0.97	$0.13
L-Aspartic acid		Sigma	A9256		$0.72	$0.10
L-Glutamic acid potassium salt		Sigma	G1501		$0.40	$0.08
L-Glycine		Sigma	G7126		$0.06	$0.005
L-Glutamine		Sigma	G3126		$0.69	$0.10
L-Histidine		Sigma	H8000		$0.62	$0.10
L-Lysine		Sigma	L5501		$0.43	$0.08
L-Proline		Sigma	P0380		$0.72	$0.08
L-Serine		Sigma	S4500		$0.85	$0.09
L-Threonine		Sigma	T8625		$1.35	$0.16
L-Tyrosine		Sigma	T3754		$1.16	$0.21
Pantothenic acid	-	Sigma	21210	-	$0.60	$0.14
Nicotinic acid	-	Sigma	N4126	-	$0.11	$0.01
Sodium fumarate	-	Sigma	F1506	-	$0.28	$0.04
Malic acid	-	Sigma	M6413	-	$2.89	$0.39
Adenine	-	Sigma	A8626	Adjusted to pH 7-7.2.	$2.81	$0.38
Adenosine	-	Sigma	A9251	Adjusted to pH 7-7.2.	$0.30	$0.08
Cytidine	-	Sigma	C122106	Adjusted to pH 7-7.2.	$7.63	$1.86
Cytosine	-	Sigma	C3506	Adjusted to pH 7-7.2.	$12.20	$1.36
Guanine	-	Sigma	G11950	Adjusted to pH 7-7.2.	$0.89	$0.13

Supplementary Table 2 (continued).

Reagent Name	Abbreviation	Vendor	Catalog Number	Preparation Notes*	$/g reagent	$/L/mM**
Guanosine	-	Sigma	G6752	Adjusted to pH 7-7.2.	$1.72	$0.49
Uridine	-	Sigma	U3750	Adjusted to pH 7-7.2.	$0.44	$0.11
Uracil	-	Sigma	U0750	Adjusted to pH 7-7.2.	$0.63	$0.07
Starch, soluble	-	Sigma	S9765	Prepared fresh for each use.	$0.15	-
Sodium chloride	-	Sigma	S3014	-	$0.04	$0.003
Trizma hydrochloride	Tris-HCl	Sigma	T5941	-	$0.25	$0.04
Glycerol	-	Sigma	G5516	-	$0.10	$0.01
EDTA	-	Invitrogen	AM9262	-	$1.11	$0.32
Betaine	-	Sigma	61962	-	$1.07	$0.13
Sucrose	-	Sigma	S0389	-	$0.05	$0.02

*Unless otherwise noted, all reagents were dissolved in MilliQ water, flash frozen in liquid nitrogen, and stored at -80°C. All pH adjustments were made with either potassium hydroxide (Sigma P5958) or acetic acid (Sigma A6283).

**Reagents with no listed values in this column are typically added on a mass basis, not a molar basis.

Supplementary Table 3. *E. coli* strains used in this work.

Strain Name	Source	Genotype	Usage
BL21 Star (DE3)	Invitrogen C601003	F⁻*ompT hsdSB* (r_B–, m_B–) *gal dcm rne*131 (DE3)	Cell-free extract
BL21 Star (DE3) Δ*gor*	This Work	F⁻*ompT hsdSB* (r_B–, m_B–) *gal dcm rne*131 (DE3) *Δgor*	Cell-free extract
Shuffle T7 Express	NEB C3029J	*fhuA2 lacZ::T7 gene1 [lon] ompT ahpC gal λatt::pNEB3-r1-cDsbC (SpecR, lacIq) ΔtrxB sulA11 R(mcr-73::miniTn10--TetS)2 [dcm] R(zgb-210::Tn10 --TetS) endA1 Δgor Δ(mcrC-mrr)114::IS10*	Cell-free extract
759	Des Soye, et al. (2019). *Cell Chem. Bio.*	C321.ΔA. -*ompT* endA⁻ gor⁻ rne⁻ mazF⁻ T7	Cell-free extract
CLM24	Stark, et al. (2021). *Science Advances.*	W3110 ΔwaaL	Indicator cells
DH5α	NEB C2987H	*fhuA2Δ(argF-lacZ)U169 phoA glnV44 Φ80Δ(lacZ)M15 gyrA96 recA1 relA1 endA1 thi-1 hsdR17*	Plasmid propagation

Supplementary Table 4. Protein sequences used in this work. All sequences were codon optimized for expression in *E. coli* and cloned into the pJL1 backbone (Addgene 69496) by Twist Bioscience at the NdeI/SalI restriction enzyme sites.

Protein Product	Protein Sequence Source	Protein Sequence
Anthrax Protective Antigen	UniProt P13423	ATGGAAGTTAAACAGGAGAATCGCTTATTGAACGAGTCGGAATCTTCTTCACAAGGCCTGTTG<br>GGATATTACTTTTCAGACTTGAACTTCCAAGCACCTATGGTTGTCACGTCGTCAACCACAGGA<br>GACTTGTCCATCCCGTCTTCCGAGCTCGAGAATATCCCGTCTGAAAATCAATACTTTCAGTCA<br>GCTATATGGTCAGGATTTATAAAGGTTAAGAAAAGTGACGAGTATACATTCGCGACGTCTGCAG<br>ACAATCATGTTACGATGTGGGTAGACGATCAAGAAGTAATTAATAAGGCCTCTAACTCAAATAA<br>GATTAGACTGGAGAAGGGACGCCTTTACCAGATCAAGATTCAGTACCAAAGAGAAAACCCGA<br>CTGAGAAAGGATTAGACTTCAAGTTATACTGGACTGACTCCCAGAACAAGAAGGAAGTGATCA<br>GCAGCGACAACTTGCAACTCCCTGAATTAAAGCAAAAGAGTAGCAATTCGCGAAAGAAGCGT<br>AGTACATCTGCGGGGCCGACCGTTCCGGATCGCGATAATGACGGTATACCGGACAGCTTAGA<br>GGTCGAAGGGTACACTGTTGATGTTAAGAACAAGCGGACTTTCTTGTCTCCATGGATAAGCAA<br>TATACACGAAAAGAAGGGCCTGACTAAATACAAGTCAAGCCCCGAGAAGTGGTCCACAGCAT<br>CCGATCCCTACTCAGATTTCGAGAAGGTTACCGGCAGAATTGACAAGAATGTGAGTCCAGAG<br>GCTCGTCATCCTTTAGTTGCTGCATACCCAATCGTCCATGTAGATATGGAGAACATCATACTGA<br>GCAAGAACGAGGACCAGAGTACGCAGAACACGGACTCGCAGACGCGGACGATCTCAAAGAA<br>CACCAGTACTAGTCGCACTCACACGTCTGAGGTACATGGTAATGCGGAAGTTCACGCCTCGT<br>TCTTCGATATCGGCGGTTCTGTTAGTGCGGGTTTCTCAAACTCTAATTCAAGTACCGTAGCGAT<br>CGATCACTCACTGTCGCTTGCTGGTGAAAGAACCTGGGCAGAGACAATGGGTCTTAATACGG<br>CTGACACAGCCCGCCTCAATGCGAACATCCGTTACGTCAATACGGGAACAGCCCCCATCTAC<br>AATGTCCTTCCAACAACTAGCCTCGTGCTGGGGAAGAATCAGACCTTGGCAACTATCAAGGC<br>TAAGGAAAATCAGCTGTCGCAGATCTTGGCACCAAATAACTACTACCCAAGTAAGAACCTTGC<br>GCCGATCGCCTTGAACGCTCAAGACGACTTCAGCTCCACACCTATCACCATGAACTACAACC<br>AGTTTCTGGAACTCGAGAAAACCAAGCAACTCCGATTGGACACGGACCAAGTTTACGGGAAC<br>ATCGCGACATATAACTTCGAGAACGGACGGGTCCGGGTAGACACAGGTTCCAATTGGAGCGA<br>AGTCTTGCCCCAAATTCAGGAGACCACTGCGCGCATTATATTCAACGGGAAGGACCTGAACT<br>TAGTGGAGCGACGTATCGCGGCTGTAAATCCGTCGGACCCGCTTGAAACCACCAAGCCGGA<br>CATGACCCTTAAGGAAGCCTTAAAGATCGCGTTCGGTTTTAACGAGCCCAACGGCAACCTTC<br>AGTACCAGGGCAAGGACATAACAGAGTTCGACTTCAACTTCGATCAACAAACCTCTCAAAATA<br>TTAAGAATCAGCTGGCCGAACTGAACGCAACTAACATATACACTGTGCTTGATAAGATCAAGCT<br>GAACGCCAAAATGAACATTCTGATCCGAGATAAGCGGTTCCATTATGATCGAAACAATATTGCT<br>GTTGGAGCAGACGAGAGTGTTGTGAAAGAGGCCCACCGTGAGGTAATCAATAGTTCGACTGA<br>GGGCTTATTACTGAACATAGACAAGGACATTCGCAAGATCTTAAGCGGTTACATCGTCGAAATT<br>GAAGACACGGAAGGCCTCAAGGAAGTGATTAATGACCGATATGACATGTTGAACATCAGTAGT<br>TTGCGACAGGACGGCAAGACGTTCATAGACTTCAAGAAGTACAATGACAAACTCCCTTTATAC<br>ATTTCCAATCCGAATTATAAGGTCAACGTTTACGCGGTTACGAAGGAGAACACCATTATCAACC<br>CTTCTGAGAACGGCGACACCAGCACTAACGGGATCAAGAAAATCCTGATTTTCAGCAAGAAG<br>GGCTATGAAATTGGTTGA

Supplementary Table 4 (continued).

Protein Product	Protein Sequence Source	Protein Sequence
β-galactosidase (LacS)	UniProt P22498	ATGTACTCATTCCCTAATTCATTTCGGTTCGGCTGGTCCCAGGCCGGTTTCCAAAGTGAGATG<br>GGAACTCCAGGATCTGAGGACCCAAATACTGACTGGTACAAGTGGGTTCACGACCCTGAGAA<br>TATGGCCGCAGGTCTTGTTTCTGGTGACTTGCCTGAGAATGGACCAGGTTACTGGGGTAATTA<br>CAAGACATTCCACGACAATGCTCAAAAGATGGGTCTTAAGATAGCCCGTTTGAATGTTGAGTG<br>GAGTCGGATATTCCCTAATCCTCTTCCTCGTCCACAAAATTTCGACGAGTCCAAGCAAGACGT<br>CACTGAGGTCGAGATCAATGAGAATGAGTTAAAGCGGTTAGACGAGTACGCCAATAAGGACG<br>CCTTAAATCACTACCGTGAGATCTTCAAGGACTTAAAGTCTCGTGGTCTTTACTTCATCCTTAA<br>TATGTACCACTGGCCTTTACCCCTCTGGCTTCACGACCCTATACGTGTACGGCGAGGGGACTT<br>CACGGGTCCATCTGGTTGGCTTTCCACGCGTACAGTTTACGAGTTCGCACGTTTCTCAGCATA<br>CATCGCCTGGAAGTTCGACGACTTGGTTGACGAGTACTCCACTATGAATGAGCCCAATGTAGT<br>TGGCGGTCTTGGGTACGTTGGGGTTAAGTCAGGGTTCCCGCCCGGTTACTTGTCATTCGAGT<br>TATCGCGGCGTGCAATGTACAATATCATCCAAGCCCACGCTCGTGCTTACGACGGGATCAAGT<br>CCGTTAGTAAGAAGCCTGTTGGGATAATATACGCAAATTCCTCTTTCCAACCTCTTACGGACAA<br>GGACATGGAAGCCGTTGAGATGGCAGAGAATGACAATAGATGGTGGTTCTTCGACGCTATAAT<br>CAGAGGAGAGATAACTCGTGGGAATGAGAAGATCGTACGTGACGACTTAAAGGGTAGACTCG<br>ACTGGATAGGTGTCAATTACTACACTCGGACTGTAGTCAAGAGAACTGAGAAGGGTTACGTTA<br>GTTTAGGCGGTTACGGTCACGGGTGTGAGCGGAATTCTGTTTCTCTTGCCGGTTTACCTACAT<br>CGGACTTCGGATGGGAGTTCTTCCCTGAGGGTTTATACGACGTTCTCACTAAGTACTGGAATC<br>GTTACCACTTATACATGTACGTAACTGAGAATGGTATAGCAGACGACGCCGACTACCAAAGAC<br>CATACTACCTTGTCTCACACGTATACCAAGTTCACAGAGCCATCAATTCCGGTGCCGACGTTC<br>GGGGTTACTTACACTGGTCACTTGCAGACAATTACGAGTGGGCATCAGGTTTCTCTATGAGAT<br>TCGGATTACTTAAGGTCGACTACAATACGAAGCGTTTATACTGGCGGCCTTCTGCCCTCGTCT<br>ACAGAGAGATCGCCACTAATGGAGCTATAACAGACGAGATAGAGCACTTGAATAGTGTTCCAC<br>CTGTCAAGCCATTGCGGCACGGCGGATCGGGTGGTGACTACAAGGACCACGACGGTGACTA<br>CAAGGACCACGACATAGACTACAAGGACGACGACGACAAGGGTGGTTCTGGCGGGGTCTCA<br>GGTTGGAGACTTTTCAAGAAGATCTCGTGA

Supplementary Table 4 (continued).

Protein Product	Protein Sequence Source	Protein Sequence
Brolucizumab	KEGG D11083	ATGGAGATTGTGATGACACAAAGCCCGAGCACGCTTTCAGCCTCAGTGGGTGACCGCGTCAT<br>AATCACTTGCCAAGCCAGCGAAATCATCCACTCCTGGCTGGCATGGTATCAACAGAAACCTG<br>GTAAGGCACCGAAACTCCTCATCTATTTGGCATCAACTCTGGCAAGTGGAGTCCCGAGCAGA<br>TTCTCCGGCTCAGGATCGGGTGCCGAATTCACTTTAACCATCTCATCACTCCAACCAGATGAT<br>TTCGCGACATACTACTGCCAAAACGTGTACCTGGCTTCGACTAATGGCGCGAACTTTGGTCAA<br>GGAACGAAATTAACTGTCTTGGGAGGAGGTGGCGGCAGTGGCGGCGGCGGTAGTGGTGGG<br>GGGGGCTCTGGTGGCGGTGGTTCAGAGGTACAATTAGTTGAGAGTGGTGGTGGCCTGGTAC<br>AACCAGGTGGTTCCCTTCGGCTGAGCTGCACAGCCAGTGGCTTCTCTCTCACGGACTACTAC<br>TATATGACCTGGGTCAGACAAGCGCCGGGTAAAGGTCTTGAATGGGTGGGATTCATCGATCC<br>TGACGACGATCCATACTACGCTACTTGGGCCAAGGGGCGTTTTACAATCAGCCGAGACAATTC<br>GAAGAATACATTGTATCTGCAAATGAACAGCCTGCGCGCTGAAGATACCGCCGTTTATTATTGC<br>GCAGGCGGCGACCACAACAGTGGTTGGGGCTTGGATATCTGGGGTCAAGGAACTCTGGTCA<br>CGGTGAGTTCTTAG
Caplacizumab	DrugBank DB06081	ATGGAAGTACAACTGGTTGAAAGCGGTGGTGGTCTGGTACAACCTGGCGGATCACTTCGGCT<br>GAGTTGCGCCGCAAGCGGTCGCACGTTCAGCTACAATCCAATGGGGTGGTTCCGGCAGGCT<br>CCAGGTAAAGGACGCGAATTAGTCGCAGCGATAAGTCGTACAGGCGGTTCCACATATTATCCC<br>GACAGCGTTGAGGGTCGTTTCACGATCTCACGGGACAATGCCAAGCGTATGGTATATCTCCA<br>AATGAACAGTCTTCGCGCCGAGGATACGGCAGTGTACTATTGTGCAGCTGCAGGGGTACGCG<br>CCGAAGACGGAAGAGTACGTACTCTTCCTAGCGAGTATACATTCTGGGGACAGGGTACACAA<br>GTGACCGTGAGCAGCGCTGCCGCAGAGGTTCAACTGGTAGAATCTGGCGGTGGACTGGTG<br>CAACCGGGTGGGTCCCTTAGACTCAGTTGTGCCGCGTCGGGCCGGACATTCTCCTATAACCC<br>AATGGGTTGGTTCCGACAGGCTCCTGGTAAGGGGCGCGAGTTGGTGGCAGCAATTTCACGT<br>ACAGGCGGCTCAACATACTATCCAGACTCAGTAGAGGGTCGGTTCACAATCTCTCGGGACAA<br>CGCCAAGCGGATGGTGTACTTACAGATGAACTCATTACGCGCGGAAGACACGGCCGTTTACT<br>ACTGCGCCGCCGCAGGTGTTCGTGCAGAGGACGGGCGTGTTAGAACGTTACCAAGCGAGTA<br>CACCTTTTGGGGCCAGGGAACCCAAGTAACCGTGTCTAGCTAA

Supplementary Table 4 (continued).

Protein Product	Protein Sequence Source	Protein Sequence
Carbonic anhydrase (CA)	UniProt A0A2I2JKT9	ATGGTCTCATTCTCGTACAATCAACAAAATTTATGGACTGGTGTTTGTAATGCCGGTAATACTG<br>GGCGTCAATCCCCAATAAATATAGTTTTAGCTGACGTAGTCCAATCACCATCTCTTACTCCTCT<br>CGTCTTCAATTCTGAGTGGGACTCAACGAATACAGTTGGAACATTCTCAAATACAGGGCACAA<br>TATCCAATACGACTTAAATTCTACGTCCCCTGACATAACAACTGTAACGCCAATCGGGACATAC<br>AAGTTCTTGCAATTCCACATGCACTGGGGTAATCAAACAGGGGTCGGTTCTGAGCACTTAATC<br>AATGGGGAGCAAAGTGAGGTTGAGATCCACTTCGTACACCAAAAGGTTGGTGCCAGTACTGC<br>CGGTAATATGTTCGGTGTTGTCGGGGTCTTCGCAGACGTCGGAGACATACCCATGACGGGTA<br>TCTGGCAACAATTAAATGCATCCAATGTTCCAGCTGTCGACGACGTTATACTTTCCGACGGTAT<br>ACGTTTCACAGACCTCTTACCATCCAATAGAGACTACTACTACTACGAGGGTGGTCTTACTACA<br>CCACCCTGTACAGAGGCCGTACAATTCTTCTTATTAAAGAATAGAATCACTGTTCCTTCTGCAT<br>ACTTGGCACGTCTCCGTTCCATCGACCAAAATGACGGGCCTGGTGCCATCAATTACCGGGAC<br>ATCCAAAGTTTGAATGGGCGGGTTGTAATGGGATCTGGTTCAAATATGGTTTACGCATCGTGTA<br>TCTCAGTTTTGCTCTTCGCAGTTCTTTCACTCCAACTTGTCTTCTTCGTCGGTGGGAGTGGCG<br>GTGACTACAAGGACCACGACGGGGACTACAAGGACCACGACATCGACTACAAGGACGACGA<br>CGACAAGGGTGGATCTGGTGGTGTATCAGGTTGGCGTCTTTTCAAGAAGATCAGTTAG
Colicin M	Jin, et al. (2018). *Synthetic Biology*.	ATGGAGACCCTGACCGTACACGCGCCATCCCCAAGCACGAATCTTCCCAGCTATGGCAACGG<br>TGCATTCAGTCTCAGTGCTCCGCACGTTCCTGGTGCTGGGCCACTTTTGGTCCAGGTCGTGT<br>ACAGCTTCTTTCAATCCCCAAATATGTGCTTGCAAGCGCTGACTCAATTAGAGGACTATATTAA<br>GAAGCATGGCGCGTCTAATCCACTTACGTTGCAGATAATCTCAACGAACATCGGATATTTCTGT<br>AACGCGGACCGCAATTTGGTATTGCACCCGGGTATCAGCGTGTACGATGCATATCACTTCTCC<br>AAGCCAGCCCCATCTCAGTACGACTATCGCTCCATGAATATGAAGCAGATGTCGGGGAACGTT<br>ACGACGCCTATAGTGGCTCTTGCTCACTACTTGTGGGGTAATGGTGCGGAGCGTTCTGTTAA<br>CATCGCCAACATAGGATTGAAGATTTCTCCCATGAAGATTAATCAAATTAAAGATATTATTAAGTC<br>TGGCGTAGTTGGCACATTCCCGGTCTCCACCAAATTCACCCACGCGACAGGGGATTACAACG<br>TCATCACCGGTGCGTATCTGGGGAATATTACTTTAAAGACTGAGGGTACTTTGACAATCTCTGC<br>AAATGGTAGTTGGACTTACAACGGCGTGGTTAGATCATATGACGATAAGTATGACTTTAACGCT<br>AGTACCCACCGGGGAGTGATTGGTGAGTCTCTGACCCGATTGGGCGCAATGTTCTCGGGGA<br>AGGAATATCAAATTCTTCTGCCAGGTGAAATCCACATAAAGGAATCAGGAAAACGGTGA

Supplementary Table 4 (continued).

Protein Product	Protein Sequence Source	Protein Sequence
CRM197	Addgene 128395	ATGGGCGCAGACGATGTGGTTGATAGTAGTAAGTCGTTCGTAATGGAAAATTTTAGTTCCTACC<br>ACGGTACAAAGCCCGGATACGTGGACAGTATCCAAAAGGGCATCCAAAAGCCCAAATCCGGG<br>ACCCAAGGAAACTACGATGACGATTGGAAGGAGTTCTACTCTACAGACAACAAGTATGACGCC<br>GCTGGTTACTCGGTGGACAATGAGAACCCCCTGTCTGGCAAGGCCGGTGGTGTGGTTAAGG<br>TTACGTACCCTGGCTTAACCAAGGTTTTGGCATTGAAGGTCGACAATGCGGAGACAATTAAGA<br>AGGAGTTAGGGCTGTCCCTGACGGAACCGCTTATGGAACAGGTCGGGACAGAGGAATTCAT<br>CAAGCGCTTTGGCGACGGCGCTAGCCGCGTGGTCTTATCACTGCCGTTTGCTGAGGGTAGT<br>AGTTCCGTTGAGTACATTAATAATTGGGAGCAAGCGAAAGCCTTGTCAGTAGAATTGGAGATC<br>AACTTTGAGACTCGTGGTAAACGTGGGCAAGATGCAATGTACGAGTATATGGCACAAGCGTGT<br>GCAGGAAACCGAGTCCGTCGTTCCGTAGGCAGCAGTCTTTCATGCATCAACTTAGACTGGGA<br>CGTCATACGCGATAAGACCAAAACTAAAATTGAGTCACTGAAGGAGCATGGCCCGATCAAGAA<br>TAAGATGTCCGAAAGCCCAAATAAGACCGTCAGCGAGGAGAAGGCCAAACAATACTTAGAAG<br>AGTTTCACCAAACAGCTCTTGAGCACCCTGAATTAAGCGAACTGAAGACCGTCACCGGGACG<br>AACCCGGTATTCGCGGGAGCGAATTACGCCGCTTGGGCAGTAAATGTAGCTCAAGTAATCGA<br>CAGCGAGACAGCGGACAACCTTGAGAAGACTACTGCCGCACTGTCCATCCTTCCTGGCATTG<br>GTTCAGTTATGGGTATCGCAGACGGCGCAGTACACCACAACACAGAAGAGATTGTAGCACAA<br>AGTATTGCTCTTAGCTCATTGATGGTAGCACAAGCGATACCCCTCGTCGGCGAGCTCGTCGAT<br>ATAGGTTTCGCTGCGTACAACTTCGTTGAGAGTATCATTAACCTTTTCCAAGTAGTCCACAATT<br>CGTACAACCGACCGGCCTACTCCCCCGGCCATAAGACACAACCATTTCTGCATGACGGGTAC<br>GCAGTTTCTTGGAACACAGTCGAGGACAGTATCATACGGACCGGTTTTCAAGGTGAATCCGG<br>ACACGACATCAAGATAACCGCAGAGAACACACCGCTTCCTATAGCTGGGGTGTTGTTACCGA<br>CCATACCCGGCAAGCTGGACGTCAACAAGTCTAAGACGCACATATCAGTGAACGGGAGAAAG<br>ATCCGGATGCGGTGTAGAGCCATTGACGGAGATGTTACCTTCTGCCGGCCTAAGTCTCCGGT<br>GTATGTCGGAAACGGTGTCCATGCCAATCTGCACGTGGCCTTCCACCGTAGTAGCAGTGAGA<br>AGATACACAGCAATGAGATTTCCTCAGACAGCATCGGAGTACTTGGGTATCAAAAGACGGTCG<br>ATCACACTAAGGTTAACTCAAAGCTGTCACTGTTCTTTGAGATTAAGTCTCTTGAGGACCAGA<br>ACGCGACTGGTGGGGACCAAAATGCGACTGGTGGTGATCAAAACGCAACCGGTGGTGACCA<br>GAACGCTACGGTGGATCACCATCACCACCATCATTAG

Supplementary Table 4 (continued).

Protein Product	Protein Sequence Source	Protein Sequence
Factor X	UniProt P00742	ATGGCCAACTCTTTCCTGGAAGAAATGAAGAAGGGACACTTGGAACGGGAATGCATGGAAGA<br>AACGTGTAGCTACGAGGAAGCGCGTGAGGTGTTCGAAGATAGTGACAAAACCAACGAATTTT<br>GGAACAAGTACAAGGACGGGGACCAATGTGAGACTAGCCCGTGCCAAAACCAAGGCAAATG<br>CAAAGACGGCTTAGGAGAGTACACGTGTACATGCTTGGAAGGTTTCGAGGGGAAGAACTGC<br>GAATTGTTTACACGCAAGCTGTGCTCACTCGACAACGGTGATTGCGATCAATTTTGCCATGAA<br>GAGCAAAACTCTGTGGTATGCTCATGTGCCCGCGGATACACTTTAGCCGACAACGGGAAAGC<br>CTGCATTCCAACTGGTCCTTACCCGTGCGGAAAGCAGACGTTAGAGCGGCGTAAACGGAGC<br>GTCGCCCAAGCGACATCTAGCTCAGGAGAAGCACCAGACTCAATTACGTGGAAACCTTACGA<br>TGCAGCGGACCTGGATCCAACCGAAAACCCATTCGACTTACTGGACTTCAACCAAACTCAGC<br>CGGAGCGTGGAGACAACAACTTGACGCGTATCGTCGGTGGACAGGAATGCAAGGACGGCG<br>AATGTCCTTGGCAAGCACTCCTGATTAACGAGGAGAACGAGGGTTTCTGCGGTGGGACTATC<br>TTATCGGAGTTCTACATCCTGACAGCTGCACATTGCTTATACCAAGCAAAGCGTTTTAAAGTAC<br>GGGTTGGTGATCGGAATACCGAACAGGAAGAGGGTGGCGAGGCAGTGCACGAGGTTGAGG<br>TCGTCATCAAACACAACCGATTCACAAAAGAGACCTATGATTTTGACATCGCTGTTTTGCGACT<br>GAAGACCCCTATAACCTTTCGTATGAATGTCGCACCAGCATGCCTGCCTGAGCGAGACTGGG<br>CGGAAAGTACATTGATGACACAAAAGACAGGCATAGTCTCTGGCTTTGGTCGCACACACGAG<br>AAGGGTCGTCAATCGACCCGTCTGAAAATGCTCGAAGTGCCATATGTGGACAGAAACTCTTG<br>TAAACTCAGCAGCTCCTTTATTATAACTCAAAACATGTTCTGCGCAGGTTACGACACCAAGCAA<br>GAGGACGCATGCCAAGGTGATTCTGGCGGACCGCACGTCACACGGTTCAAGGACACATACT<br>TCGTAACTGGTATCGTGTCGTGGGGAGAGGGCTGTGCGCGAAAGGGTAAGTACGGGATATAT<br>ACAAAAGTTACTGCCTTCTTAAAGTGGATCGACCGGTCCATGAAAACTCGCGGCCTTCCCAAA<br>GCGAAGTCGCATGCTCCCGAGGTCATCACATCTTCACCTCTCAAGTAA
FGF2	UniProt P09038	ATGCCAGCACTCCCTGAAGATGGCGGAAGCGGAGCATTTCCACCGGGTCACTTTAAGGACC<br>CTAAGCGTCTGTACTGCAAGAACGGTGGTTTCTTTCTCCGAATCCACCCAGATGGACGTGTT<br>GACGGGGTTCGAGAGAAGAGCGATCCCCATATCAAGCTGCAACTGCAAGCCGAAGAACGGG<br>GTGTTGTTTCTATAAAGGGCGTCTGCGCGAATCGGTATCTCGCCATGAAGGAAGACGGACGA<br>CTTCTGGCATCCAAATGCGTTACCGACGAATGCTTCTTCTTTGAACGGTTGGAGTCCAATAAC<br>TACAACACTTATCGTAGTCGTAAGTACACGAGTTGGTACGTCGCTTTAAAGCGAACCGGGCAA<br>TATAAGTTAGGTAGCAAGACAGGCCCAGGGCAGAAAGCGATTTTGTTCCTGCCAATGTCCGC<br>CAAGTCTTGA

Supplementary Table 4 (continued).

Protein Product	Protein Sequence Source	Protein Sequence
Glutamine synthase (GS)	UniProt Q5SIP0	ATGGGGTACACGAAGGCCGAGATACTTAAGGCTCTTAAGGGTGAGAATGTAAAGTTCTTACGG<br>CTCCAAATCACAGACATCCTTGGTGTAGTTAAGAATGTTGAGGTACCAGAGTCACAATTCGAG<br>AAGGCACTCGACGGAGAGATAATGTTCGACGGATCTTCGATAGAGGGGTTCACTAGAATCGA<br>GGAGAGTGACATGTTACTTCGTCCCGACTACAATACGTTCGTTATATTACCCGACTTAGTCGAG<br>GACCCAAAGCGGGGTCGTGTTGCTCGTTTAATCTGTGACGTATACTACCCTGACGGTCGACC<br>ATTCGAGGGTGACCCTCGGTACGTTTTGAAGAGACAAATCGAGCGTCTTAAGAAGCTTGGGT<br>TCGACAATCTCTACGCAGGGCCCGAGCCCGAGTTCTTCTTGTTCTTGCGTACACCCGAGGGA<br>TTGCCAACTACGGAGACGCACGACCGAGCTGGTTACTTCGACTTAGCACCTATCGACAAAGG<br>GGAAGAGGCCCGTCGTGACATGGTCAATGCTTTAGTTGCAATGGGTTTCGAGATCGAGGCTG<br>CACACCACGAGGTTGCACCAGGGCAACACGAGATAGACTTCAAGTACGCAGACGCCTTGAC<br>TACGGCCGACAATATCGCAACATTCAAGTGGGTAGTAAAGCGAATAGCATTGAATCACGGGTT<br>ACACGCAACATTCCTTCCTAAGCCTATAAGAGGTATCAATGGTTCTGGAATGCACACGCACCT<br>TTCACTTTTCAAGGACGGTGAGAATGCTTTCTACGACCCTAATGCTGAGTACCAACTCTCACA<br>AACAGCATTACACTTCATAGCCGGGTTGTTAGAGCACGCCGCAGGTATGGTAGCCGTTACTAA<br>TCCTTTGGTAAATTCGTACAAGCGTCTTACACCTGGTTACGAGGCCCCCACTAATATAGCCTG<br>GTCTGCTTCAAATAGAAGTGCAATGATCCGTATACCTGCTCGACGTGGTGTTGGGACACGAG<br>CCGAGCTTCGGATGCCTGACCCCAGTTGTAATCCCTACCTTGCACTCGCCGTCATGGCAGCC<br>GCTGGGGCTGACGGGATAGAGCGTAAGCTCCTTCCACCCCCACCTATCCAAAGAAATATATAC<br>CAAATGACAGTTAGAGAGAGACGAAAGCACAAGATACGAGAGTTACCCGGGACTCTCCGGGA<br>AGCATTAGAGGCCTTACGTAAGGACCCAGTTATACGTGAGGCTTTAGGAGAGCACGTCTACAC<br>TCACTTCTTGCAAGCAAAGCAAATGGAGTGGGACGACTACCGTGTCACGGTCCACCAATGGG<br>AGTTGGACCGGTACCTTGCAACATACGGTGGTAGTGGTGGAGACTACAAGGACCACGACGG<br>AGACTACAAGGACCACGACATAGACTACAAGGACGACGACGACAAGGGCGGGTCCGGTGGG<br>GTCAGTGGTTGGAGATTATTCAAGAAGATCAGTTGA
Interferon Gamma	UniProt P01579	ATGCAGGACCCTTATGTCAAGGAAGCTGAGAATCTGAAGAAGTACTTTAATGCGGGTCACTCC<br>GATGTCGCGGACAACGGTACCCTGTTTCTTGGAATCCTGAAGAATTGGAAGGAAGAATCGGA<br>CCGCAAAATTATGCAGTCGCAAATTGTATCTTTCTACTTTAAGCTTTTCAAGAATTTCAAGGAC<br>GACCAATCCATACAAAAGTCCGTCGAGACGATTAAAGAGGACATGAACGTGAAATTCTTTAAC<br>TCTAATAAGAAGAAGCGCGATGATTTCGAGAAATTAACAAACTACAGCGTCACCGATCTGAAC<br>GTCCAACGCAAGGCAATCCACGAGCTCATTCAAGTCATGGCCGAGTTATCACCGGCTGCAAA<br>GACTGGAAAGCGTAAACGGTCCCAAATGTTATTTCGCGGTTGA

Supplementary Table 4 (continued).

Protein Product	Protein Sequence Source	Protein Sequence
momi120	Lauko et al. (2025)	ATGATAAAGGAGTACGAGTTCCCTGCTAAGAAGGCCAAGACAGTAGAGGAAGCAGAGAAGAA<br>TGTTGAAGAGGAGATCGAGCTCATGAAGTCATCAGGTGTTTCATTCTCTGCTAGACGTGAGC<br>GAGTTGTCGTAGTCGGTTACTCGTTGGGAGTCTTCGCAGGTATAATAATGTTCGCCGCAAATA<br>CTACTTACGAGGAAGCCTTGAAGTTAGCAAAGGAGATATTCAAGGAAGCTCTCAAGAATCCAG<br>AGTTAATCGCTCGGCACCTCGCCTTACACCGTAATGCAGAGACTACAGGTAAAGAGGAGTGG<br>CGTCAAGACATAGAGACATGGATAAAGATAATCGAGAAGCGTTTAGGAAAGCCCGTCGACAA<br>GTCAAGAATCTTCATCGCCACGACGAAGGAAGAGGCCGTAAGATTAGCAGAAGAGGCAGTTA<br>AGCTTGGACGAGCAGTTATATACACTCCACCTCACCTCTTGCCTGTTGCAGGGGACGAGATC<br>GTTGAGGTTTTAACTAAGGCCGGTGTTACAGTTCTTGTTAAGGGTGGTATCGGGTCCGGTGTT<br>CCATTAAAGGTATACGAGGCAGGTGGGTCTGGTGGGGACTACAAGGACCACGACGGTGACT<br>ACAAGGACCACGACATCGACTACAAGGACGACGACGACAAGGGCGGTTCGGGCGGTGTCA<br>GTGGATGGCGTTTATTCAAGAAGATATCGTGA
Myoglobin	UniProt P02144	ATGGGCCTTTCCGACGGTGAGTGGCAGCTGGTGTTAAATGTATGGGGCAAGGTGGAAGCAG<br>ATATACCAGGTCATGGACAAGAGGTACTCATACGCCTTTTCAAGGGTCACCCAGAGACTTTAG<br>AAAAGTTCGACAAATTTAAGCATCTTAAGTCGGAAGACGAGATGAAGGCATCGGAAGATTTAA<br>AGAAGCACGGGGCTACTGTGCTTACCGCCCTTGGTGGTATACTGAAGAAGAAGGGTCATCAC<br>GAGGCGGAGATCAAGCCTCTTGCACAAAGTCACGCTACAAAGCACAAAATCCCTGTTAAGTA<br>CCTTGAGTTCATTTCAGAGTGTATCATCCAGGTTTTGCAGAGCAAGCACCCGGGCGACTTCG<br>GCGCAGACGCACAGGGAGCCATGAACAAGGCACTGGAACTCTTTCGTAAGGATATGGCATCC<br>AACTACAAGGAGCTGGGCTTCCAAGGGTAA
Ormutivimab LC	Variable region from Thera-SAbDab Constant IgG1λ region from DrugBank	ATGCAATCTGCCCTTACCCAACCTCGCTCAGTTTCCGGCAGTCCGGGCCAGAGCGTGACCAT<br>ATCCTGTACTGGAACGAGCTCAGACATCGGTGGTTATAACTTCGTGTCTTGGTATCAACAGCA<br>TCCGGGGAAGGCCCCGAAGTTGATGATTTACGACGCAACAAAGCGCCCTAGTGGCGTGCCG<br>GATCGGTTCTCAGGTAGTAAGTCAGGGAATACTGCCTCGCTCACTATAAGTGGATTGCAGGCT<br>GAAGACGAGGCGGACTACTATTGCTGTTCCTACGCAGGCGACTATACTCCTGGTGTTGTGTTT<br>GGTGGTGGCACCAAGTTGACCGTACTTGGCCAACCAAAGGCCAATCCAACGGTGACCTTGT<br>TCCCGCCGTCGAGCGAGGAACTCCAGGCAAATAAAGCGACCCTTGTGTGTTTAATTAGTGAC<br>TTCTACCCTGGCGCGGTGACTGTTGCCTGGAAGGCTGATGGTAGTCCGGTAAAGGCGGGCG<br>TCGAGACTACCAAGCCATCCAAGCAAAGTAATAATAAATACGCAGCTTCCTCGTATCTTTCTTT<br>AACCCCAGAGCAATGGAAGTCCCACCGTTCTTACTCATGTCAAGTGACACACGAAGGGTCTA<br>CCGTCGAGAAAACGGTTGCTCCGACCGAGTGCTCGTGA

Supplementary Table 4 (continued).

Protein Product	Protein Sequence Source	Protein Sequence
Ormutivimab HC	Variable region from Thera-SAbDab<br>Constant IgG1 region from DrugBank	ATGCAAGTCCAGTTAGTCCAAAGTGGTGCTGAGGTGAAGAAGCCGGGTTCGTCAGTAAAAGT<br>TAGCTGCAAGGCTTCGGGCGGGACATTCAATCGTTATACCGTAAACTGGGTCCGACAAGCGC<br>CAGGTCAGGGTCTTGAGTGGATGGGTGGGATCATACCCATCTTCGGGACAGCGAATTATGCG<br>CAACGATTTCAAGGTCGTCTGACCATAACAGCGGATGAGTCTACCTCAACAGCATATATGGAG<br>CTTAGCAGCCTGCGAAGCGATGATACTGCTGTTTACTTTTGTGCTAGAGAGAATTTAGATAATT<br>CTGGGACCTATTACTATTTCAGTGGATGGTTCGACCCGTGGGGTCAGGGTACCCTCGTAACG<br>GTGTCTTCCGCCTCAACTAAAGGACCAAGCGTTTTCCCGTTAGCTCCGTCCAGCAAGAGCAC<br>ATCAGGCGGTACCGCGGCATTAGGGTGCTTAGTGAAGGATTACTTTCCAGAACCGGTGACCG<br>TCTCGTGGAATTCTGGAGCCCTGACCTCTGGAGTTCACACCTTCCCTGCAGTGTTACAGAGC<br>TCCGGATTGTACTCATTAAGTTCAGTGGTAACGGTGCCGTCGTCTTCGCTCGGAACCCAAACT<br>TACATCTGCAACGTGAACCACAAGCCTAGCAACACCAAGGTTGACAAGAAGGTGGAGCCCAA<br>ATCGTGCGATAAGACCCATACCTGTCCACCCTGCCCCGCTCCTGAACTCTTGGGCGGGCCGT<br>CAGTTTTCCTCTTCCCACCTAAACCTAAAGACACGCTTATGATCTCAAGAACCCCTGAGGTGA<br>CTTGTGTGGTAGTTGATGTGTCTCATGAAGATCCCGAGGTAAAGTTCAACTGGTACGTCGACG<br>GCGTCGAAGTGCACAACGCGAAGACTAAACCACGTGAGGAGCAATATAATAGTACGTACCGC<br>GTTGTGTCTGTCCTGACGGTGCTGCACCAAGACTGGCTGAACGGCAAGGAATATAAGTGTAA<br>GGTTTCCAATAAGGCATTACCGGCCCCGATCGAGAAGACAATTAGTAAGGCCAAAGGCCAAC<br>CCCGAGAACCGCAGGTTTACACCTTACCCCCTTCACGCGAGGAGATGACAAAGAACCAAGTC<br>TCATTGACATGCTTAGTTAAGGGCTTCTACCCCAGTGACATAGCGGTTGAGTGGGAGTCAAAT<br>GGGCAACCGGAGAACAACTACAAAACCACGCCACCTGTCCTTGACAGTGATGGCTCCTTCTT<br>TCTGTATTCCAAATTGACCGTGGATAAGTCCCGGTGGCAACAGGGTAATGTTTTCTCCTGTTC<br>AGTAATGCATGAAGCTCTGCACAACCATTATACCCAAAAGTCTCTGAGCCTTTCTCCAGGTAA<br>GTAG
P. damselae DsbC	UniProt D0Z004	ATGGAAAAGAAGATCTGGTCACACCCTCAATTCGAGAAGGGCGGAAGCGGGGAAAACCTTTA<br>TTTTCAATCAGGTGGCGCTAGCCCGGCCGGTGGGGCACCGAATAAAGCCGCAATAACTAAGA<br>AGCTCTCGGCGATCGGCCTGGTTCCCACCGAGATCACTGCATCACAAGTTGCAGGCTTAAAC<br>GAGGTGGTAACGGAGCGCGGAATTGTTTACACCTCAGATGATGGTAACTATTTCATTGTCGGC<br>CACCTTTATGACAACCAAGCAGCGCAGCCAGTTAATGTCACCGAACAAAAGATGGCCAAGATA<br>AACAAGGACAAGTTACAAGGCATGGAAGACGAGATGATTATATATCCGGCGAAGAACCAGAAG<br>CACGTTATCACCGTGTTCACTGACACTACCTGTGGTTATTGCCGCAAACTGCATAACGAGATG<br>CAAGCATACAATGACAAGGGCATTACTGTACGTTATCTCGCTTTCCCGCGAGGCGGTGAGCA<br>ATCAGGCAATTTTATGCAAATGGCTCAAATTTGGGGCGCGAAGGACCGCGCTAAAGCGATGG<br>ATGACGCTAAGAATGGCAGTTTCGACCCTAAGGGTATCACTCCTCGAACGGACCTGATCAAG<br>AAGCACTATGAGCTTGGAGTTGCGATGGGTGTTAATGGGACCCCGGCCATCGTTCTCGAGGA<br>TGGCACGATGATACCTGGGTATCAACCCGCTGCGTCATTATCACAAATGCTGGATGCACAACA<br>ATCAAAGAAGTAA

Supplementary Table 4 (continued).

Protein Product	Protein Sequence Source	Protein Sequence
Protein D	Addgene 128391	ATGAAGTCAGACAAGATCATTATCGCGCATCGCGGTGCCTCCGGCTACTTGCCGGAGCATAC<br>TTTGGAGAGCAAAGCCTTAGCATTTGCCCAACAAGCGGATTACTTGGAACAAGACTTGGCCAT<br>GACTAAGGATGGGCGTCTGGTGGTGATTCACGACCATTTTCTTGATGGTCTTACGGATGTAGC<br>TAAGAAGTTTCCGCATCGCCATCGTAAGGACGGGCGCTATTATGTAATCGATTTTACCTTGAAG<br>GAAATTCAAAGCTTGGAGATGACCGAAAACTTTGAGACGAAGGATGGGAAGCAAGCGCAAGT<br>CTACCCGAATCGTTTTCCCTTATGGAAAAGCCATTTTCGCATCCATACTTTTGAGGATGAGATT<br>GAGTTTATCCAAGGGCTGGAAAAGTCTACTGGCAAGAAAGTTGGGATCTATCCTGAGATCAAG<br>GCACCGTGGTTTCATCATCAAAACGGTAAGGACATCGCCGCTGAGACCTTAAAAGTACTGAA<br>GAAGTATGGCTACGATAAAAAGACTGACATGGTTTACCTTCAAACGTTCGATTTTAATGAGTTG<br>AAACGTATTAAAACAGAATTATTGCCCCAGATGGGGATGGATTTGAAGTTGGTTCAGTTAATCG<br>CCTACACCGACTGGAAGGAAACGCAGGAGAAGGATCCCAAAGGATACTGGGTGAATTATAAT<br>TATGATTGGATGTTCAAGCCAGGAGCAATGGCTGAAGTAGTAAAATATGCGGATGGTGTAGGC<br>CCCGGCTGGTATATGCTTGTGAACAAAGAAGAAAGCAAGCCTGATAATATCGTCTATACTCCCT<br>TAGTCAAAGAACTGGCCCAATATAACGTAGAAGTTCACCCTTACACCGTCCGTAAGGACGCCT<br>TACCGGAGTTTTTTACTGACGTCAATCAAATGTATGATGCCTTGCTGAACAAGAGTGGAGCGA<br>CAGGCGTCTTCACCGACTTTCCAGACACTCTCGAGGATCAGAACGCGACCGGCGGTGACCA<br>AAATGCCACAGGTGGCGATCAAAACGCCACCGGCGGTGACCAGAATGCGACAGTCGACcatc<br>accatcatcaccatTAA
Ranibizumab HC	DrugBank DB00054	ATGGAAGTTCAGCTTGTGGAATCAGGTGGCGGATTAGTACAACCCGGTGGTAGTTTACGTCT<br>GTCGTGTGCTGCCTCGGGCTATGATTTTACTCATTACGGTATGAATTGGGTAAGACAAGCCCC<br>GGGAAAGGGGTTAGAGTGGGTCGGCTGGATCAACACCTACACCGGCGAGCCGACCTACGCT<br>GCTGACTTCAAGCGTCGGTTCACGTTCAGTCTGGACACTTCAAAGAGCACAGCTTACCTTCA<br>AATGAATTCACTTCGCGCAGAGGATACAGCCGTTTATTACTGTGCCAAATATCCGTATTATTACG<br>GCACGAGTCACTGGTACTTTGACGTTTGGGGACAAGGCACATTAGTTACTGTCTCGTCTGCG<br>AGCACCAAAGGGCCAAGCGTTTTCCCCCTCGCTCCATCAAGCAAGTCTACTTCCGGTGGCAC<br>GGCGGCATTGGGGTGTCTCGTTAAAGACTACTTCCCGGAGCCAGTTACGGTGTCGTGGAATT<br>CTGGCGCACTTACATCTGGAGTGCACACATTTCCAGCAGTTTTACAATCTTCAGGCCTTTACA<br>GCTTATCTTCTGTAGTCACAGTGCCTTCGTCTAGTCTCGGAACACAGACTTATATCTGCAACGT<br>GAATCACAAGCCATCAAACACAAAAGTCGATAAGAAAGTAGAACCGAAGTCATGTGACAAGAC<br>GCACTTATAA

Supplementary Table 4 (continued).

Protein Product	Protein Sequence Source	Protein Sequence
Ranibizumab LC	DrugBank DB00054	ATGGACATTCAACTCACACAAAGTCCCAGTTCCCTGTCCGCGAGTGTGGGCGACCGGGTGA<br>CAATAACCTGTTCAGCCTCGCAGGACATCTCGAATTACTTAAACTGGTATCAGCAAAAGCCAG<br>GCAAGGCACCTAAGGTTCTGATTTACTTTACTTCTAGTCTGCACTCTGGTGTTCCGAGCCGGT<br>TTTCTGGCTCAGGCTCGGGGACAGATTTCACATTAACTATCAGCTCATTGCAGCCGGAAGATT<br>TTGCAACCTACTACTGTCAGCAATACTCTACGGTACCCTGGACTTTTGGCCAAGGCACTAAAG<br>TGGAAATTAAACGTACTGTCGCCGCTCCATCTGTTTTCATTTTCCCGCCGTCAGACGAACAAC<br>TCAAGTCTGGAACGGCTTCCGTAGTTTGCTTACTGAACAACTTTTACCCACGTGAGGCGAAG<br>GTACAATGGAAGGTGGACAATGCACTTCAAAGCGGCAACTCTCAGGAGTCTGTCACTGAGCA<br>AGATTCCAAGGACTCCACTTACAGCCTCTCGAGTACACTGACGCTGTCCAAAGCGGATTACG<br>AGAAGCACAAGGTCTACGCTTGCGAGGTCACACACCAGGGCTTATCATCACCAGTCACTAAG<br>TCTTTTAACCGAGGCGAGTGTTGA
sfGFP	Addgene 102634	ATGAGCAAAGGTGAAGAACTGTTTACCGGCGTTGTGCCGATTCTGGTGGAACTGGATGGCGA<br>TGTGAACGGTCACAAATTCAGCGTGCGTGGTGAAGGTGAAGGCGATGCCACGATTGGCAAA<br>CTGACGCTGAAATTTATCTGCACCACCGGCAAACTGCCGGTGCCGTGGCCGACGCTGGTGA<br>CCACCCTGACCTATGGCGTTCAGTGTTTTAGTCGCTATCCGGATCACATGAAACGTCACGATT<br>TCTTTAAATCTGCAATGCCGGAAGGCTATGTGCAGGAACGTACGATTAGCTTTAAAGATGATG<br>GCAAATATAAAACGCGCGCCGTTGTGAAATTTGAAGGCGATACCCTGGTGAACCGCATTGAAC<br>TGAAAGGCACGGATTTTAAAGAAGATGGCAATATCCTGGGCCATAAACTGGAATACAACTTTAA<br>TAGCCATAATGTTTATATTACGGCGGATAAACAGAAAAATGGCATCAAAGCGAATTTTACCGTTC<br>GCCATAACGTTGAAGATGGCAGTGTGCAGCTGGCAGATCATTATCAGCAGAATACCCCGATTG<br>GTGATGGTCCGGTGCTGCTGCCGGATAATCATTATCTGAGCACGCAGACCGTTCTGTCTAAA<br>GATCCGAACGAAAAAGGCACGCGGGACCACATGGTTCTGCACGAATATGTGAATGCGGCAG<br>GTATTACGTGGAGCCATCCGCAGTTCGAAAAATAA

Supplementary Table 4 (continued).

Protein Product	Protein Sequence Source	Protein Sequence
Streptokinase	UniProt P96471	ATGATAGCTGGACCTGAATGGTTGTTGGGCCGCCCTAGTGTGAATAACTCGCAATTAGTCGTG<br>TCCGTAGCGGGAACAGTTGAGGGTACTAATCAAGAAATCTCCCTGAAATTCTTCGAAATTGAT<br>TTAACTTCGCGTCCCGCTCAAGGCGGTAAAACGGAGCAGGGATTGCGTCCCAAGTCCAAAC<br>CTCTGGCGACCGACAAAGGCGCAATGTCTCATAAGCTTGAGAAAGCAGACCTTCTTAAAGCG<br>ATCCAGGAACAATTGATTGCTAACGTTCACTCCAACGACGGTTATTTCGAGGTAATCGACTTTG<br>CCTCTGATGCCACCATCACCGACCGTAATGGTAAAGTTTACTTTGCCGACCGTGACGATAGTG<br>TAACTCTGCCGACACAACCAGTCCAAGAGTTTCTGCTGTCCGGTCACGTCCGGGTACGGCC<br>CTATCGGCCCAAGGCGGTACACAACAGTGCCGAACGAGTCAATGTCAACTATGAAGTCAGCT<br>TTGTTAGCGAGACGGGAAATTTGGACTTCACACCTAGTCTCAAGGAGCAATACCACCTCACCA<br>CCTTAGCCGTTGGCGACTCACTTTCATCCCAGGAGTTAGCGGCAATAGCCCAGTTCATCCTTT<br>CTAAGAAACACCCCGACTACATAATTACGAAGCGGGATTCATCAATTGTGACGCACGACAATG<br>ATATCTTCAGAACGATACTGCCGATGGACCAAGAGTTCACCTACCATATCAAGGACAGAGAGC<br>AAGCCTACAAAGCGAATTCTAAAACCGGCATTGAGGAGAAAACTAATAATACAGACTTGATTTC<br>AGAGAAGTACTATATTCTTAAGAAAGGGGAAAAGCCGTATGATCCTTTCGACCGTAGCCACTT<br>GAAGCTGTTCACTATTAAGTATGTAGATGTAGACACAAAGGCTCTGCTCAAAAGCGAACAACT<br>TCTTACAGCAAGTGAACGCAACCTTGATTTCAGAGATCTTTATGATCCACGCGACAAGGCCAA<br>GCTGCTTTACAACAATCTTGACGCCTTCGGTATCATGGGATACACCCTGACCGGTAAAGTAGA<br>GGATAATCACGATGATACCAATCGGATTATCACTGTTTACATGGGGAAGAGACCGGAAGGCGA<br>GAATGCATCATACCACCTTGCGTACGACAAGGACCGGTATACAGAAGAAGAACGGGAAGTATA<br>CTCATACCTTCGTGACACCGGTACCCCGATCCCAGATAATCCGAAAGATAAGTGA
Trastuzumab Fc	DrugBank DB00072	ATGGAACCGAAATCTTGTGACAAAACTCACACCTGCCCACCGTGCCCGGCACCTGAACTCCT<br>GGGGGGACCGTCAGTCTTCCTCTTCCCCCCAAAACCCAAGGACACCCTCATGATCTCCCGG<br>ACCCCTGAGGTCACATGCGTGGTGGTGGACGTGAGCCACGAAGACCCTGAGGTCAAGTTCA<br>ACTGGTACGTGGACGGCGTGGAGGTGCATAATGCCAAGACAAAGCCGCGGGAGGAGCAGTA<br>CAACAGCACGTACCGTGTGGTCAGCGTCCTCACCGTCCTGCACCAGGACTGGCTGAATGGC<br>AAGGAGTACAAGTGCAAGGTCTCCAACAAAGCCCTCCCAGCCCCCATCGAGAAAACCATCTC<br>CAAAGCCAAAGGGCAGCCCCGAGAACCACAGGTGTACACCCTGCCCCCATCCCGGGATGAG<br>CTGACCAAGAACCAGGTCAGCCTGACCTGCCTGGTCAAAGGCTTCTATCCCAGCGACATCG<br>CCGTGGAGTGGGAGAGCAATGGGCAGCCGGAGAACAACTACAAGACCACACCTCCCGTGCT<br>GGACTCCGACGGCTCCTTCTTCCTCTACAGCAAGCTCACCGTGGACAAGAGCAGGTGGCAG<br>CAGGGGAACGTCTTCTCATGCTCCGTGATGCATGAGGCTCTGCACAACCACTACACGCAGAA<br>GAGCCTCCCCCTGTCTCCGGGTAAACTCGAGTAA

Supplementary Table 4 (continued).

Protein Product	Protein Sequence Source	Protein Sequence
Trastuzumab HC	DrugBank DB00072	ATGGAAGTTCAATTGGTAGAATCCGGAGGAGGTTTGGTCCAACCAGGTGGATCCCTGCGCCT<br>GAGCTGCGCCGCCAGTGGGTTTAACATAAAAGATACATATATTCATTGGGTTCGTCAGGCCCC<br>TGGCAAGGGCTTGGAGTGGGTTGCACGGATATATCCAACCAATGGGTATACTCGATACGCAG<br>ACTCCGTCAAGGGGCGCTTTACGATCAGCGCCGATACATCAAAGAACACAGCATACCTGCAA<br>ATGAACTCTCTCCGAGCAGAGGATACCGCAGTTTATTACTGTTCTCGGTGGGGTGGGGACGG<br>ATTTTACGCCATGGATTACTGGGGACAGGGTACATTAGTTACCGTATCTTCAGCCAGCACCAA<br>GGGACCTTCAGTGTTTCCACTTGCACCGAGTTCTAAGAGTACTAGCGGTGGCACGGCTGCG<br>CTGGGTTGCTTAGTAAAGGATTATTTTCCCGAGCCAGTGACCGTTAGTTGGAACAGCGGTGC<br>GCTTACTAGTGGTGTTCATACATTTCCCGCGGTGCTTCAAAGTTCGGGTTTATATTCCTTATCAT<br>CCGTAGTTACGGTCCCGAGCTCAAGCCTTGGAACCCAGACTTATATATGCAACGTCAACCACA<br>AGCCTTCCAACACAAAGGTTGATAAGAAGGTCGAACCCAAGTCCTGCGACAAGACACACACA<br>TGTCCCCCATGTCCCGCTCCGGAGTTGTTGGGTGGACCGTCTGTTTTCTTATTCCCACCAAA<br>GCCTAAAGATACCTTAATGATCTCCCGCACCCCAGAAGTGACATGCGTTGTGGTCGACGTCTC<br>TCACGAGGACCCTGAGGTGAAATTTAACTGGTACGTAGACGGCGTCGAAGTACATAATGCGA<br>AGACGAAACCTCGTGAAGAGCAATACAACAGCACCTACCGCGTCGTCTCAGTACTGACTGTG<br>CTCCACCAAGATTGGCTGAACGGAAAAGAATACAAGTGTAAGGTGTCCAACAAAGCTTTGCC<br>AGCTCCGATAGAGAAAACAATCAGTAAGGCGAAGGGTCAACCACGAGAGCCGCAAGTTTACA<br>CATTGCCGCCGTCTCGGGAAGAAATGACGAAGAATCAAGTCTCATTAACCTGCCTTGTGAAG<br>GGTTTCTACCCGTCAGACATAGCTGTTGAGTGGGAGAGTAACGGACAGCCAGAGAATAATTA<br>CAAAACAACACCGCCTGTGCTGGATTCTGACGGCTCATTCTTTCTCTACTCGAAGTTAACAGT<br>GGATAAAAGCAGATGGCAACAGGGGAACGTATTCTCTTGTTCCGTCATGCACGAAGCCCTTC<br>ATAATCATTACACCCAAAAGTCGCTTAGCCTCTCACCGGGCAAGTGA
Trastuzumab LC	DrugBank DB00072	ATGGACATTCAGATGACCCAGAGTCCTTCTTCACTGAGCGCATCGGTAGGCGATCGGGTTAC<br>CATTACTTGCAGAGCCTCGCAGGACGTTAACACAGCTGTTGCGTGGTACCAACAAAAGCCGG<br>GTAAAGCGCCGAAACTTCTTATATATAGCGCTTCTTTCCTTTACAGCGGAGTTCCTAGTCGGTT<br>CTCAGGGTCGCGTTCCGGCACCGACTTCACCTTGACCATTAGCTCCCTTCAGCCAGAGGATT<br>TCGCCACTTATTACTGCCAACAGCACTACACTACTCCGCCGACATTCGGGCAGGGAACGAAA<br>GTAGAGATTAAGCGAACCGTCGCAGCGCCCAGTGTCTTTATTTTCCCGCCATCGGATGAGCA<br>GCTGAAATCGGGTACAGCCTCTGTCGTCTGCCTTCTCAACAACTTTTACCCGCGCGAGGCCA<br>AAGTACAATGGAAGGTGGACAATGCCCTGCAATCCGGAAACAGTCAGGAATCCGTGACCGAG<br>CAAGACTCAAAGGACTCAACTTACAGCCTCAGTTCAACGCTCACCCTTAGTAAGGCGGACTA<br>CGAGAAACATAAGGTGTACGCGTGCGAGGTAACTCACCAAGGTCTTAGCTCGCCTGTGACGA<br>AATCTTTTAATCGCGGTGAATGCTAA

Supplementary Table 4 (continued).

Protein Product	Protein Sequence Source	Protein Sequence
TRI2-2_NoTags	Hunt, et al. (2022). *Science Translational Medicine.*	ATGGAATTGGAAGAGCAGGTAATGCATGTATTAGACCAAGTGTCTGAGCTCGCCCACGAACT GCTGCACAAGCTGACCGGCGAAGAGCTCGAGCGAGCAGCGTACTTCAACTGGTGGGCGAC TGAAATGATGTTGGAGTTAATCAAGTCCGACGATGAGCGTGAGATTCGCGAGATTGAAGAGG AAGCCAGACGCATCTTGGAGCATCTCGAGGAGTTAGCGCGTAAGGGCGGTAGCGAAGCGTT AGAGGAACTGGAGAAGGCGCTGCGCGAATTGAAGAAGTCCACGGATGAACTGGAGCGTTCG ACAGAGGAGTTAGAGAAGAACCCCAGCGAGGACGCTCTGGTTGAAAATAACCGCCTCATCGT GGAGAATAACAAGATAATAGTTGAAGTGCTGCGTATAATAGCCAAGGTTCTTAAATGA
TRI2-2_2xStrep	Hunt, et al. (2022). *Science Translational Medicine.*	ATGGAGAAAAAAATCGAATTGGAGGAGCAAGTGATGCATGTCTTGGATCAAGTAAGTGAATTA GCGCATGAGCTGTTGCACAAGCTTACAGGGGAAGAGCTGGAGCGTGCTGCGTATTTTAACTG GTGGGCAACAGAAATGATGCTTGAATTGATCAAAAGTGATGACGAGCGCGAAATCCGCGAGA TTGAAGAGGAGGCTCGCCGTATCTTAGAGCACTTAGAAGAGCTTGCTCGTAAGGGGGGGTC CGAAGCCCTGGAAGAATTGGAAAAAGCACTTCGCGAATTAAAAAAATCCACAGACGAACTTG AGCGCTCTACAGAGGAGTTAGAGAAGAATCCATCCGAAGACGCTTTGGTCGAGAATAATCGC TTGATCGTAGAAAACAATAAGATCATCGTAGAGGTTCTGCGTATTATCGCCAAAGTCTTAAAGG GTGGCTCGGGATCTTCAGGTTCGGCCTGGAGCCACCCACAGTTTGAGAAGGGAGGAGGAA GTGGGGGAGGGTCAGGTGGCTCAGCGTGGTCACACCCTCAATTTGAGAAGTAA

Supplementary Table 4 (continued).

Protein Product	Protein Sequence Source	Protein Sequence
Type III polyketide synthase RppA	Sword et al. (2024)	ATGGCTACACTTTGTCGTCCCGCCATCGCTGTACCAGAGCACGTTATAACTATGCAACAAACA<br>TTAGACTTGGCCCGTGAGACTCACGCAGGTCACCCACAACGTGACCTTGTTCTCAGATTGAT<br>CCAAAATACTGGTGTCCAAACTCGACACCTTGTACAACCTATCGAGAAGACATTGGCTCACCC<br>TGGTTTCGAGGTCCGTAATCAAGTTTACGAGGCTGAGGCAAAGACTCGTGTCCCAGAGGTTG<br>TCAGACGTGCATTAGCAAATGCAGAGACGGAGCCTTCAGAGATAGACCTCATCGTTTACGTAT<br>CCTGTACGGGTTTCATGATGCCTAGTCTTACGGCTTGGATCATCAATTCTATGGGGTTCCGAC<br>CCGAGACACGTCAACTCCCCATCGCACAATTGGGGTGTGCTGCTGGTGGTGCCGCTATCAAT<br>CGTGCACACGACTTCTGTGTCGCTTACCCTGACAGTAATGTTTTGATCGTCTCATGTGAGTTC<br>TGTTCATTATGTTACCAACCAACGGACATCGGGGTTGGTTCCCTTTTAAGTAATGGGTTATTCG<br>GGGACGCCTTATCGGCTGCAGTAGTTCGGGGACAAGGTGGGACTGGGATGCGATTAGAGCG<br>GAATGGTTCGCACTTAGTTCCCGACACGGAAGACTGGATAAGTTACGCTGTCAGAGACACAG<br>GTTTCCACTTCCAATTAGACAAGCGAGTTCCCGGTACTATGGAGATGCTCGCACCTGTTTTAC<br>TCGACCTTGTTGACCTTCACGGGTGGTCCGTACCAAATATGGACTTCTTCATAGTCCACGCAG<br>GTGGACCACGGATATTAGACGACCTCTGTCACTTCCTTGACCTTCCACCAGAGATGTTCCGAT<br>ACAGTCGTGCAACGTTGACGGAGCGTGGTAATATCGCATCATCGGTCGTCTTCGACGCCTTA<br>GCCCGACTCTTCGACGACGGTGGGGCAGCAGAGTCGGCACAAGGATTGATCGCCGGATTC<br>GGTCCAGGGATAACAGCCGAGGTAGCTGTAGGGTCATGGGCTAAAGAGGGACTCGGTGCCG<br>ACGTAGGGCGTGACTTGGACGAGCTTGAGCTTACGGCAGGTGTAGCCCTTTCAGGAGGAGG<br>ATCAGGTGGTGACTACAAGGACCACGACGGAGACTACAAGGACCACGACATCGACTACAAG<br>GACGACGACGACAAGGGCGGTAGTGGTGGAGTCTCTGGTTGGCGGCTCTTCAAGAAGATAT<br>CTTGA
Xyloglucan-specific endo-beta-1,4-glucanase (XEG1)	UniProt G4ZHR2	ATGGGTGACTACTGTGGTCAATGGGACTGGGCTAAGAGTACAAATTACATCGTCTACAATAATT<br>TGTGGAATAAGAATGCCGCAGCTTCTGGGTCCCAATGTACTGGAGTTGACAAGATATCCGGAT<br>CTACGATCGCCTGGCACACTTCGTACACGTGGACGGGTGGTGCCGCCACTGAGGTCAAGTC<br>TTACTCAAATGCAGCACTCGTTTTCTCAAAGAAGCAAATAAAGAATATCAAGTCGATCCCTACA<br>AAGATGAAGTACTCATACAGTCACTCATCTGGTACTTTCGTCGCAGACGTCTCTTACGACCTC<br>TTCACGAGTTCTACAGCCAGTGGTTCAAATGAGTACGAGATCATGATCTGGTTAGCTGCCTAC<br>GGTGGGGCCGGGCCTATCTCCAGTACAGGTAAGGCCATCGCCACTGTAACTATAGGATCCAA<br>TTCATTCAAGCTTTACAAGGGACCCAATGGTTCAACTACGGTCTTCTCCTTCGTTGCAACGAA<br>GACTATCACAAATTTCTCGGCTGACTTACAAAAGTTCTTGTCGTACTTGACAAAGAATCAAGGT<br>CTTCCCAGTTCTCAATACTTGATCACTTTGGAAGCCGGGACTGAGCCATTCGTTGGAACGAAT<br>GCAAAGATGACTGTTTCATCATTCTCCGCTGCCGTAAATGGTGGTTCCGGCGGAGACTACAA<br>GGACCACGACGGAGACTACAAGGACCACGACATCGACTACAAGGACGACGACGACAAGGG<br>CGGTAGTGGTGGAGTATCGGGATGGCGATTGTTCAAGAAGATCTCTTGA

Supplementary Table 4 (continued).

Protein Product	Protein Sequence Source	Protein Sequence
vtPA	UniProt P00750<br>Truncated based on:<br>Gething, et al. (1988)<br>*The EMBO Journal.*<br>Yin & Swartz. (2004).<br>*Wiley InterScience*.	ATGGGAAACAGCGATTGTTATTTTGGGAACGGCTCCGCATATCGTGGCACACACAGTTTGACA<br>GAAAGTGGTGCGTCATGTCTTCCTTGGAATTCAATGATATTAATCGGTAAAGTTTACACGGCGC<br>AAAATCCTAGCGCCCAAGCACTTGGACTGGGCAAGCATAATTATTGCCGCAACCCAGACGGGG<br>ACGCAAAGCCGTGGTGCCACGTATTAAAGAACCGCCGCCTGACATGGGAATATTGTGACGTAC<br>CTTCATGCAGTACATGCGGCCTGCGCCAATACTCCCAGCCGCAGTTCCGCATTAAGGGTGGG<br>CTTTTCGCAGATATCGCATCCCATCCGTGGCAAGCAGCCATTTTCGCTAAGCACAGACGTAGC<br>CCAGGAGAAAGATTTCTTTGCGGTGGAATTTTAATCTCATCGTGCTGGATACTCTCAGCTGCGC<br>ACTGTTTCCAGGAGCGCTTCCCACCACATCACCTTACGGTAATTTTAGGACGCACATATCGGGT<br>AGTACCTGGCGAGGAAGAGCAGAAGTTCGAGGTGGAGAAATATATTGTGCACAAGGAATTTGA<br>CGACGATACGTATGACAACGACATAGCACTGCTGCAACTGAAGTCTGATTCGTCACGGTGCGC<br>TCAGGAGTCTTCCGTCGTCCGCACCGTCTGTTTACCCCCTGCTGACCTGCAACTGCCAGATTG<br>GACTGAGTGTGAGCTCTCCGGCTACGGAAAACACGAAGCCTTAAGCCCTTTCTACTCGGAAC<br>GTCTTAAGGAAGCGCATGTTCGCCTCTACCCCTCTTCCAGATGCACATCTCAACACTTGTTGAA<br>TCGGACCGTTACAGACAATATGTTGTGCGCCGGAGACACGCGTTCAGGCGGACCGCAAGCCA<br>ATTTACATGACGCATGCCAGGGAGACTCAGGCGGTCCTTTGGTTTGTTTAAACGATGGCCGGA<br>TGACGCTCGTAGGTATCATTTCTTGGGGACTGGGTTGCGGGCAAAAGGACGTTCCCGGAGTC<br>TACACAAAAGTCACCAACTACTTGGACTGGATACGGGACAACATGCGCCCATGA
