# Supplementary material for: Design-driven optimization of low-cost reagent formulations for reproducible and high-yielding cell-free gene expression
Source: Nat Commun. 2026 Mar 5;17:3478. doi: 10.1038/s41467-026-69605-8 (PMC13079839; doi:10.1038/s41467-026-69605-8)
Supplement: Supplementary file 2 — Description of Additional Supplementary Files [file 41467_2026_69605_MOESM2_ESM.pdf]

Description of Additional Supplementary Files:

Supplementary Data 1: Excel file providing optimization campaign design strategies and model fit details.

Supplementary Data 2: Excel file containing all tested reagent formulations and their corresponding sfGFP yield, $\$/L_{CFE}$, and $\$/g_{protein}$.
