# Supplementary material for: Design-driven optimization of low-cost reagent formulations for reproducible and high-yielding cell-free gene expression
Source: Nat Commun. 2026 Mar 5;17:3478. doi: 10.1038/s41467-026-69605-8 (PMC13079839; doi:10.1038/s41467-026-69605-8)
Supplement: Supplementary file 5 — Reporting Summary [file 41467_2026_69605_MOESM5_ESM.pdf]

nature portfolio

Corresponding author(s): Michael C. Jewett & Ashty S. Karim

Last updated by author(s): 12/15/2025

Reporting Summary

Nature Portfolio wishes to improve the reproducibility of the work that we publish. This form provides structure for consistency and transparency in reporting. For further information on Nature Portfolio policies, see our Editorial Policies and the Editorial Policy Checklist.

Statistics

For all statistical analyses, confirm that the following items are present in the figure legend, table legend, main text, or Methods section.

n/a	Confirmed	
☐	☒	The exact sample size (*n*) for each experimental group/condition, given as a discrete number and unit of measurement
☐	☒	A statement on whether measurements were taken from distinct samples or whether the same sample was measured repeatedly
☐	☒	The statistical test(s) used AND whether they are one- or two-sided<br>*Only common tests should be described solely by name; describe more complex techniques in the Methods section.*
☒	☐	A description of all covariates tested
☐	☒	A description of any assumptions or corrections, such as tests of normality and adjustment for multiple comparisons
☐	☒	A full description of the statistical parameters including central tendency (e.g. means) or other basic estimates (e.g. regression coefficient) AND variation (e.g. standard deviation) or associated estimates of uncertainty (e.g. confidence intervals)
☐	☒	For null hypothesis testing, the test statistic (e.g. *F*, *t*, *r*) with confidence intervals, effect sizes, degrees of freedom and *P* value noted<br>*Give P values as exact values whenever suitable.*
☒	☐	For Bayesian analysis, information on the choice of priors and Markov chain Monte Carlo settings
☒	☐	For hierarchical and complex designs, identification of the appropriate level for tests and full reporting of outcomes
☒	☐	Estimates of effect sizes (e.g. Cohen's *d*, Pearson's *r*), indicating how they were calculated

Our web collection on statistics for biologists contains articles on many of the points above.

Software and code

Policy information about availability of computer code

Data collection	All data were collected using stated instruments and associated commercially available software. Commercial software used includes: BioTek Gen5 version 3.14 (Agilent) for acquiring all plate reader data, MicroBeta2 Windows Workstation version 6.0.0.0 (PerkinElmer) for acquiring 14C-leucine radioactive count data, Typhoon FLA 7000 control software version 1.2.1.93 (GE) for acquiring autoradiogram images, ChemStation version B.04.03 (Agilent) for HPLC data acquisition, and Echo 525 Liquid Handler and Plate Reformat version 2.7.2 (Beckman Coulter) for Echo 525 operation. Software for bioreactor operation is described in the indicated reference.
Data analysis	Data were visualized and analyzed using GraphPad Prism 10.5.0 and Microsoft Excel version 16.43 or 2506. Densitometry analysis was performed using ImageJ 0.5.8. Plasmidsaurus genome sequencing results were visualized and analyzed with SnapGene 5.0.8. HPLC spectra were analyzed with ChemStation version B.04.03 (Agilent). Design of Experiments analysis was performed with JMP Pro 16.

For manuscripts utilizing custom algorithms or software that are central to the research but not yet described in published literature, software must be made available to editors and reviewers. We strongly encourage code deposition in a community repository (e.g. GitHub). See the Nature Portfolio guidelines for submitting code & software for further information.

Data

Policy information about availability of data

All manuscripts must include a data availability statement. This statement should provide the following information, where applicable:

- Accession codes, unique identifiers, or web links for publicly available datasets
- A description of any restrictions on data availability
- For clinical datasets or third party data, please ensure that the statement adheres to our policy

Source data for all figures are provided with this paper in the Source Data file (main text figures), Supplementary Data 3 file (supplementary figures), and Supplementary Data 4 (unedited autoradiograms). Data regarding reagent optimization campaign design strategies and optimization formulas are provided in the Supplementary Information/Source Data files. The genomic sequencing data for the BL21 Star (DE3) Δgor strain has been deposited in the NCBI database under BioProject accession code PRJNA1306108 [https://www.ncbi.nlm.nih.gov/bioproject/1306108].

Research involving human participants, their data, or biological material

Policy information about studies with human participants or human data. See also policy information about sex, gender (identity/presentation), and sexual orientation and race, ethnicity and racism.

Reporting on sex and gender	None to report.
Reporting on race, ethnicity, or other socially relevant groupings	None to report.
Population characteristics	None to report.
Recruitment	None to report.
Ethics oversight	None to report.

Note that full information on the approval of the study protocol must also be provided in the manuscript.

Field-specific reporting

Please select the one below that is the best fit for your research. If you are not sure, read the appropriate sections before making your selection.

- [x] Life sciences
- [ ] Behavioural & social sciences
- [ ] Ecological, evolutionary & environmental sciences

For a reference copy of the document with all sections, see nature.com/documents/nr-reporting-summary-flat.pdf

Life sciences study design

All studies must disclose on these points even when the disclosure is negative.

Sample size	All data are presented as the mean of n = 3 biological replicates, unless otherwise stated. Sample sizes were determined based on precedent (minimum number to determine standard deviation) and were not calculated via alternative statistical methods.
Data exclusions	None to report.
Replication	All attempts at replication were successful and are stated throughout the manuscript text and figure legends, including replication across different laboratory sites. Biological replicates were composed of individual cell-free expression reactions that were then analyzed as stated, with each data point representing the result from a distinct expression reaction. Replication experiments were set up on separate days using separate sets of reagents and found to be consistent, as demonstrated in the manuscript text and figures.
Randomization	No randomization was performed. Covariates were controlled by using either the same lot of small molecule reagents and cell lysate for each reaction, varying only reagent composition or plasmid DNA, or by using reagent lots that were validated against a set of laboratory standards to ensure consistent performance.
Blinding	No blinding was performed. All experiments required experimenter knowledge for proper set-up. Additionally, all data analysis was done based on objective data measurements that could not be directly influenced by the researcher (e.g., plate reader values and statistical analysis).

Reporting for specific materials, systems and methods

We require information from authors about some types of materials, experimental systems and methods used in many studies. Here, indicate whether each material, system or method listed is relevant to your study. If you are not sure if a list item applies to your research, read the appropriate section before selecting a response.

Materials & experimental systems

n/a	Involved in the study	
☒	☐	Antibodies
☒	☐	Eukaryotic cell lines
☒	☐	Palaeontology and archaeology
☒	☐	Animals and other organisms
☒	☐	Clinical data
☒	☐	Dual use research of concern
☒	☐	Plants

Methods

n/a	Involved in the study	
☒	☐	ChIP-seq
☒	☐	Flow cytometry
☒	☐	MRI-based neuroimaging

Plants

Seed stocks	None to report.
Novel plant genotypes	None to report.
Authentication	None to report.
