# Supplementary figures and images for: Design-driven optimization of low-cost reagent formulations for reproducible and high-yielding cell-free gene expression
Source: Nat Commun. 2026 Mar 5;17:3478. doi: 10.1038/s41467-026-69605-8 (PMC13079839; doi:10.1038/s41467-026-69605-8)

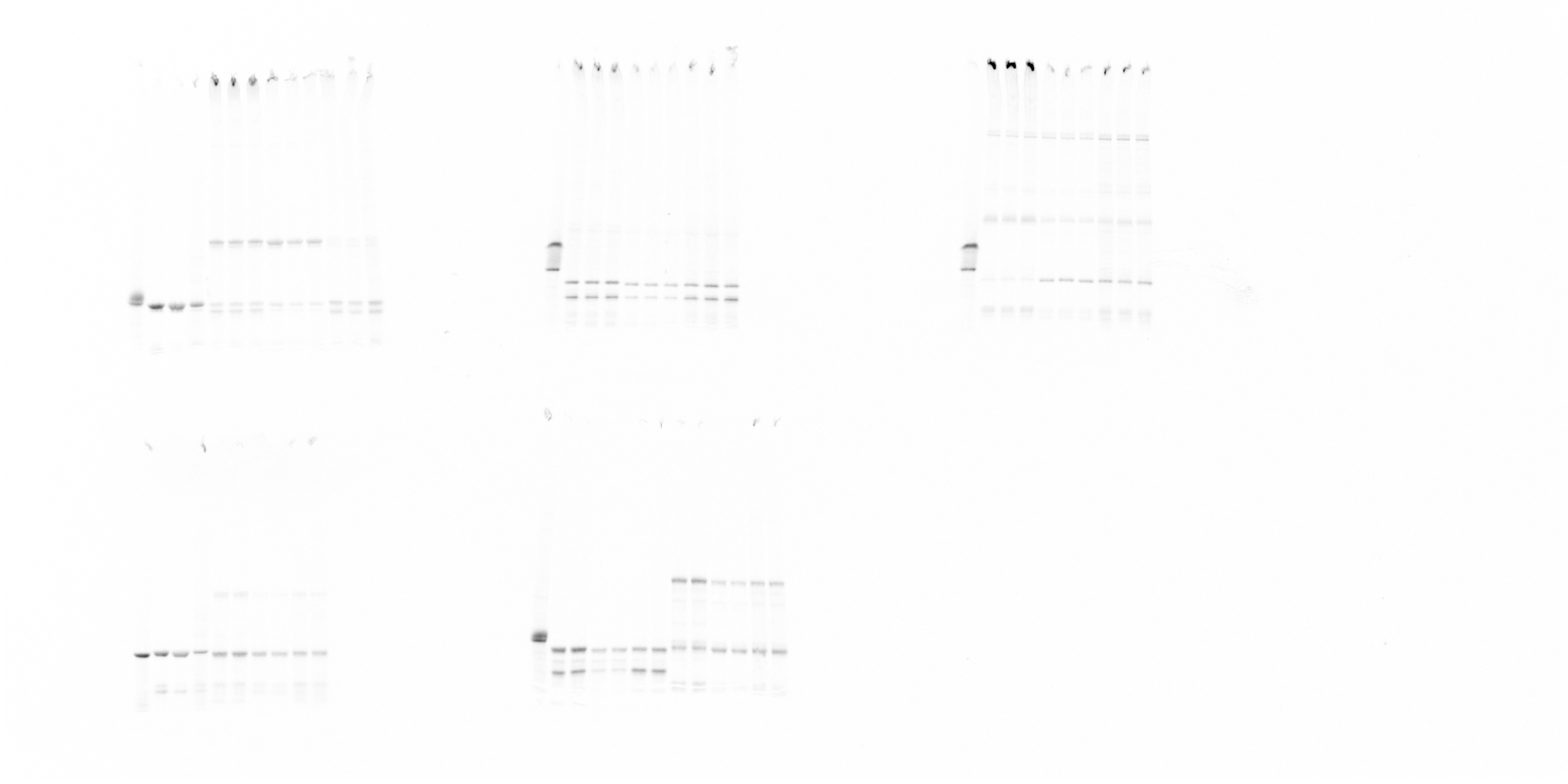

Supplement: Supplementary file 8 — Source data - Images [file 41467_2026_69605_MOESM8_ESM.zip › Source Data/Figs_S20-21_UneditedAutoradiogram.tif]

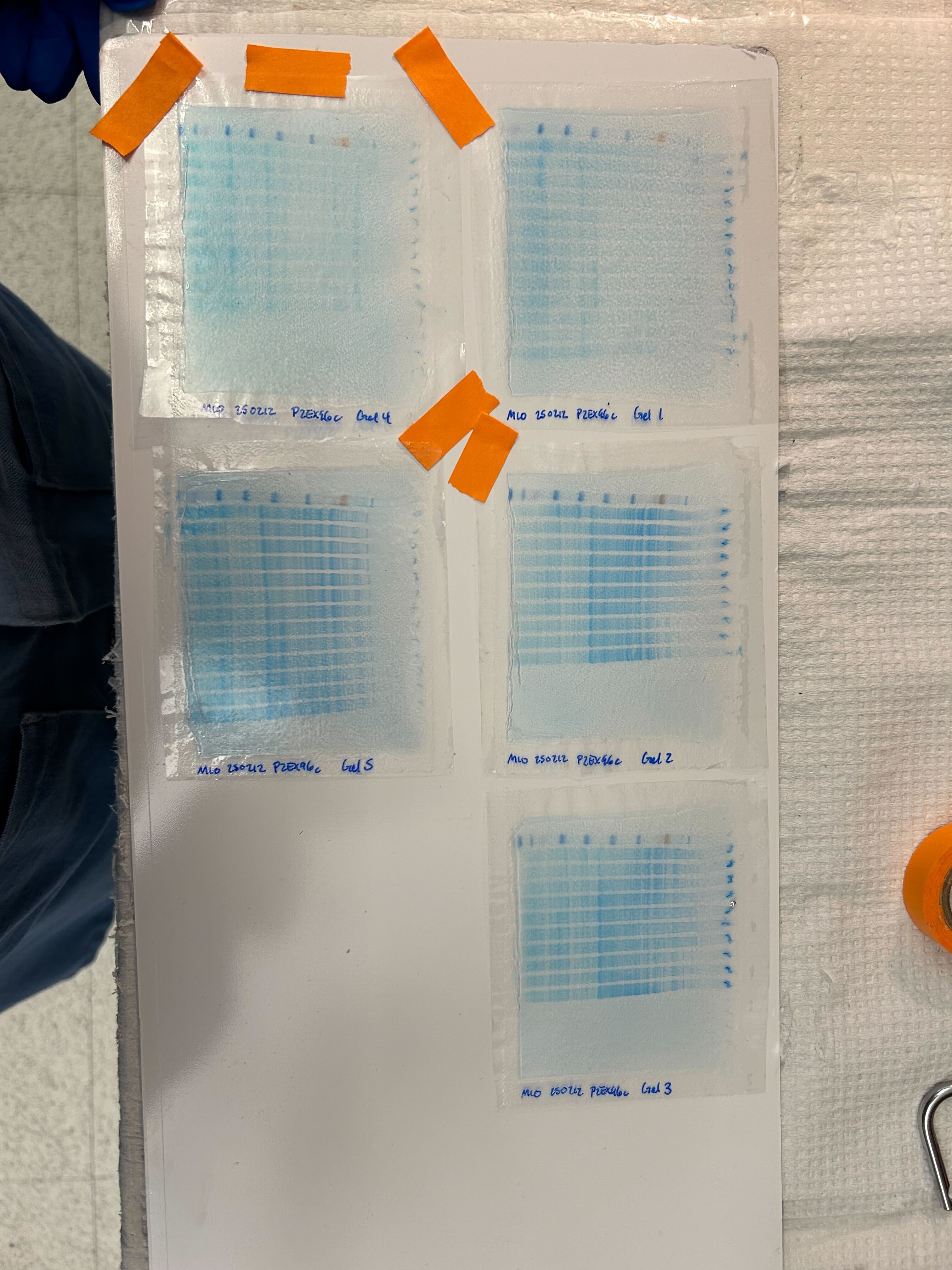

Supplement: Supplementary file 8 — Source data - Images [file 41467_2026_69605_MOESM8_ESM.zip › Source Data/Figs_S20-21_UneditedCoomassieStain.jpg]

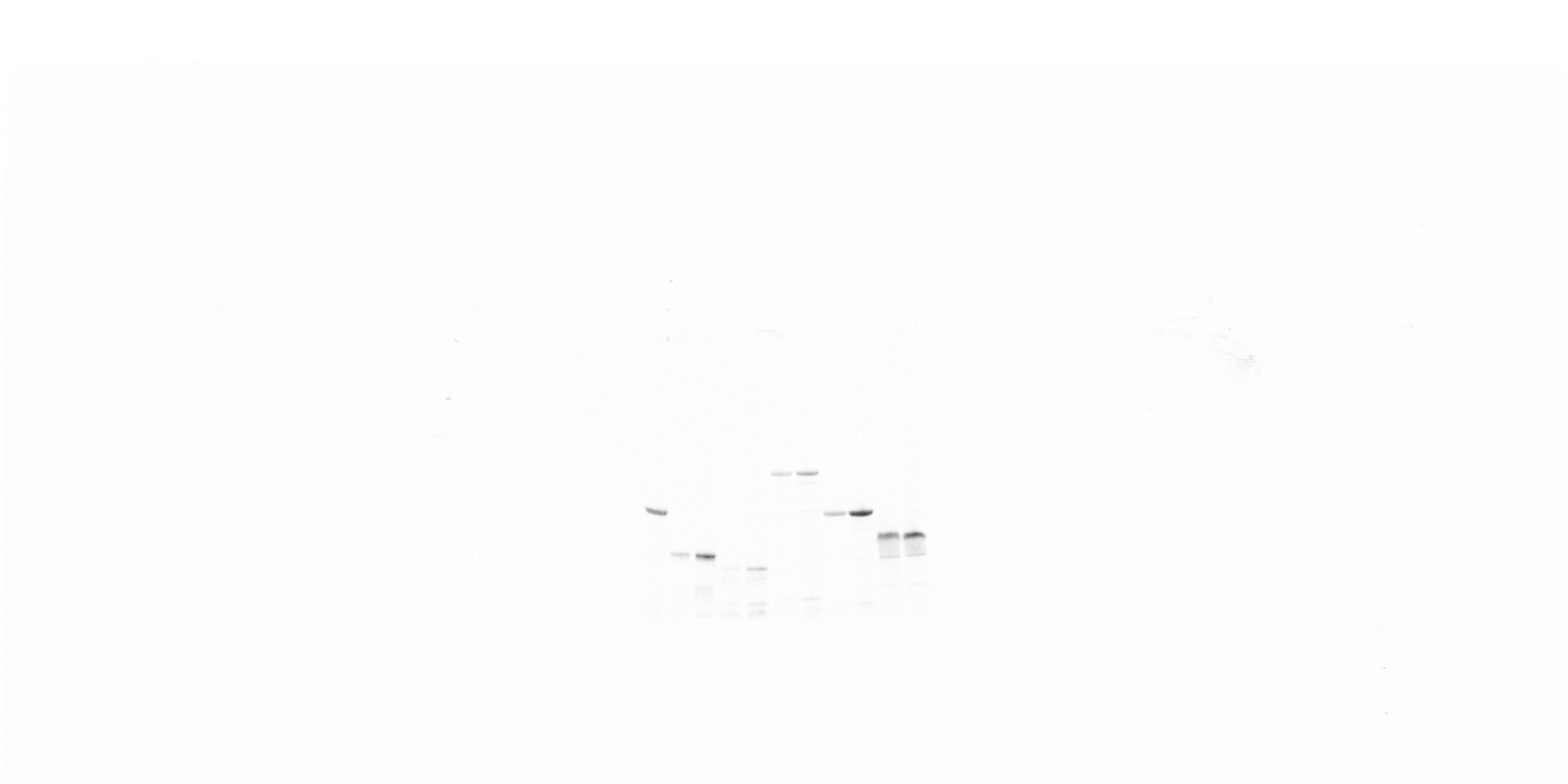

Supplement: Supplementary file 8 — Source data - Images [file 41467_2026_69605_MOESM8_ESM.zip › Source Data/Fig_S11_UneditedAutoradiogram.tif]

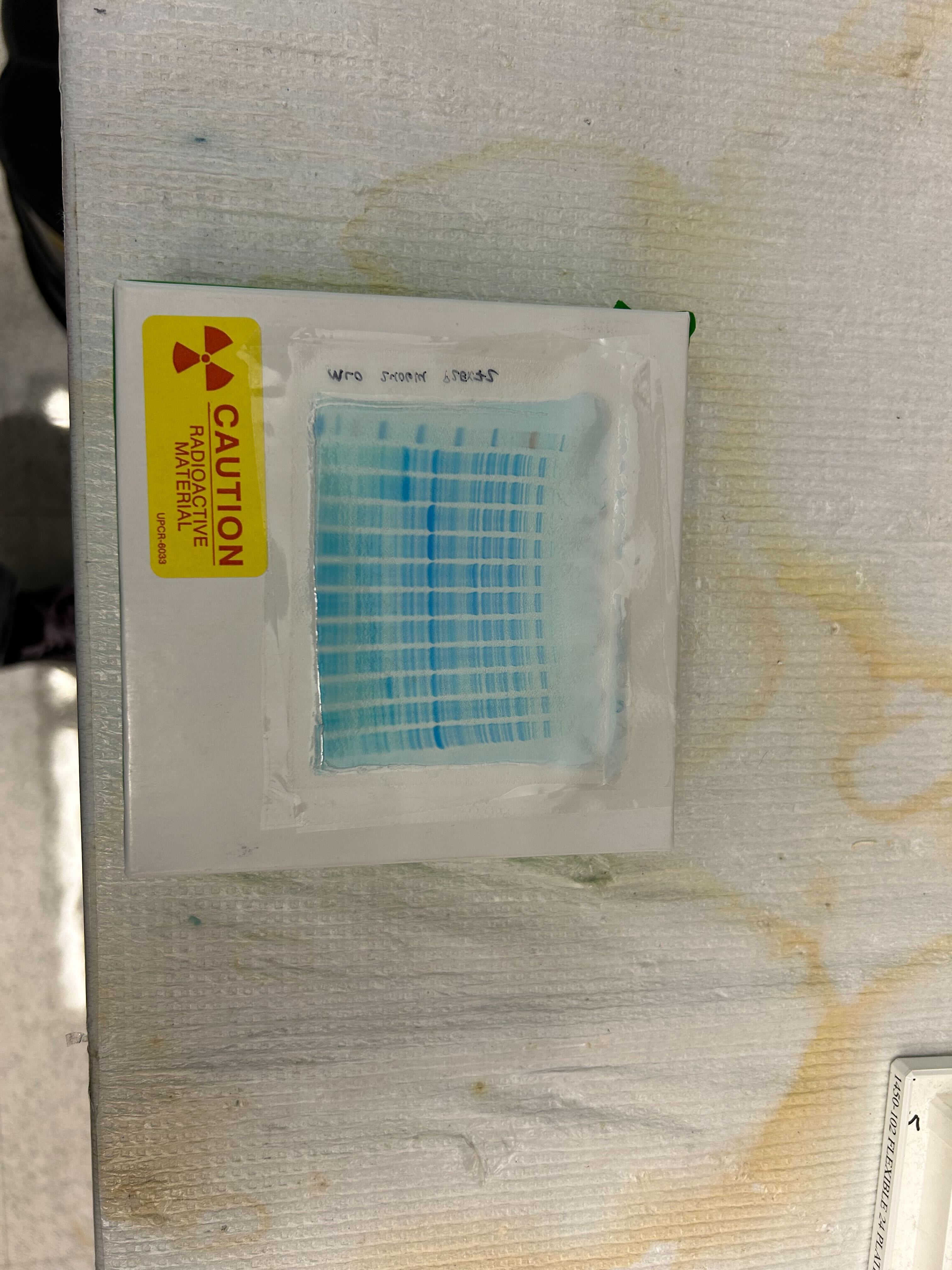

Supplement: Supplementary file 8 — Source data - Images [file 41467_2026_69605_MOESM8_ESM.zip › Source Data/Fig_S11_UneditedCoomassieStain.jpg]

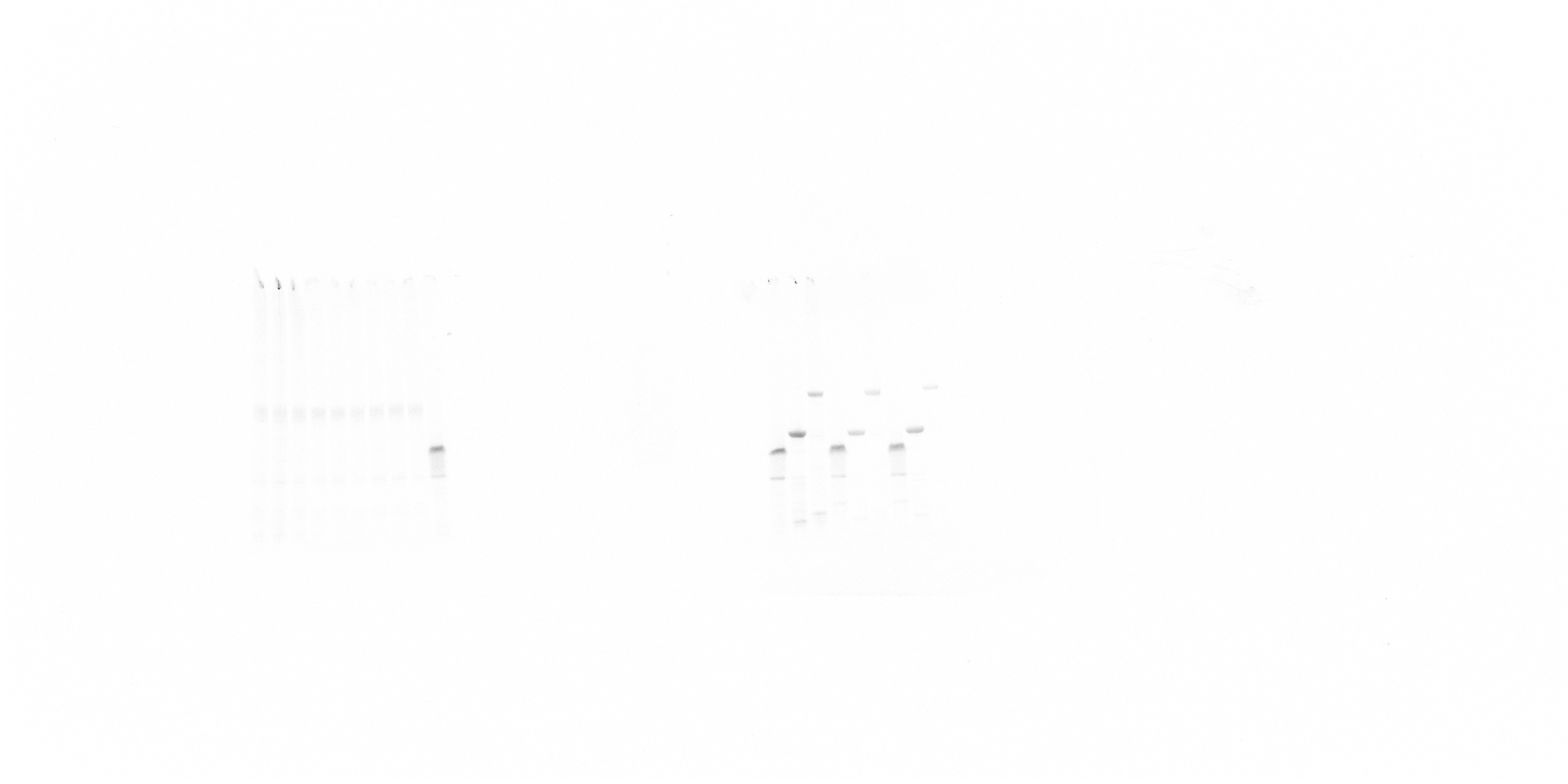

Supplement: Supplementary file 8 — Source data - Images [file 41467_2026_69605_MOESM8_ESM.zip › Source Data/Fig_S16a_UneditedAutoradiogram.tif]

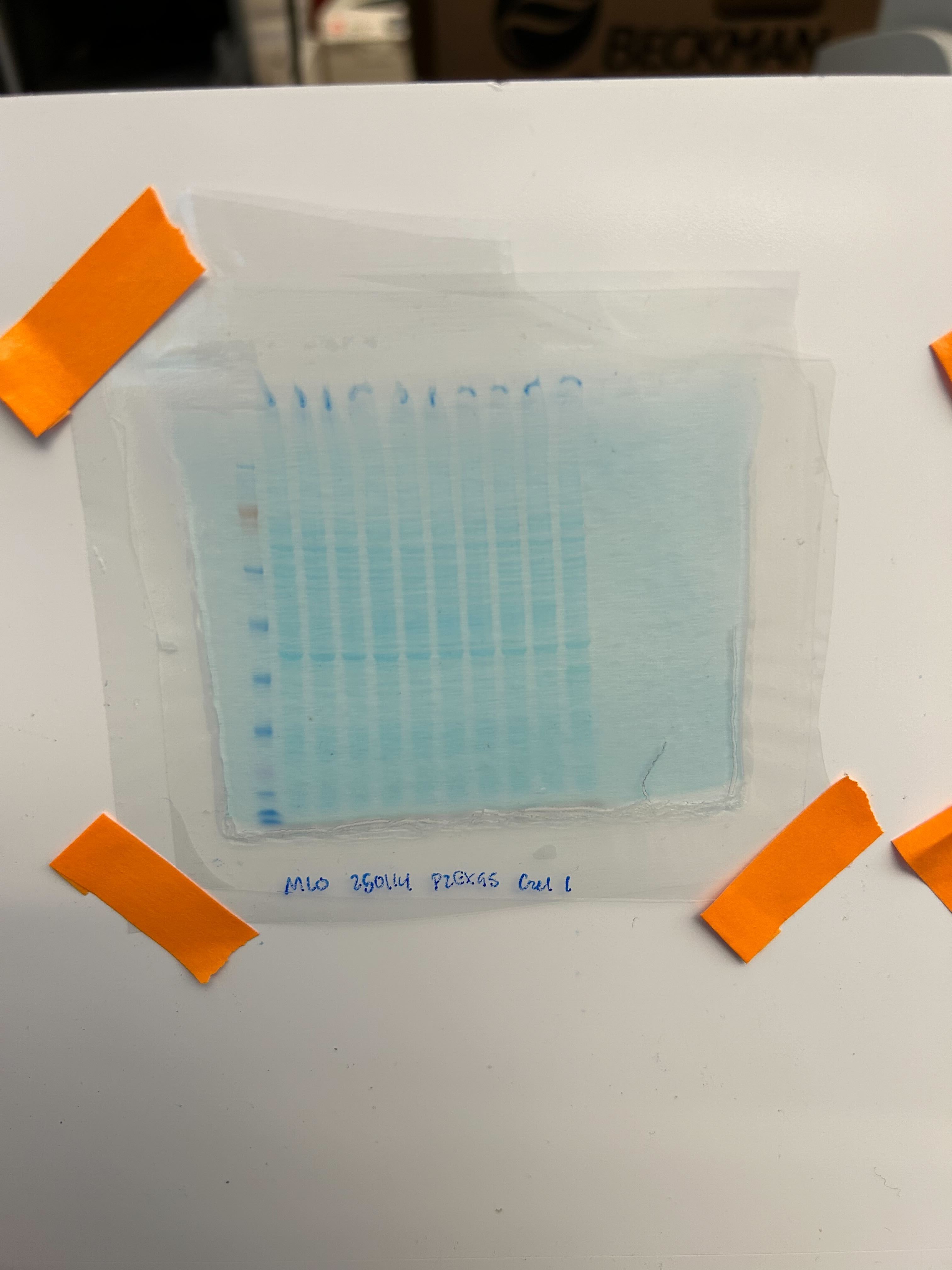

Supplement: Supplementary file 8 — Source data - Images [file 41467_2026_69605_MOESM8_ESM.zip › Source Data/Fig_S16a_UneditedCoomassieStain.jpg]

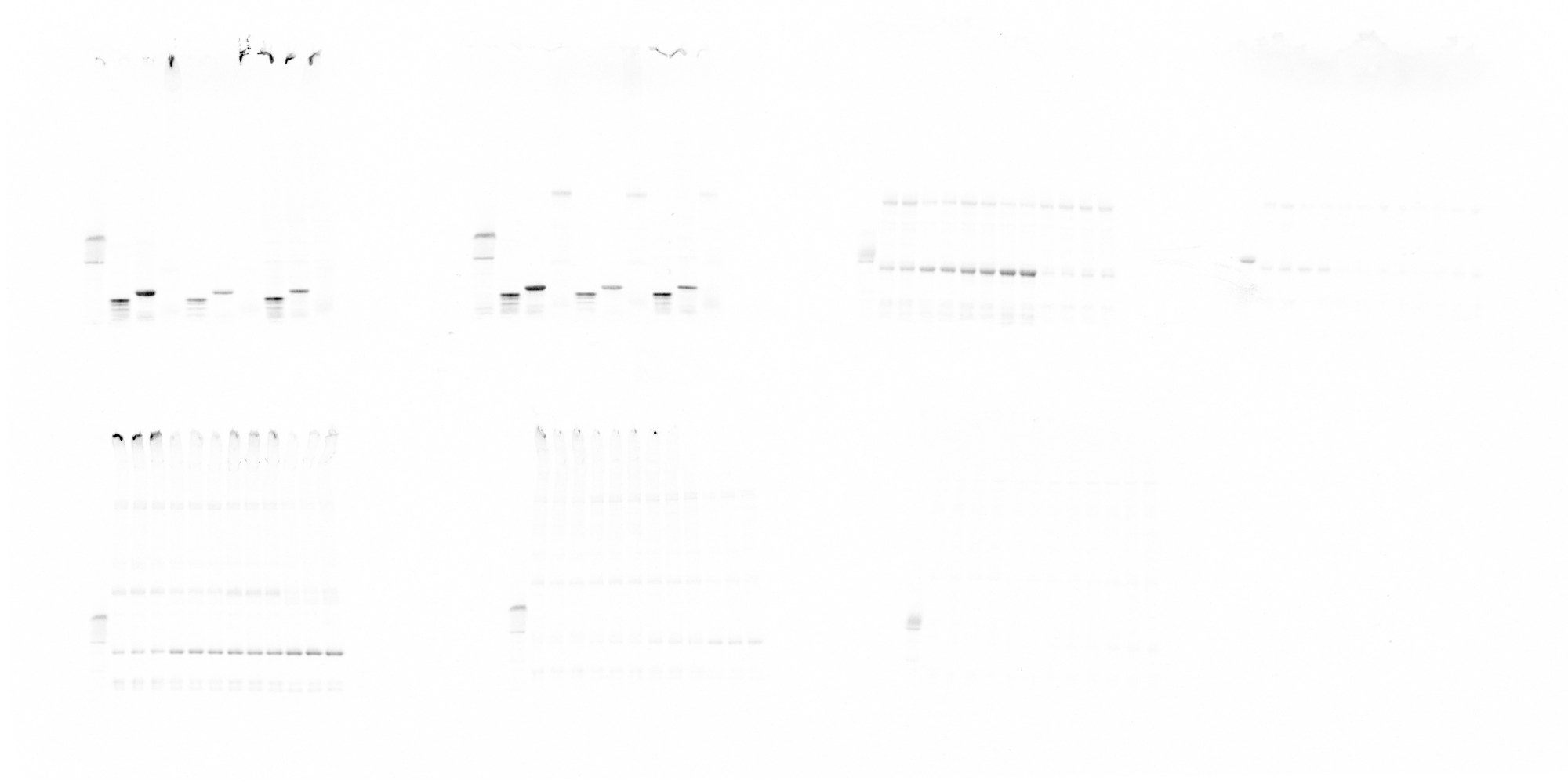

Supplement: Supplementary file 8 — Source data - Images [file 41467_2026_69605_MOESM8_ESM.zip › Source Data/Fig_S17_UneditedAutoradiograms.tif]

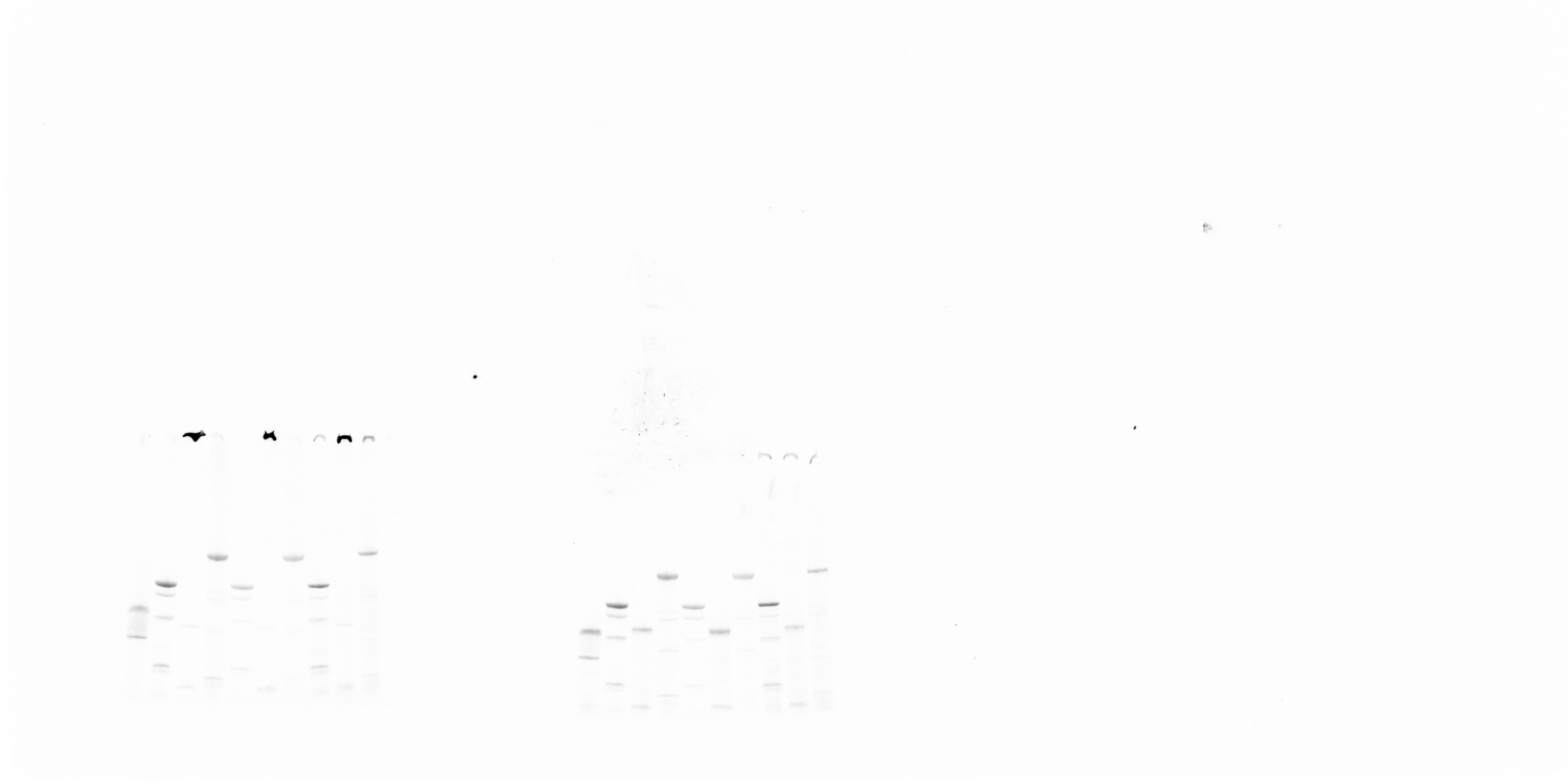

Supplement: Supplementary file 8 — Source data - Images [file 41467_2026_69605_MOESM8_ESM.zip › Source Data/Fig_S18_bottom_UneditedAutoradiogram.tif]

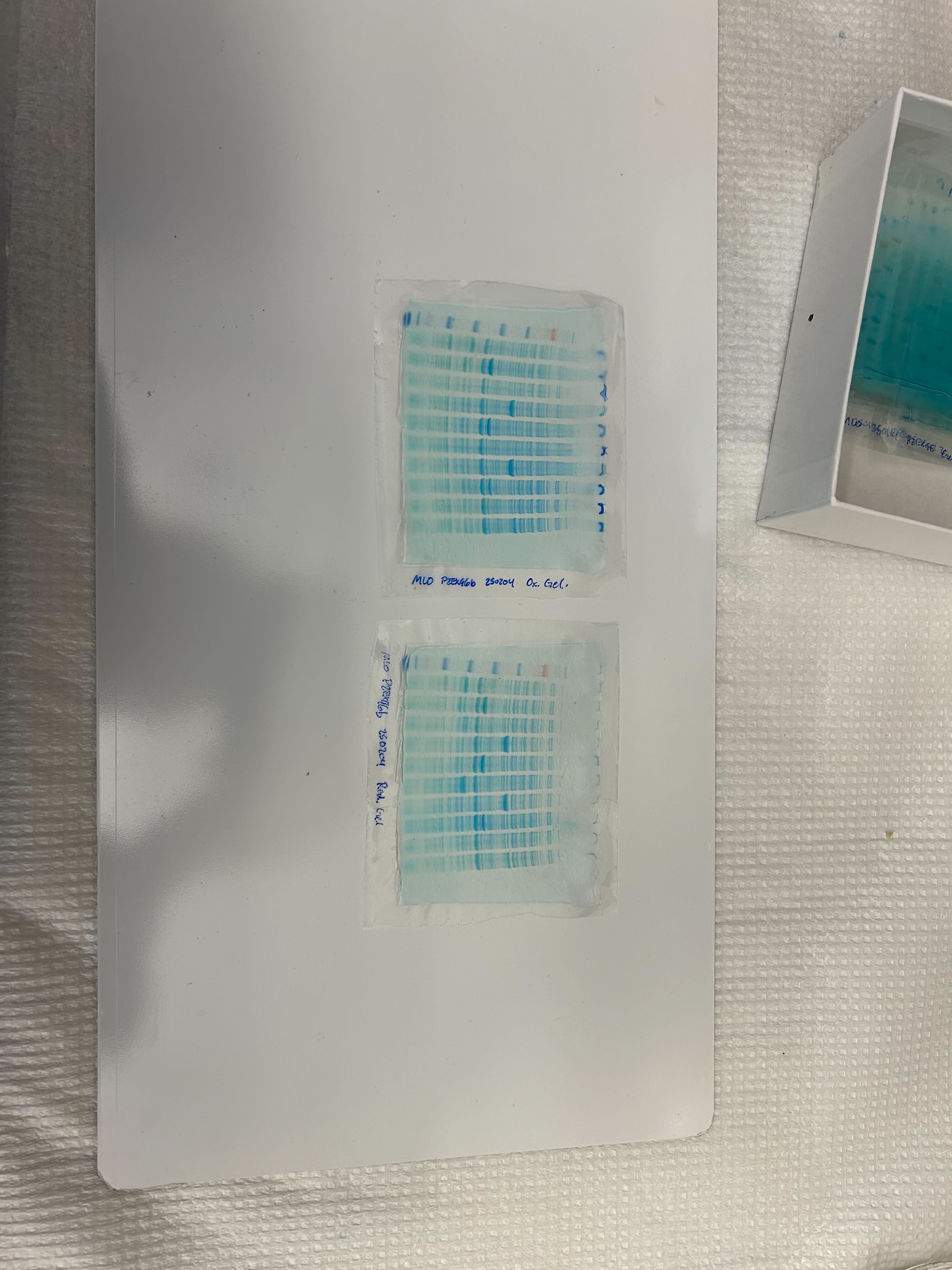

Supplement: Supplementary file 8 — Source data - Images [file 41467_2026_69605_MOESM8_ESM.zip › Source Data/Fig_S18_bottom_UneditedCoomassieStain.jpg]

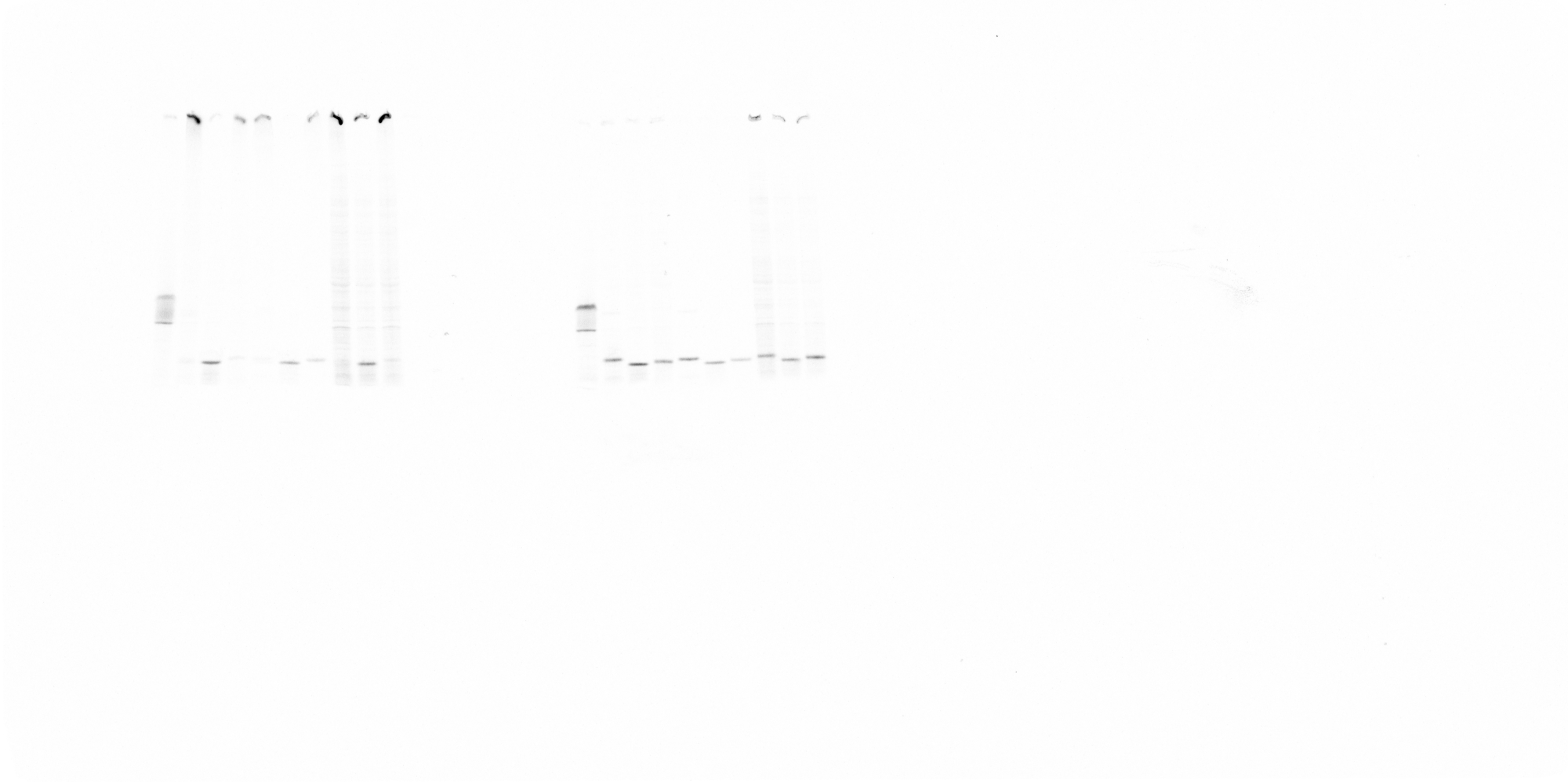

Supplement: Supplementary file 8 — Source data - Images [file 41467_2026_69605_MOESM8_ESM.zip › Source Data/Fig_S18_top_UneditedAutoradiograms.tif]

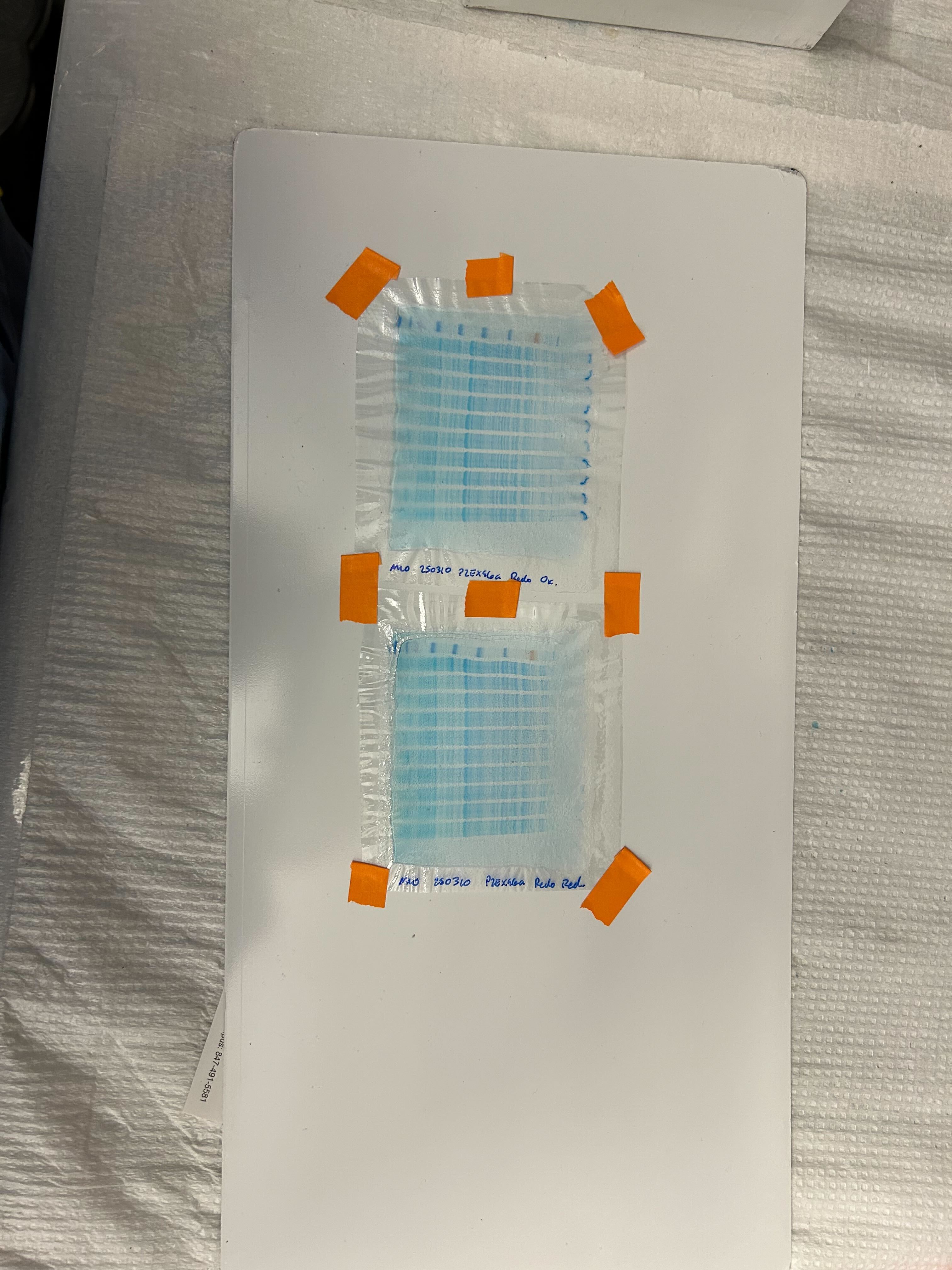

Supplement: Supplementary file 8 — Source data - Images [file 41467_2026_69605_MOESM8_ESM.zip › Source Data/Fig_S18_top_UneditedCoomassieStain.jpg]

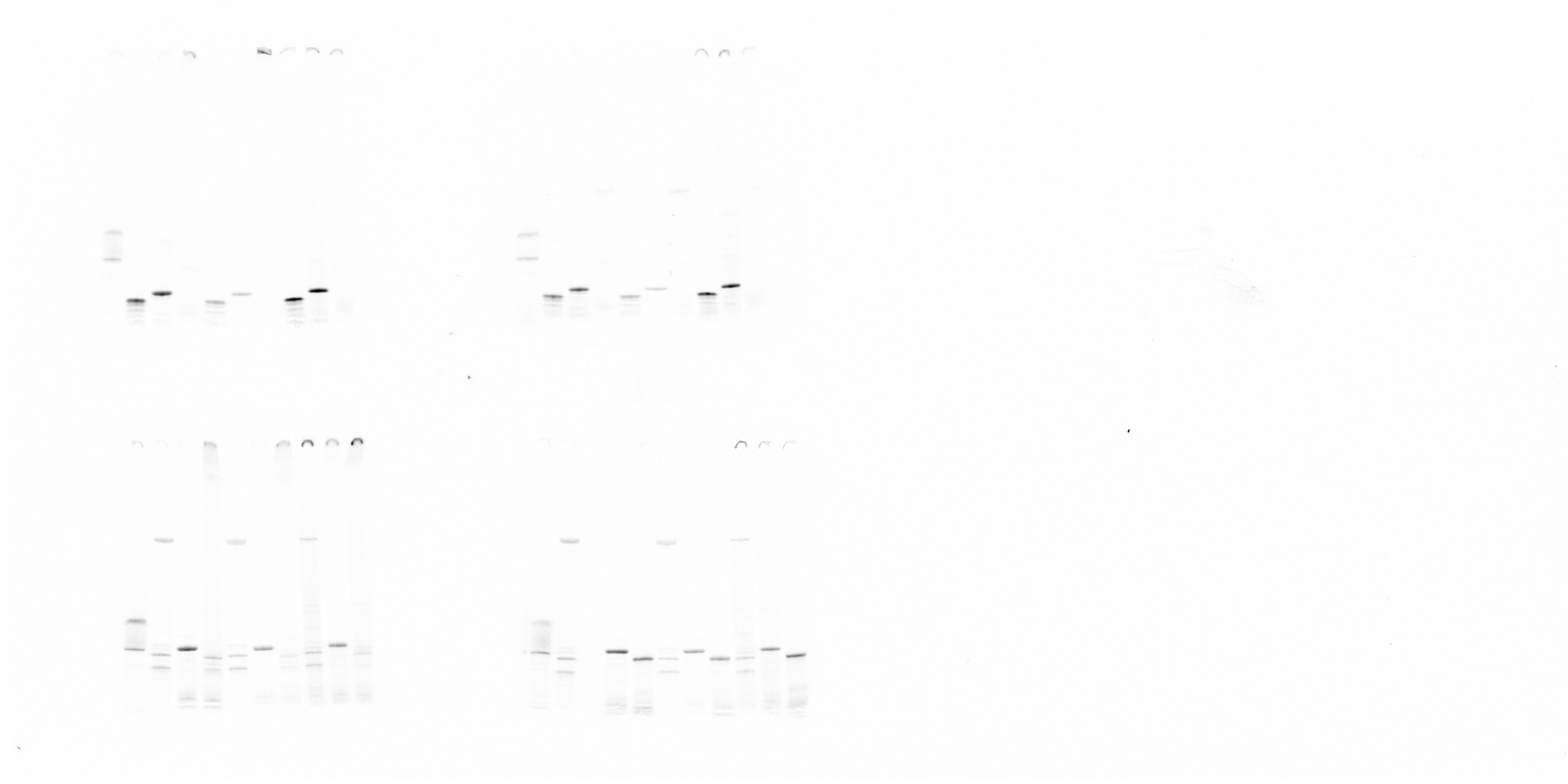

Supplement: Supplementary file 8 — Source data - Images [file 41467_2026_69605_MOESM8_ESM.zip › Source Data/Fig_S19_bottom_UneditedAutoradiogram.tif]

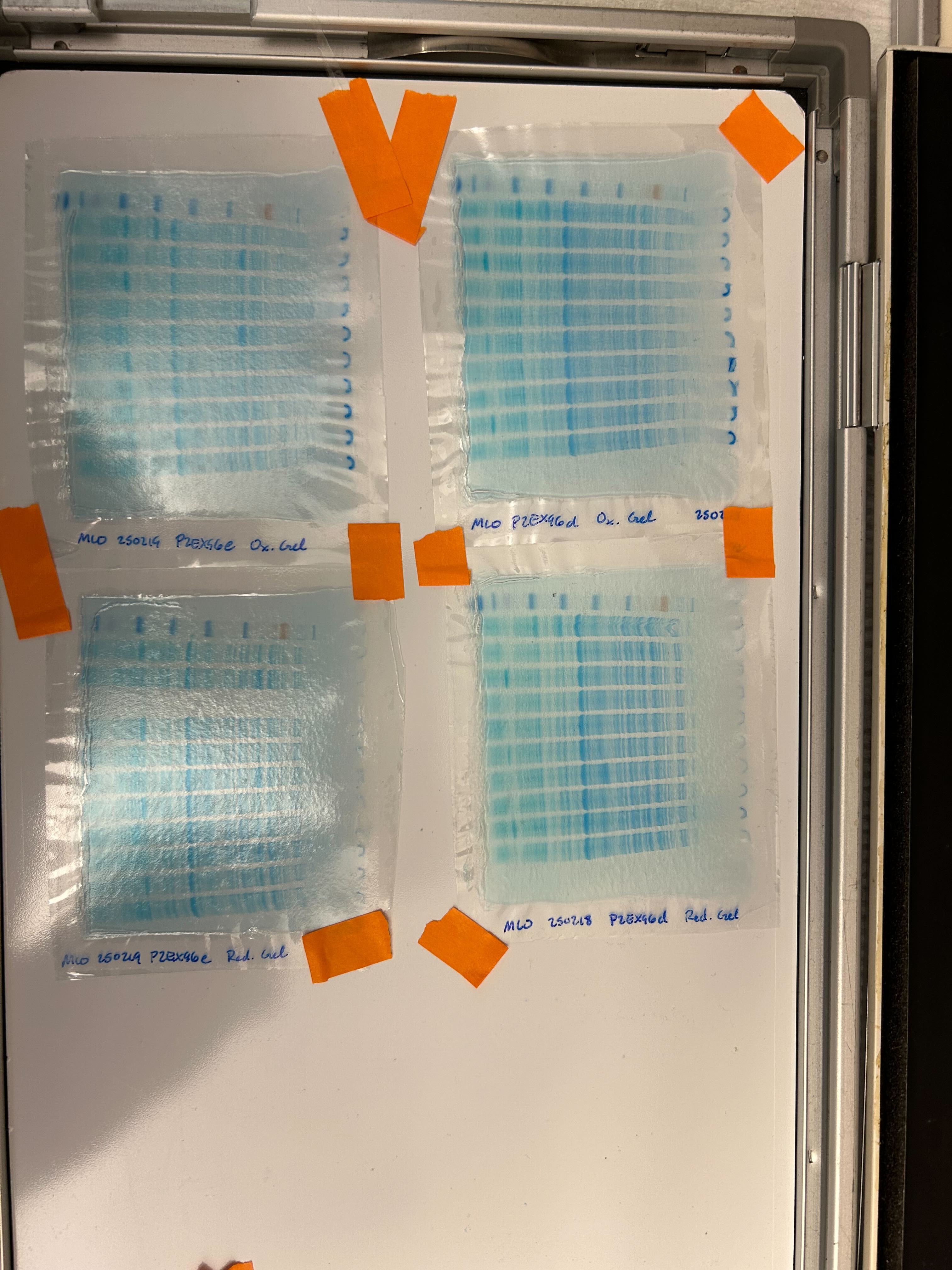

Supplement: Supplementary file 8 — Source data - Images [file 41467_2026_69605_MOESM8_ESM.zip › Source Data/Fig_S19_bottom_UneditedCoomassieStain.jpg]

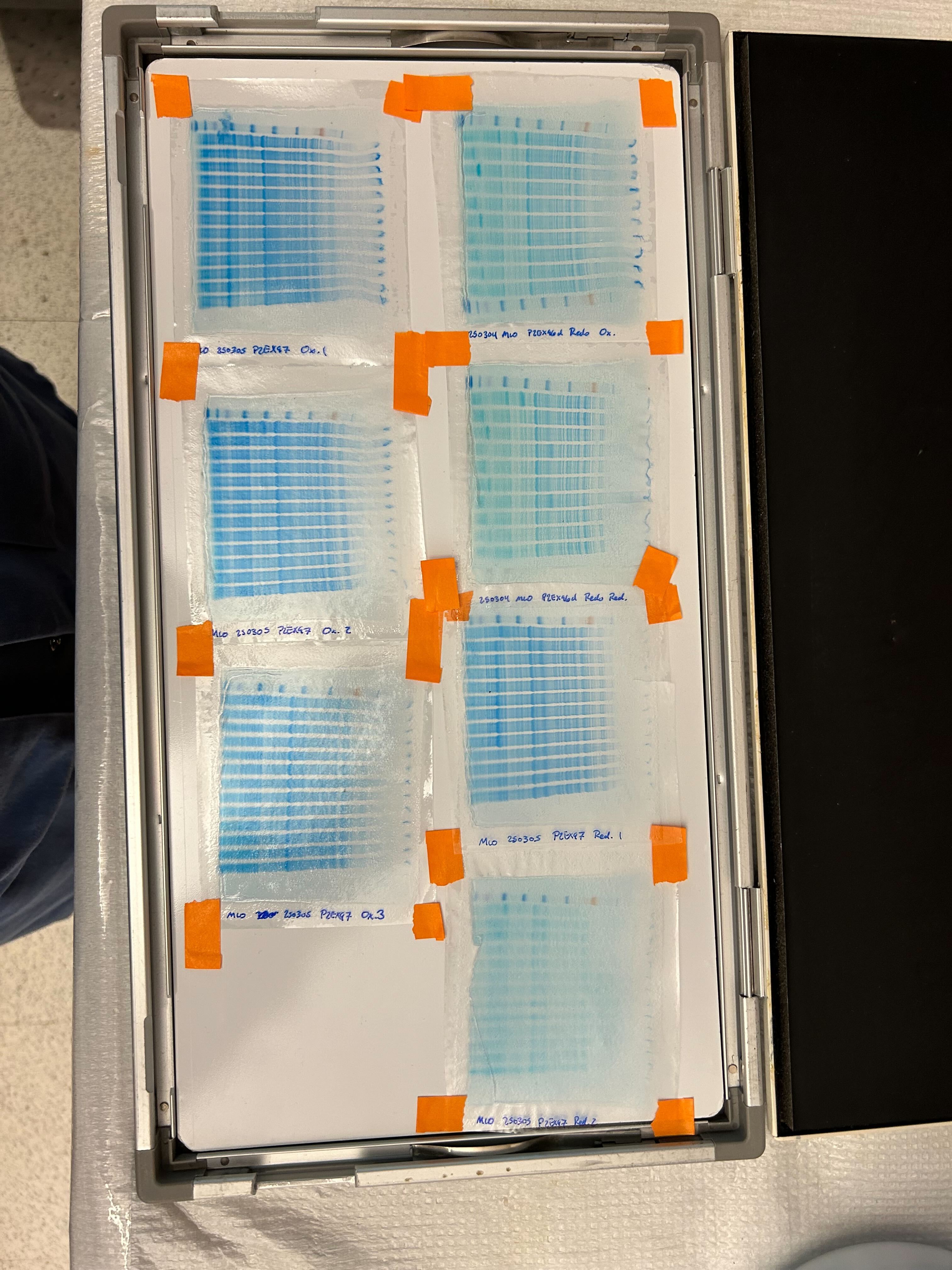

Supplement: Supplementary file 8 — Source data - Images [file 41467_2026_69605_MOESM8_ESM.zip › Source Data/Fig_S19_top_UneditedCoomassieStain.jpg]

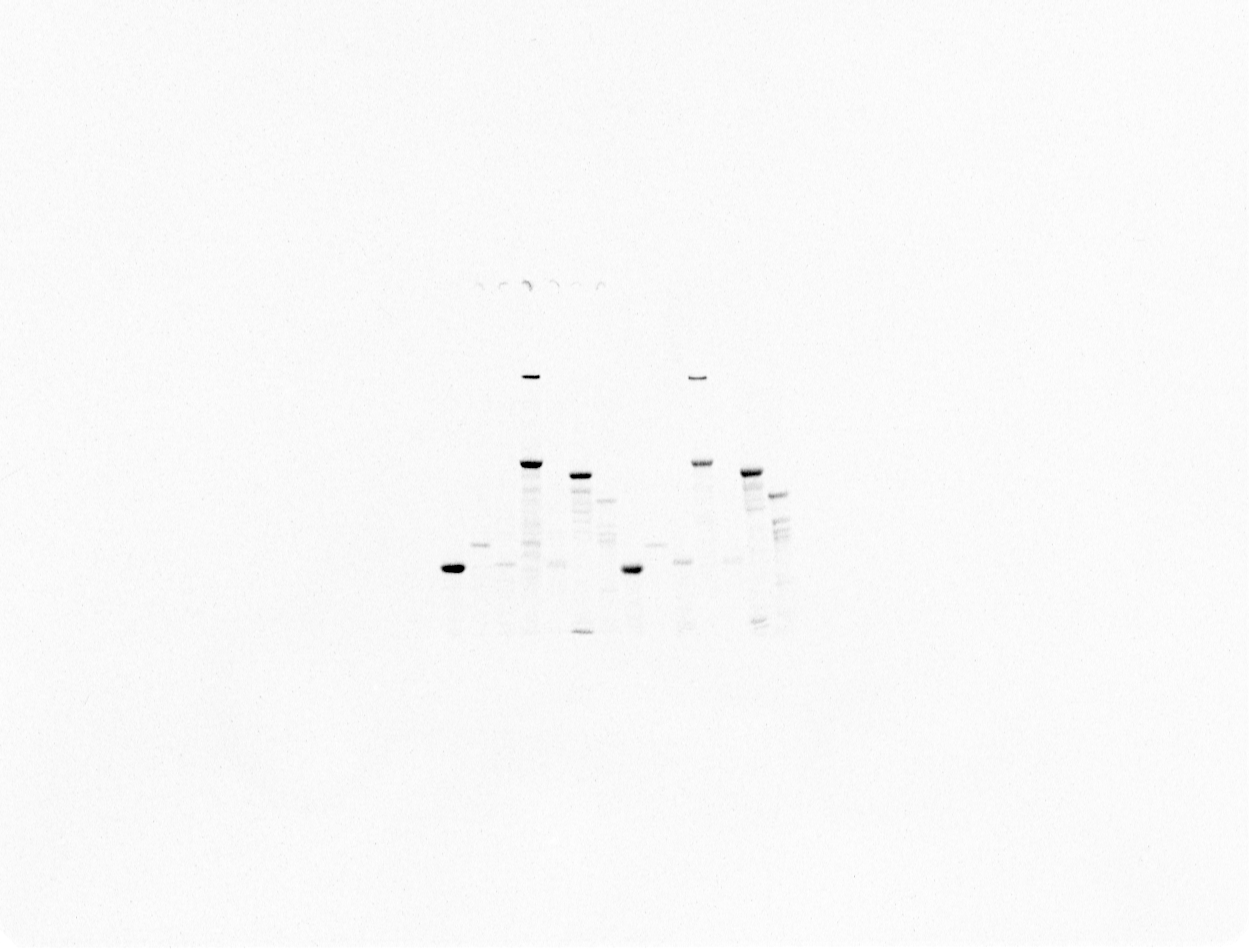

Supplement: Supplementary file 8 — Source data - Images [file 41467_2026_69605_MOESM8_ESM.zip › Source Data/Fig_S24_UneditedAutoradiogram.jpg]

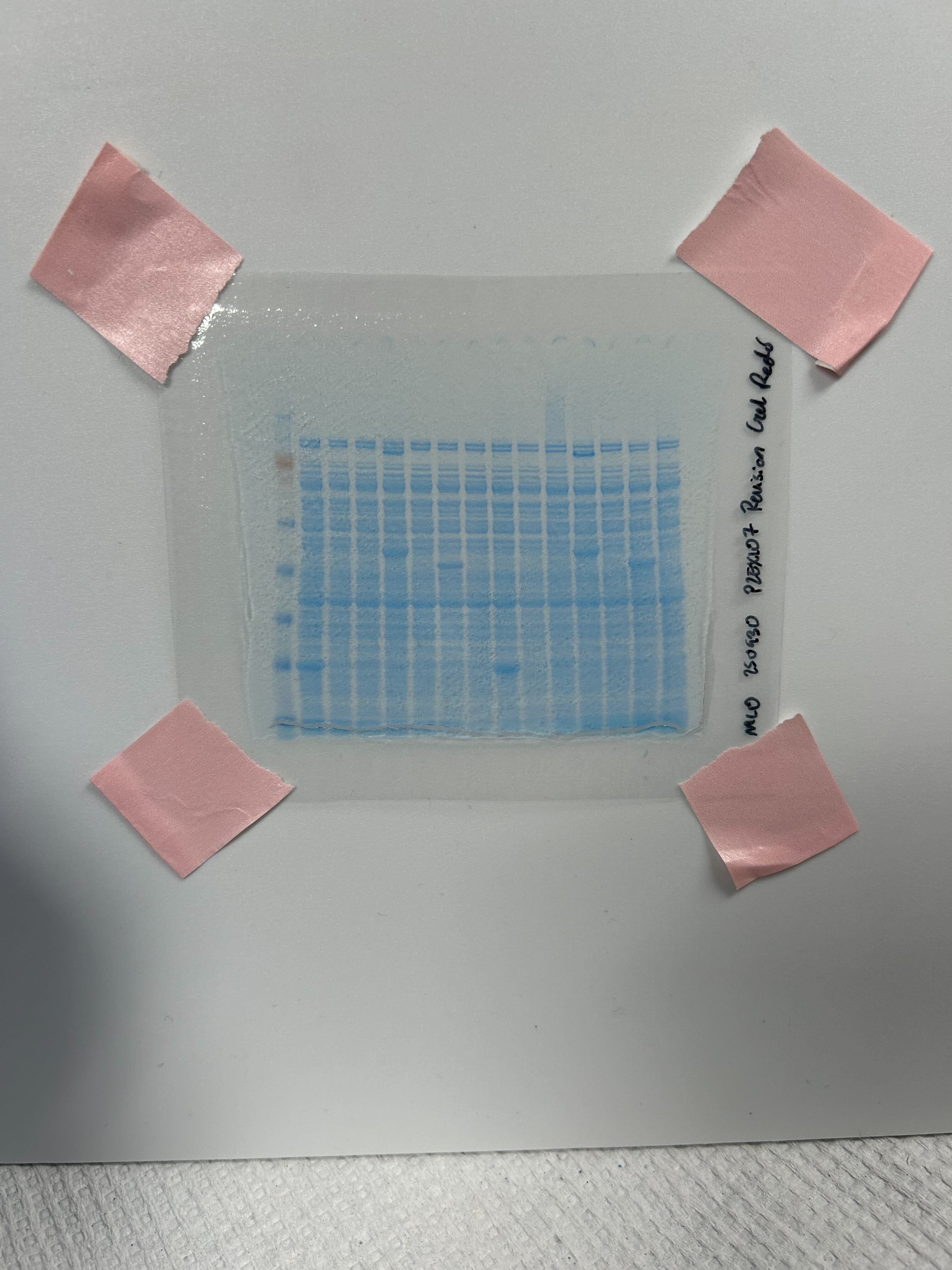

Supplement: Supplementary file 8 — Source data - Images [file 41467_2026_69605_MOESM8_ESM.zip › Source Data/Fig_S24_UneditedCoomassieStain.jpg]
